# PhenoMeNal: processing and analysis of metabolomics data in the cloud

**DOI:** 10.1093/gigascience/giy149

**Published:** 2018-12-07

**Authors:** Kristian Peters, James Bradbury, Sven Bergmann, Marco Capuccini, Marta Cascante, Pedro de Atauri, Timothy M D Ebbels, Carles Foguet, Robert Glen, Alejandra Gonzalez-Beltran, Ulrich L Günther, Evangelos Handakas, Thomas Hankemeier, Kenneth Haug, Stephanie Herman, Petr Holub, Massimiliano Izzo, Daniel Jacob, David Johnson, Fabien Jourdan, Namrata Kale, Ibrahim Karaman, Bita Khalili, Payam Emami Khonsari, Kim Kultima, Samuel Lampa, Anders Larsson, Christian Ludwig, Pablo Moreno, Steffen Neumann, Jon Ander Novella, Claire O'Donovan, Jake T M Pearce, Alina Peluso, Marco Enrico Piras, Luca Pireddu, Michelle A C Reed, Philippe Rocca-Serra, Pierrick Roger, Antonio Rosato, Rico Rueedi, Christoph Ruttkies, Noureddin Sadawi, Reza M Salek, Susanna-Assunta Sansone, Vitaly Selivanov, Ola Spjuth, Daniel Schober, Etienne A Thévenot, Mattia Tomasoni, Merlijn van Rijswijk, Michael van Vliet, Mark R Viant, Ralf J M Weber, Gianluigi Zanetti, Christoph Steinbeck

**Affiliations:** 1 Leibniz Institute of Plant Biochemistry, Stress and Developmental Biology, Weinberg 3, 06120 Halle (Saale), Germany; 2 School of Biosciences, University of Birmingham, Edgbaston, Birmingham, B15 2TT, United Kingdom; 3 Department of Computational Biology, University of Lausanne, Lausanne, Switzerland; 4 Swiss Institute of Bioinformatics, Lausanne, Switzerland; 5 Division of Scientific Computing, Department of Information Technology, Uppsala University, Sweden; 6 Department of Pharmaceutical Biosciences, Uppsala University, Box 591, 751 24 Uppsala, Sweden; 7 Department of Biochemistry and Molecular Biomedicine, Universitat de Barcelona; Centro de Investigación Biomédica en Red de Enfermedades Hepáticas y Digestivas (CIBEREHD), Instituto de Salud Carlos III (ISCIII), Spain; 8 Department of Computer Science, College of Engineering, Design and Physical Sciences, Brunel University, London, UK; 9 Department of Surgery & Cancer, Imperial College London, South Kensington, London, SW7 2AZ, United Kingdom; 10 Centre for Molecular Informatics, Department of Chemistry, University of Cambridge, Lensfield Road, Cambridge, CB21EW, United Kingdom; 11 Oxford e-Research Centre, Department of Engineering Science, University of Oxford, 7 Keble Road, OX1 3QG, Oxford, United Kingdom; 12 Institute of Cancer and Genomic Sciences, University of Birmingham, Edgbaston, Birmingham, B15 2TT, United Kingdom; 13 Netherlands Metabolomics Center, Leiden, 2333 CC, Netherlands; 14 Division of Systems Biomedicine and Pharmacology, Leiden Academic Centre for Drug Research (LACDR), Leiden University, Leiden, 2333 CC, The Netherlands; 15 European Molecular Biology Laboratory, European Bioinformatics Institute (EMBL-EBI), Wellcome Genome Campus, Hinxton, Cambridge CB10 1SD, United Kingdom; 16 Department of Medical Sciences, Clinical Chemistry, Uppsala University, 751 85 Uppsala, Sweden; 17 BBMRI-ERIC, Graz, Austria; 18 INRA, University of Bordeaux, Plateforme Métabolome Bordeaux-MetaboHUB, 33140 Villenave d'Ornon, France; 19 Department of Informatics and Media, Uppsala University, Box 513, 751 20 Uppsala, Sweden; 20 INRA - French National Institute for Agricultural Research, UMR1331, Toxalim, Research Centre in Food Toxicology, Toulouse, France; 21 Department of Epidemiology and Biostatistics, School of Public Health, Imperial College London, St. Mary's Campus, Norfolk Place, W2 1PG, London, United Kingdom; 22 National Bioinformatics Infrastructure Sweden, Uppsala University, Uppsala, Sweden; 23 Institute of Metabolism and Systems Research (IMSR), University of Birmingham, Edgbaston, Birmingham, B15 2TT, United Kingdom; 24 German Centre for Integrative Biodiversity Research (iDiv) Halle-Jena-Leipzig, Deutscher Platz 5e, 04103 Leipzig, Germany; 25 Distributed Computing Group, CRS4, Pula, Italy; 26 CEA, LIST, Laboratory for Data Analysis and Systems’ Intelligence, MetaboHUB, Gif-Sur-Yvette F-91191, France; 27 Magnetic Resonance Center (CERM) and Department of Chemistry, University of Florence and CIRMMP, 50019 Sesto Fiorentino, Florence, Italy; 28 ELIXIR-NL, Dutch Techcentre for Life Sciences, Utrecht, 3503 RM, Netherlands; 29 Phenome Centre Birmingham, University of Birmingham, Edgbaston, Birmingham, B15 2TT, United Kingdom; 30 Cheminformatics and Computational Metabolomics, Institute for Analytical Chemistry, Lessingstr. 8, 07743 Jena, Germany

**Keywords:** metabolomics, data analysis, e-infrastructures, NMR, mass spectrometry, computational workflows, galaxy, cloud computing, standardization, statistics

## Abstract

**Background:**

Metabolomics is the comprehensive study of a multitude of small molecules to gain insight into an organism's metabolism. The research field is dynamic and expanding with applications across biomedical, biotechnological, and many other applied biological domains. Its computationally intensive nature has driven requirements for open data formats, data repositories, and data analysis tools. However, the rapid progress has resulted in a mosaic of independent, and sometimes incompatible, analysis methods that are difficult to connect into a useful and complete data analysis solution.

**Findings:**

PhenoMeNal (Phenome and Metabolome aNalysis) is an advanced and complete solution to set up Infrastructure-as-a-Service (IaaS) that brings workflow-oriented, interoperable metabolomics data analysis platforms into the cloud. PhenoMeNal seamlessly integrates a wide array of existing open-source tools that are tested and packaged as Docker containers through the project's continuous integration process and deployed based on a kubernetes orchestration framework. It also provides a number of standardized, automated, and published analysis workflows in the user interfaces Galaxy, Jupyter, Luigi, and Pachyderm.

**Conclusions:**

PhenoMeNal constitutes a keystone solution in cloud e-infrastructures available for metabolomics. PhenoMeNal is a unique and complete solution for setting up cloud e-infrastructures through easy-to-use web interfaces that can be scaled to any custom public and private cloud environment. By harmonizing and automating software installation and configuration and through ready-to-use scientific workflow user interfaces, PhenoMeNal has succeeded in providing scientists with workflow-driven, reproducible, and shareable metabolomics data analysis platforms that are interfaced through standard data formats, representative datasets, versioned, and have been tested for reproducibility and interoperability. The elastic implementation of PhenoMeNal further allows easy adaptation of the infrastructure to other application areas and ‘omics research domains.

## Findings

### Background

The field of metabolomics has seen remarkable progress over the last decade and has enabled fascinating discoveries in many different research areas. Metabolomics is the study of small molecules in organisms that can reveal detailed insights into metabolic biochemistry, e.g., changes in concentrations of specific molecules, metabolic fluxes between cells or compartments, identification of molecules that are involved in the pathogenesis of a disease, and the study of the biochemical phenotype of animals, plants, and even soil microorganisms [1–3].

The principal metabolomics technologies of mass spectrometry (MS) and nuclear magnetic resonance spectroscopy (NMR) typically generate large datasets that require computationally intensive analyses [4]. Biomedical investigations can involve large cohorts with many thousands of metabolite profiles and can produce hundreds of gigabytes of data [5–8]. With such large datasets, processing becomes impracticable and unmanageable on commodity hardware. Cloud computing can offer a solution by enabling the outsourcing of calculations from local workstations to scalable cloud data centers, with the possibility to allocate thousands of central processing unit (CPU) cores simultaneously. Furthermore, cloud computing allows for resources to be instantiated on-demand (CPUs, random access memory, network, storage) and allows access to computational tools in the form of microservices that can dynamically grow or shrink.

MS and NMR data processing usually involves selection of parameters (that are often specific to the analytical instrumentation), algorithmic peak detection, peak alignment and grouping, annotation of putative compounds, and extensive statistical analyses [9, 10]. Many open-source tools have been developed that address these different steps in data processing and analysis. These tools, however, usually come with their own software dependencies, resource requirements, and scripting languages. As a consequence, configuring and running them is often complicated, especially for researchers who are untrained in computer science [4]. Furthermore, many tools require users to input parameters that can significantly affect results and performance, and reporting of these parameters is not always clear [11].

A number of infrastructures and integration efforts have been initiated in the past five years, including metabolomics data repositories with a global scope [6, 12], platforms for reproducible workflow analysis [13, 14], as well as initiatives to integrate and coordinate data standards [15]. Simultaneously, multiple networks of service centers such as the international Phenome Centers [16] and MetaboHub [17] have formed with the goal to facilitate the acquisition, processing, and analysis of metabolomics data [6–8] at ever increasing scales.

Currently, several web-based metabolomics data processing platforms are available. XCMSOnline provides a platform based on XCMS for downstream data analysis, visualization, data sharing, and access to Metlin to facilitate metabolite identification and pathway analysis [18]. MetaboAnalyst presents a wide variety of data processing and analysis tools including statistical analysis, time-series analysis, functional analysis, and pathway analysis [19]. Workflow4Metabolomics is based on Galaxy and provides various metabolomics processing workflows, including NMR [13, 20]. These common tools for analyzing metabolomics data provide web-based graphical user interfaces (GUIs) with different functionality.

Here, we present PhenoMeNal (Phenome and Metabolome aNalysis), a unique, easy-to-use, complete, robust, and performant cloud e-infrastructure that provides a large suite of standardized and interoperable metabolomics data processing tools as a complete data analysis solution. In contrast to current metabolomics processing platforms, PhenoMeNal provides Infrastructure-as-a-Service (IaaS) and seamlessly integrates a wide array of existing open-source tools.

**Figure 1:**
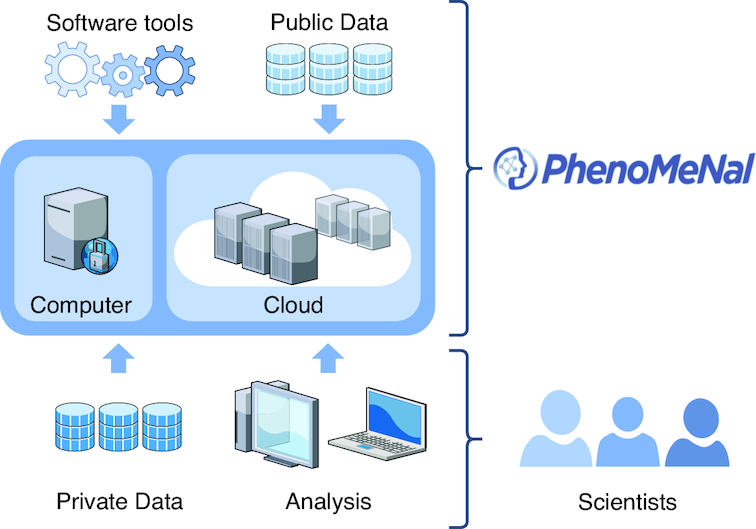
Conceptual design of the PhenoMeNal cloud e-infrastructure, which brings compute to the data for any large number of data scientists.

A major advantage over other platforms is that PhenoMeNal make it possible to instantiate many different services in the cloud and provides a number of standardized, automated, and published analysis workflows in the user interfaces Galaxy, Jupyter, Luigi, and Pachyderm (Fig. 1). Moreover, the PhenoMeNal e-infrastructure can be easily deployed onto public and private cloud environments and can be configured elastically to fit into any cloud-based environment, thus enabling scalable and cost-effective high-performance metabolomics data analysis in a way that hides the technical complexity from the user. PhenoMeNal further facilitates reproducible analyses through automated, sharable, and citable workflows.

### Overview

**Figure 2:**
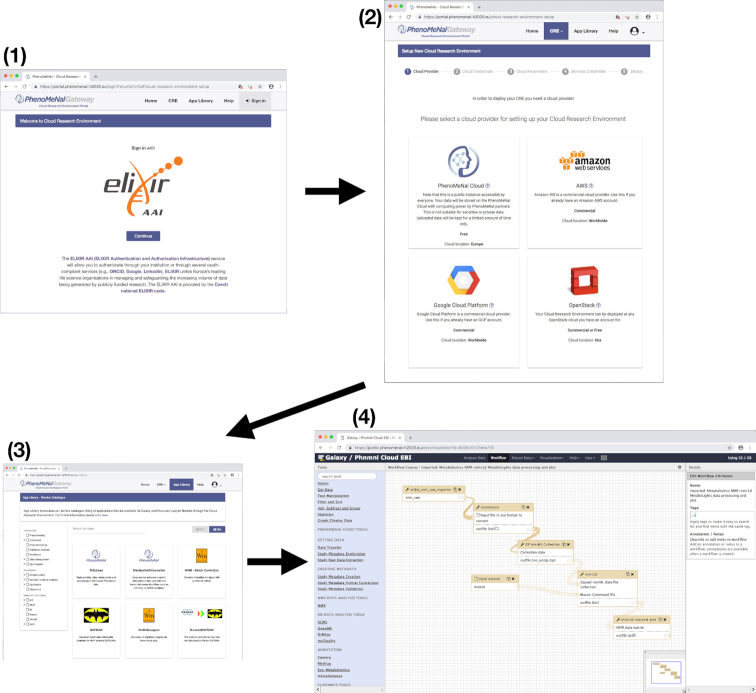
Screenshots of creating and using the PhenoMeNal cloud e-infrastructure. First, log in with ELIXIR to the cloud research environment (CRE) portal. Second, select a public or private cloud provider. After entering cloud credentials and setting up parameters in the dedicated portal, the deployment of the PhenoMeNal e-infrastructure into the cloud environment can be made. Third, in the PhenoMeNal Portal app library there are several services ready to be deployed and used in the set-up infrastructure. Fourth, dedicated web services such as Galaxy are readily available in the cloud e-infrastructure. All steps can be operated from an easy-to-use web interface that is accessible from any standard web browser.

The features of the PhenoMeNal e-infrastructure are encapsulated as a cloud research environment (CRE). The PhenoMeNal CRE can be instantiated on major commercial public cloud providers, including Amazon web services (AWS) and Google cloud platform (GCP), as well as OpenStack-based private clouds and in custom environments. Technical complexity is hidden from the users, simplifying setting up the cloud infrastructure for administrators (Fig. 2).

From a web-based portal, users can deploy the CRE, which includes several web services and software tools (Fig. 2). Data can be processed directly in the e-infrastructure without the need to install additional software. Scientific workflows can be executed via user-friendly web-based platforms such as Galaxy, as well as programmatic interfaces and notebooks. Each service has been supplied with a rich source of documentation and training material to assist researchers.

#### The PhenoMeNal Portal

The PhenoMeNal Portal [21] allows users to deploy, manage, and delete PhenoMeNal CREs simply through a web interface. Deployments to major commercial cloud platforms (AWS and GCP) as well as OpenStack, an open-source cloud platform, can be made using an easy-to-follow wizard (Fig. 2). OpenStack deployments can be deployed behind clinical firewalls, which is especially pertinent when dealing with sensitive (i.e., patient) data.

The PhenoMeNal public instance allows users to test-run a CRE without the need to deploy on a cloud platform. It can be deployed and accessed through the portal. Once credentials for users have been generated, analyses can be run through a Galaxy instance containing the tools and workflows present in any deployed CRE. The portal also includes user and developer documentation, workflow tutorials, and links to training videos.

#### Scientific workflows

A scientific workflow is a set of computational steps that are carried out to process and analyze data [22]. Usually, a workflow is comprised of several linked software tools that are each executed during a particular step of the workflow. In order to manage and automate scientific workflows, in PhenoMeNal the well-established dedicated workflow management system Galaxy can be deployed, which presents the user with an easy-to-use graphical user interface as well as providing a programmatic interface [20, 23]. Galaxy facilitates collaborative exchange, reproducibility, and traceability of data analysis by enabling users to share entire workflows and analysis histories [24]. In addition to Galaxy, programmatic executable notebooks (Jupyter) and the workflow tools exposed as programmatic interfaces Luigi and Pachyderm are also supported [25].

In order to cover typical use cases in metabolomics and to illustrate the usage and applicability of given analytical pipelines and software tools, five representative scientific workflows are available in the PhenoMeNal Galaxy (Table 1), each having different computational demands and purposes. More than 250 individual modules have been integrated in Galaxy (see the subsection Scientific Workflows in the Methods section).

**Table 1:** List of workflows that are representative for their respective metabolomics domains (identification in NMR, Fluxomics, Annotation, and identification in MS and eco-metabolomics)

Workflow name	Description	Reference
1D NMR	Processes 1D NMR experiments from raw data to a data matrix required for visualization and statistical analysis, building on nmrML and NMRProcFlow. The automatic workflow is based on the MTBLS1 dataset, describing urinary changes in type 2 diabetes in humans.	[26, 27, 28]
Fluxomics	Quantifies steady-state fluxes following ^13^C metabolic flux analysis. The workflow was first based on the analysis of the MTBLS412 dataset with 13C tracer data of human umbilical vein endothelial cells under hypoxia.	[29, 30]
LC-MS/MS	Processes, quantifies, and annotates/identifies features in mass spectra using MetFrag — a tool that annotates molecules from compound databases of tandem mass spectrometry (MS/MS) spectra. The workflow is based on MTBLS558.	[31, 32, 33]
Univariate and Multivariate Statistics	Applies univariate and multivariate statistical analysis and illustrates how datasets may be explored, enabling the identification of variables of interest and the construction of predictive models. The workflow is based on MTBLS404.	[13, 34]
Eco-Metabolomics	Implementation of a resource demanding metabolomics use case in ecology, used in large field experiments to describe interactions between different species of organisms in remarkable detail. The workflow is based on MTBLS520.	[35]
ISA-Create-Validate-Upload	A workflow to create Investigation, Study, and Assay data model framework-compliant metadata files based on study design information, augmented with semantic markup as source, implementing UK Phenome center naming conventions. Following validation, the workflow also allows visualization of overall study design and deposition to EMBL-EBI.	

#### Software tools

The Portal App Library [36] shows the software tools packaged in PhenoMeNal that are available through the CRE deployment (Fig. 2). The range of software tools available covers several metabolomics domains, making PhenoMeNal relevant for use in a wide range of data analysis scenarios. The domains covered include clinical metabolomics, plant metabolomics, fluxomics, and eco-metabolomics. Data from both targeted and untargeted analysis can be analyzed for metabolite profiling and fingerprinting approaches [1, 2]. NMR and MS (liquid chromatography coupled with mass spectrometry, gas chromatography coupled with mass spectrometry, direct infusion mass spectrometry) data can be processed.

PhenoMeNal also provides tools for data management (e.g., via the Investigation, Study, and Assay data model framework [ISA] format and application programming interface [API]), metabolite feature detection (e.g., XCMS, CAMERA, nmrProcFlow), metabolite identification (MetFrag, BATMAN, MetaboMatching), and (bio)statistics (e.g., univariate, multivariate, and power analyses) (Supplementary Table S1). Tools can be filtered for functionality, approaches, and instrument (data) types to readily find the most appropriate software tools. Some tools that implement specific functionality (e.g., Rnmr1D, which performs baseline correction of NMR spectra as part of nmrProcFlow) are available through dedicated Galaxy modules or through software containers (Supplementary Table S1).

#### Study design

PhenoMeNal was designed to use standardized protocols and software tools and to comply with state-of-the-art dedicated specifications and data formats across the entire project. Development was geared toward implementation of open standards for tracking provenance of both data and metadata generated by clinical phenotyping projects. In PhenoMeNal, the ISA model and specifications were implemented using the ISA format to generate, annotate, validate, and deposit experimental metadata information of datasets and studies to public repositories such as MetaboLights [37, 38]. ISA-based metadata tracking is used for the different analysis pipelines that are specific to the distinct metabolomics domains. PhenoMeNal reached native support for the ISA format by developing a dedicated Galaxy composite data type. Such component affords direct recognition of the ISA format by the Galaxy environment, thus ensuring seamless integration with the downstream workflow component.

#### Data deposition

PhenoMeNal encourages the metabolomics data repository MetaboLights as a primary source of data deposition [39]. Private and public datasets are supported, as are download and upload to MetaboLights. If the storage in a data repository such as MetaboLights is not possible, data can be stored locally or in the cloud e-infrastructure. Access to the data is strictly controlled and secured. To support data deposition, ISA-based Galaxy modules are available making it possible to publish and disseminate scientific results in standard compliant ways.

#### Reproducibility

One of the challenges of cloud computing is that analyses need to be run continuously and successfully in different environments [40]. Specifically, it has to be ensured that, given the same input, workflows and tools produce identical results regardless of the underlying environment [4, 40]. When these requirements are fulfilled, end users can be confident that their data will be analyzed correctly. PhenoMeNal has implemented three major testing strategies to ensure technical reproducibility using a continuous integration framework [41]. Tests were implemented for the infrastructure components, individual software containers, and data involved in computational workflows.

#### Sustainability

PhenoMeNal is part of a number of initiatives (BioMedBridges, COSMOS, and ELIXIR) to foster the role of metabolomics and to harmonize experimental data and metadata usage [15, 42]. Collaborations were established with EGI [43] and Indigo Datacloud [44] infrastructure providers and initiatives [45, 46] to ensure that PhenoMeNal uses technologies that are well supported and ensure their widespread usage, continuity, and further development. For example, the development of KubeNow and contributions to the Galaxy and Workflow4Metabolomics community are essential for PhenoMeNal [47]. Core development will continue on GitHub and is fostered by collaborations with tool developers.

Dependencies on specific technologies and frameworks were avoided by focusing on open standards such as ISA-Tab/ISA-JSON, mzML and nmrML, and widely accepted software [48]. By being able to deploy PhenoMeNal on multiple types of cloud environments, lock-ins to specific computing resource providers are avoided. PhenoMeNal implemented continuous integration and delivery, validated by extensive testing and with clear maintenance responsibilities (see Methods section).

#### Privacy and security

With human or animal material, the collection, storage, and analysis of metabolomics data introduce a number of constraints due to ethical, legal, and social implications (ELSI) [49]. In particular, data initially derived from human clinical studies may be identifiable and will require consent for use, usually for a defined objective, such as diagnosis, or be related to a particular disease study. Where data is identifiable or pseudonymized, users can deploy PhenoMeNal on local secure resources, thus avoiding the export of data. In this scenario, access to the e-infrastructure should be strictly controlled through local access and authorization. It is recommended that clinical data be fully anonymized before analysis in PhenoMeNal [49, 50].

The PhenoMeNal portal provides substantial guidance to enable users to comply with ELSI and general data protection regulation (GDPR) requirements. Users must register in order to use the individual parts of the e-infrastructure. PhenoMeNal was implemented to use secured and encrypted transport and network communications.

#### Documentation and training materials

Extensive user documentation and tutorials are provided via the PhenoMeNal Wiki page [51]. The Wiki includes detailed developer resources including information about the PhenoMeNal release schedule; guidelines for tool, workflow, and portal developers; continuous integration; and testing. Further documentation is also provided detailing, creating, and managing PhenoMeNal CREs and tutorials for the Galaxy modules and pre-configured workflows, as well as Galaxy tours that provide step-by-step guidance for inexperienced users.

#### Community engagement

The PhenoMeNal project is open source and is hosted on GitHub [52]. Developers can contribute tools to PhenoMeNal and are encouraged to do so. To add a tool to PhenoMeNal, it must be containerized using Docker and then integrated into the build process. Detailed documentation is available in the project's Wiki for developers who wish to add their tools to PhenoMeNal.

Collaborations with other projects have been actively encouraged during the development of PhenoMeNal, including Workflow4Metabolomics [13] and the developers of both nmrML and nmrProcFlow [26]. These collaborations are essential to fostering greater standardization within PhenoMeNal and to increasing compatibility with other metabolomics data processing infrastructures.

### Availability

Information on how to access PhenoMeNal can be found at the project's website [53]. The GitHub repository hosts the source code of all development projects [52]. The project container-galaxy-k8s-runtime contains all of the developments regarding Galaxy. The Wiki containing documentation is also hosted on GitHub [51]. The PhenoMeNal Portal can be reached at [21]. The public instance of Galaxy is accessible at [54]. Source code and documentation are available under the terms of the Apache 2.0 license. Integrated open-source projects are available under the respective licensing terms.

### Conclusions

PhenoMeNal has succeeded in increasing the robustness and coverage of representative metabolomics data processing in scientific cloud e-infrastructures. The presented cloud e-infrastructure covers a wide range of analysis pipelines including data generation and download, data pre- and post-processing, (bio)statistics, and result deposition in data repositories. A large effort has been made to introduce lower-level changes to cloud e-infrastructures (e.g., the cloud deployment software KubeNow) to meet the demands of the biomedical domain. Furthermore, Galaxy has been enriched with metabolomics data standards, in particular, the ISA format for study metadata and mzML and nmrML for acquired data files, as well as support for Kubernetes. PhenoMeNal has fostered the visibility of new metabolomics tools and has enabled the development of more sophisticated data analysis workflows. Our efforts were also guided by feedback from real-life test scenarios collected at workshops with users from the clinical domain.

PhenoMeNal constitutes a keystone solution in cloud platforms available for metabolomics data analysis. The platform was designed to deliver optimal performance and functionality for typical use cases in the metabolomics domain. While the needs of clinicians and researchers in the biomedical and biochemical domains have been targeted, PhenoMeNal is not limited to a specific domain as the cloud infrastructure, tools, and workflows can be adapted to other use cases as demonstrated with the inclusion of the eco-metabolomics workflow. The technological advancements can be reused in other scientific cloud environments and could be integrated with solutions from other ‘omics domains in the future.

## Methods

### Cloud e-infrastructure

The PhenoMeNal CRE is designed as a microservice architecture, with services being implemented as virtual machine images and software containers. Containers are used to provide microservices for metabolomics data analysis tools and also long-running services such as workflow management systems. A container orchestrator runs containers on top of the scalable infrastructure. The orchestrator takes a group of machines that act as a distributed cluster and receives requests for tools as well as service executions. PhenoMeNal implements various layers to providea container orchestrator on top of either bare metal hardware or IaaS given by a cloud provider [55] (Supplementary Fig. S1).

During the setup process and while PhenoMeNal is deployed, data storage and CPU limits can be configured and dynamically scaled to fit any cloud environment. Deployments can be made to GCE, AWS, and OpenStack-based private clouds from the PhenoMeNal portal. Deployments are also supported from the command line to Microsoft Azure [56], the European Science Cloud [57], and local servers (bare metal) [58]; we provide step-by-step instructions for these solutions.

PhenoMeNal provides IaaS for three different cloud environments:

“local cloud”: local workstations or bare metal clusters where data are not allowed to leave the facility.
“public cloud”: the flexible use of commercial cloud providers such as GCE and AWS.
“shared cloud”: using OpenStack—a free and open-source software platform for cloud computing, ideal for custom environments and research networks.

### Software tools

The PhenoMeNal portal has an application library that allows users to deploy tools as microservices into the cloud infrastructure (Fig. 2, Supplementary Table S1). The portal is packaged into frontend and backend engines on top of Kubernetes.

Most software tools in PhenoMeNal are compiled from source code and use a variety of programming languages. Linux versions of software tools and user interfaces such as Galaxy are supported in dedicated encapsulated Docker containers that are implemented as minimum-sized microservices. PhenoMeNal currently hosts 100 such projects in its GitHub repository [59] (Supplementary Table S1). Projects are indicated by the trailing `container-` name and include a ruleset to build and run the containerized tools, as well as datasets for testing and other necessary files.

PhenoMeNal provides tutorials for developers who want to integrate their tools into our e-infrastructure [60].

### Scientific workflows

In PhenoMeNal, a number of options are available for running reproducible and standardized workflows (Table 1).

#### Galaxy

The Galaxy workflow management system is widely regarded as one of the most popular scientific workflow platforms [20, 61]. It provides a user-friendly web-based GUI to make it easy for the end user to configure and run individual modules and entire workflows without programming experience. Command-line tools and scripts are encapsulated into modules that are launched via the web interface. Galaxy also supports more powerful features such as programmatic access through a REST API and helper libraries to access the running instance of Galaxy [62].

PhenoMeNal has been able to adapt Galaxy for use with a microservices-based architecture [31]. To this end, modules are encapsulated into Docker containers that can be flexibly launched within the cloud e-infrastructure. Galaxy is available in all deployed PhenoMeNal CREs and contains more than 250 modules that have been implemented as part of PhenoMeNal.

Six representative metabolomics Galaxy workflows have been fully integrated into PhenoMeNal (Table 1), and more workflows (mzQuality, NMR-BATMAN) are available for testing.

#### Jupyter

Jupyter, which started its history as the IPython notebook, is the most popular among the tools commonly referred to as executable notebooks or computational notebooks [63]. Jupyter lets users combine executable code with results from code executions such as text, tables, and figures. Usually, Jupyter notebooks are enriched with extended information that explains what the code does. As a result, they are often used for training material and for tutorials. Also, computational notebooks can, to some extent, be used as a way to document code executions and to make executions more reproducible [64].

#### Luigi and pachyderm

Luigi is a Python workflow programming library that was originally developed by the company Spotify. It manages pipelines of computations primarily on "big data" systems such as Hadoop and Apache Spark but also supports local execution [63, 64]. Luigi is a very flexible library that facilitates building complex pipelines of batch jobs handling dependency resolution, workflow management, and visualization.

Similarly, Pachyderm makes it possible to process distributed data and to keep track of the data from every stage of the analysis pipeline [25]. With Pachyderm, it is possible to track the provenance of results and to accurately reproduce scientific workflows. Luigi and Pachyderm are well suited for complex scientific tasks and are easy to use from the python environment in Jupyter notebooks without additional integration tooling needed.

In PhenoMeNal, we have extended Galaxy, Jupyter, Luigi, and Pachyderm in such a way that they can be orchestrated throughout the cloud infrastructure together with the data analysis tools themselves [31]. Six important metabolomics workflows have been fully integrated into PhenoMeNal (Table 1), and more (mzQuality, NMR-BATMAN) are available for testing.

### Reproducibility

Three strategies are realized to ensure technical reproducibility. They are implemented in the continuous integration (CI) software development framework Jenkins [41] which is accessible at [65]. These strategies are implemented as tests in our Jenkins and a tutorial guide is available at [66].

- Infrastructure testing: Procedures were implemented to ensure that each individual component (e.g., the deployment process of software containers, resource management, APIs/application binary interfaces [ABIs]) within the infrastructure is interacting correctly with the other components.
- Container testing: Verification that tools, which are packaged into software containers, build and run correctly in the infrastructure. Dependencies within one container and across several interdependent containers are tested.
- Data testing: The output of tools, which process demonstration data, is checked against a data set that is known to contain the expected result. This is being done for both individual tools and for several tools running in a workflow using the workflow testing tool for Galaxy called wft4galaxy [67].

### Standardization

PhenoMeNal has implemented several dedicated Galaxy modules that directly retrieve and store ISA-Tab data set descriptors from and to MetaboLights, and can convert between other formats. Native Galaxy composite data types to support ISA-Tab and ISA-JSON have also been integrated, building upon the ISA API [38, 48]. The ISA data type allows for the upload of an ISA-Tab archive (a zip file containing the ISA set of files and raw data when available), which is displayed to the users as a single Galaxy history data set. The integrated Galaxy modules include a MetaboLights downloader and uploader (for ingestion and submission), an ISAcreate module for the creation of ISA compliant archives, modules to explore study metadata through queries on study factors, ISA-Tab “slicing” where queries are used to select subsets of data files of interest, as well as format conversion (export to ISA-JSON and Workflow4Metabolomics [W4M]) and study metadata validation (Supplemental Table S1).

PhenoMeNal also advanced the specification of the nmrML standard data format [27] and contributed a dedicated composite data type for nmrML to Galaxy. nmrML is used extensively throughout the NMR 1D workflow and conversion from raw format into nmrML is supported via dedicated Galaxy modules (Table 1).

Throughout the entire analysis pipeline, modules of computational workflows were designed to accept standard formats such as mzML, XML or CSV whenever possible.

Standardized APIs/ABIs are being used for the programmatic interfaces as well as for deploying services. To this end, modern and standardized programming, scripting and meta languages were selected such as Go, HCL, Python, Shell, XML and YAML that are widely used in cloud computing.

### Reusability

In an ongoing effort, PhenoMeNal is actively advancing the criteria for good data management and stewardship based on findability, accessibility, interoperability and reusability (FAIR) for good data management and stewardship [68] to be applied not only to data, but also to software tools and computational workflows (Table 2).

**Table 2:** Overview of the most important FAIR criteria and implementations suggested for PhenoMeNal data, tools and workflows

	Data	Tools	Workflows
**(F)indability**	Indexing in domain relevant databases (e.g., MetaboLights)	Indexing in domain relevant software repositories (e.g., the PhenoMeNal App Library, GitHub)	Indexing in workflow management systems such as Galaxy (e.g., PhenoMeNal, W4M), or libraries such as [69]
	Rich descriptions of metadata (e.g., ISA-Tab)	Tool descriptions follow the EDAM ontology	Persistent identifier (e.g., W4M ID, DOI) and intuitive naming patterns
**(A)ccessibility**	Data access and rights management based on e.g., data use ontology (DUO)	Accessible open-source licenses	Access to workflow systems can be configured to be shared or restricted
**(I)nteroperability**	Standard formats for experimental metadata (ISA-Tab/ISA-JSON)	Standardized tool descriptions	Standardized workflow format (e.g., Galaxy GA format, Common Workflow Language CWL)
	Domain specific standards for raw data (e.g., mzML, nmrML)	Containerization of software tools	Execution in various software environments (e.g., through the use of containers)
	OboFoundry vocabularies and established domain ontologies to annotate data	EDAM ontology to annotate tools	Workflow annotation ontologies (e.g., Ontology of workflow motifs for annotating workflow specifications [70])
**(R)eusability**	Deposition in data repositories (e.g., MetaboLights) and data indexing sites (e.g., OmicsDI)	Rich documentation and usage guides	Rich documentation and tutorials (e.g., Galaxy tours)

### Privacy

PhenoMeNal supports fully anonymized data, which cannot be traced back to individuals in any way [50] and treats pseudonymized data as identifiable. As pseudonymized data are anonymous to the investigator, third parties may be able to link pseudonymized data back to identifiable individuals through mappings such as a hash or code [49]. In these cases, e.g., in a hospital environment, users must deploy PhenoMeNal within a private cloud or bare metal cluster behind their institution's firewall.

PhenoMeNal provides guidance on ethical and technical frameworks to regulate and secure the use of private or sensitive data [49, 50]. It is possible to combine data and metadata within an ELSI compliant framework [50] and in such cases users can follow the example of the European Genome Phenome Archive (EGA) [71]. In public installations of PhenoMeNal, the ELIXIR policy on privacy has been implemented within a technically secure environment to process data [42].

### Security

Open-source tools are used throughout the entire e-infrastructure. This promotes community efforts to discover and resolve bugs and security issues. The container build process is steered by the continuous integration (CI) service Jenkins, which continuously builds the containers and generates reports. On success and through authentication, container images are pushed to the PhenoMeNal container registry, which is publicly available but read-only. Cloud provider credentials are not stored in the cloud but only on the deployer host. The Kubernetes cluster running the Jenkins-CI and the container registry, as well as the portal, runs on a CoreOS container, which is a self-updatable, cluster-aware system with most portions being read-only. It reboots nodes sequentially to avoid lack of availability.

KubeNow is a key component that initializes the cloud infrastructure and configures access to it via Cloudflare [72], providing dynamic Domain Name Services (DNS) and encryption for all network communication. The flexible implementation of PhenoMeNal allows the user to decide to not use Cloudflare, in which case encryption is disabled. KubeAdm, which manages the setup of Kubernetes, is not reachable at runtime by default. The only way to access it is by having access to the private key stored on the computer on which it was launched. PhenoMeNal only allows access to standard ports (ssh, http, https, and port 44 for the Galaxy Downloader) and implements a cloud-specific firewall for all supported cloud providers.

Microservices are designed to be launched on-demand and terminated after completed analysis. If security issues are reported for the microservices, tool, or dependencies or if incremental security patches are available, new builds are automatically triggered in the CI system and developers and the release manager are notified to take additional actions if required. Images are built on a daily basis and tested for deployment to avoid security patches from introducing any abnormality in the deployment process.

### User resources

There are many user resources for both PhenoMeNal users and developers in the form of documentation, tutorials, and training videos. The PhenoMeNal Wiki [51] contains detailed documentation on all aspects of PhenoMeNal, including general user guides, workflow and tool tutorials, developer documentation, and general information on topics such as security and the e-infrastructure landscape. The PhenoMeNal portal contains help pages generated from the Wiki [73], which are categorized as User Documentation, Developer Documentation, and Workflow Tutorials. Interactive Galaxy tours are directly integrated in Galaxy [74]. Training videos are available at the project's YouTube page [75].

## Supplementary Material

GIGA-D-18-00347_Orginal_Submission.pdf

GIGA-D-18-00347_Revision_1.pdf

Response_to_Reviewer_Comments_Original_Submission.pdf

Reviewer_1_Report_Original_Submission -- H Paul Benton 9/24/2018 Reviewed

Reviewer_2_Report_(Original_Submission) -- Saravanan Dayalan 9/25/2018 Reviewed

Supplemental Files

## Data Availability

The following MetaboLights datasets are integrated into PhenoMeNal and are used to demonstrate the cloud integration and reproducibility of Galaxy workflows: MTBLS1 (NMR1D), MTBLS404 (Uni- and multivariate statistics), MTBLS412 (Fluxomics), MTBLS520 (Eco-Metabolomics), MTBLS558 (MetFrag). Datasets are available at https://www.ebi.ac.uk/metabolights. Snapshots of the code and additional supporting data are available in the *GigaScience* repository, GigaDB [76].

## Availability of source code and requirements

Project name: PhenoMeNal,

Project home page: http://phenomenal-h2020.eu

Operating system(s): Platform independent

Programming language: Go, HCL, Java, JavaScript, Python, R, Shell, XML, YAML

Other requirements: Linux, Docker, Kubernetes, Terraform, Ansible, Helm

License: MIT license for all code written by the PhenoMeNal project. Individual, Open Source Foundation approved licenses for all containerized tools.

RRID:SCR_016605

## Additional files

**Supplemental Figure 1:** PhenoMeNal implements various layers to provision containers on top of the e-infrastructure.

**Supplemental Table 1:** List of external software tools that were incorporated into PhenoMeNal.

## Abbreviations

ABI: application binary interface; API: application programming interface; AWS: Amazon web services; CI: continuous integration; CPU: central processing unit; CRE: cloud research environment; ELSI: ethical, legal, and social implications; FAIR: criteria for good data management and stewardship based on findability, accessibility, interoperability, and reusability; GCP: Google cloud platform; GUI: graphical user interface; IaaS: Infrastructure-as-a-Service; ISA: Investigation, Study, and Assay data model framework; MS: mass spectrometry; NMR: nuclear magnetic resonance (spectroscopy); PhenoMeNal: Phenome and Metabolome aNalysis; W4M: Workflow4Metabolomics.

## Declarations

Human-derived samples in the datasets MTBLS404 and MTBLS412 were processed according to ELSI guidelines.
