# Supplementary material for: PhenoMeNal: processing and analysis of metabolomics data in the cloud
Source: Gigascience. 2018 Dec 7;8(2):giy149. doi: 10.1093/gigascience/giy149 (PMC6377398; doi:10.1093/gigascience/giy149)
Supplement: GIGA-D-18-00347_Orginal_Submission.pdf [file giy149_giga-d-18-00347_orginal_submission.pdf]

GigaScience

PhenoMeNal: Processing and analysis of Metabolomics data in the Cloud

--Manuscript Draft--

**Manuscript Number:**	GIGA-D-18-00347
**Full Title:**	PhenoMeNal: Processing and analysis of Metabolomics data in the Cloud
**Article Type:**	Technical Note
**Funding Information:**	H2020 European Research Council (EC654241) — Not applicable
**Abstract:**	Background: Metabolomics is the comprehensive study of a multitude of small molecules to gain insight into an organism's metabolism. The research field is dynamic and expanding with applications across biomedical, biotechnological and many other applied biological domains. Its computationally-intensive nature has driven requirements for open data formats, data repositories and data analysis tools. However, the rapid progress has resulted in a mosaic of independent, and sometimes incompatible, analysis methods that are difficult to connect into a useful and complete data analysis solution.<br>Findings: The PhenoMeNal (Phenome and Metabolome aNalysis) e-infrastructure provides a complete, workflow-oriented, interoperable metabolomics data analysis solution for a modern infrastructure-as-a-service (IaaS) cloud platform. PhenoMeNal seamlessly integrates a wide array of existing open source tools which are tested and packaged as Docker containers through the project's continuous integration process and deployed based on a kubernetes orchestration framework. It also provides a number of standardized, automated and published analysis workflows in the user interfaces Galaxy, Jupyter, Luigi and Pachyderm.<br>Conclusions: PhenoMeNal constitutes a keystone solution in cloud infrastructures available for metabolomics. It provides scientists with a ready-to-use, workflow-driven, reproducible and shareable data analysis platform harmonizing the software installation and configuration through user-friendly web interfaces. The deployed cloud environments can be dynamically scaled to enable large-scale analyses which are interfaced through standard data formats, versioned, and have been tested for reproducibility and interoperability. The flexible implementation of PhenoMeNal allows easy adaptation of the infrastructure to other application areas and 'omics research domains.
**Corresponding Author:**	Christoph Steinbeck, Dr. rer. net.<br>Friedrich-Schiller-Universität Jena<br>Prof. Dr., Thüringen GERMANY
**Corresponding Author Secondary Information:**	
**Corresponding Author's Institution:**	Friedrich-Schiller-Universität Jena
**Corresponding Author's Secondary Institution:**	
**First Author:**	Kristian Peters
**First Author Secondary Information:**	
**Order of Authors:**	Kristian Peters
	James Bradbury
	Sven Bergmann
	Marco Capuccini
	Marta Cascante
	Pedro de Atauri
	Tim Ebbels
	Carles Foguet
	Robert C Glen

Powered by Editorial Manager® and ProduXion Manager® from Aries Systems Corporation

	Alejandra Gonzalez-Beltran
	Evangelos Handakas
	Thomas Hankemeier
	Stephanie Herman
	Kenneth Haug
	Petr Holub
	Massimiliano Izzo
	Daniel Jacob
	David Johnson
	Fabien Jourdan
	Namrata Kale
	Ibrahim Karaman
	Bita Khalili
	Payam Emami Khoonsari
	Kim Kultima
	Samuel Lampa
	Anders Larsson
	Pablo Moreno
	Steffen Neumann
	Jon Ander Novella
	Claire O'Donovan
	Jake TM Pearce
	Alina Peluso
	Luca Pireddu
	Marco Enrico Piras
	Michelle AC Reed
	Philippe Rocca-Serra
	Pierrick Roger
	Antonio Rosato
	Rico Rueedi
	Christoph Ruttkies
	Noureddin Sadawi
	Reza Salek
	Susanna-Assunta Sansone
	Vitaly Selivanov
	Ola Spjuth
	Daniel Schober
	Etienne A. Thévenot
	Mattia Tomasoni
	Merlijn van Rijswijk
	Michael van Vliet

Powered by Editorial Manager® and ProduXion Manager® from Aries Systems Corporation

	Mark Viant
	Ralf Weber
	Gianluigi Zanetti
	Christoph Steinbeck, Dr. rer. net.

**Order of Authors Secondary Information:**	
**Additional Information:**	
**Question**	**Response**
Are you submitting this manuscript to a special series or article collection?	No
**Experimental design and statistics**<br><br>Full details of the experimental design and statistical methods used should be given in the Methods section, as detailed in our Minimum Standards Reporting Checklist. Information essential to interpreting the data presented should be made available in the figure legends.<br><br>Have you included all the information requested in your manuscript?	Yes
**Resources**<br><br>A description of all resources used, including antibodies, cell lines, animals and software tools, with enough information to allow them to be uniquely identified, should be included in the Methods section. Authors are strongly encouraged to cite Research Resource Identifiers (RRIDs) for antibodies, model organisms and tools, where possible.<br><br>Have you included the information requested as detailed in our Minimum Standards Reporting Checklist?	Yes
**Availability of data and materials**<br><br>All datasets and code on which the conclusions of the paper rely must be either included in your submission or	Yes

deposited in publicly available repositories (where available and ethically appropriate), referencing such data using a unique identifier in the references and in the "Availability of Data and Materials" section of your manuscript.<br><br>Have you have met the above requirement as detailed in our Minimum Standards Reporting Checklist?	

Manuscript
Click here to access/download;Manuscript;msfile-1.docx
Click here to view linked References

PhenoMeNal: Processing and analysis of Metabolomics data in the Cloud

Kristian Peters*[,1], James Bradbury*[,2], Sven Bergmann[3,4], Marco Capuccini[5,6], Marta Cascante[7], Pedro de Atauri[8], Timothy M D Ebbels[9], Carles Foguet[8], Robert Glen[9,10], Alejandra Gonzalez-Beltran[11], Evangelos Handakas[9], Thomas Hankemeier[12,13], Kenneth Haug[14], Stephanie Herman[15], Massimiliano Izzo[11] , Daniel Jacob[16], David Johnson[11], Fabien Jourdan[17], Namrata Kale[14], Petr Holub[29], Ibrahim Karaman[18], Bita Khalili[3,4], Payam Emami Khonsari[15], Kim Kultima[15], Samuel Lampa[6], Anders Larsson[19], Pablo Moreno[14], Steffen Neumann[1,20], Jon Ander Novella[19], Claire O'Donovan[14], Jake TM Pearce[9], Alina Peluso[9], Luca Pireddu[21], Marco Enrico Piras[21], Michelle AC Reed[22], Philippe Rocca-Serra[11], Pierrick Roger[23], Antonio Rosato[24], Rico Rueedi[3,4], Christoph Ruttkies[1], Noureddin Sadawi[9], Reza M Salek[25], Susanna-Assunta Sansone[11], Vitaly Selivanov[8], Ola Spjuth[6], Daniel Schober[1], Etienne A. Thévenot[23], Mattia Tomasoni[3,4], Merlijn van Rijswijk[26,27], Michael van Vliet[28], Mark R Viant[2], Ralf J. M. Weber[2], Gianluigi Zanetti[21], , Christoph Steinbeck*[,30]

* - corresponding authors

[1] - Leibniz Institute of Plant Biochemistry, Stress and Developmental Biology, Weinberg 3, 06120 Halle (Saale), Germany

[2] - School of Biosciences, University of Birmingham, Edgbaston, Birmingham, B15 2TT, United Kingdom

[3] - Department of Computational Biology, University of Lausanne, Lausanne, Switzerland

[4] - Swiss Institute of Bioinformatics, Lausanne, Switzerland

[5] - Division of Scientific Computing, Department of Information Technology, Uppsala University, Sweden

[6] - Department of Pharmaceutical Biosciences, Uppsala University, Box 591, 751 24 Uppsala, Sweden

[7] - Department of Biochemistry and Molecular Biomedicine, Universitat de Barcelona; Centro de Investigación Biomédica en Red de Enfermedades Hepáticas y Digestivas

[8] - Department of Biochemistry and Molecular Biomedicine, Universitat de Barcelona; Centro de Investigación Biomédica en Red de Enfermedades Hepáticas y Digestivas (CIBEREHD), Instituto de Salud Carlos III (ISCIII), Spain

[9] - Department of Surgery & Cancer, Imperial College London, South Kensington, London, SW7 2AZ, United Kingdom

[10] - Centre for Molecular Informatics, Department of Chemistry, University of Cambridge, Lensfield Road, Cambridge, CB21EW, United Kingdom

[11] - Oxford e-Research Centre, Department of Engineering Science, University of Oxford, 7 Keble Road, OX1 3QG, Oxford, UK.

[12] - Netherlands Metabolomics Center, Leiden, 2333 CC, Netherlands

[13] - Division of Systems Biomedicine and Pharmacology, Leiden Academic Centre for Drug

[14] - European Molecular Biology Laboratory, European Bioinformatics Institute (EMBL-EBI), Wellcome Genome Campus, Hinxton, Cambridge CB10 1SD, UK

[15] - Department of Medical Sciences, Clinical Chemistry, Uppsala University, 751 85 Uppsala, Sweden

[16] - INRA, University of Bordeaux, Plateforme Métabolome Bordeaux-MetaboHUB, 33140 Villenave d'Ornon, France

[17] - INRA - French National Institute for Agricultural Research, UMR1331, Toxalim, Research Centre in Food Toxicology, Toulouse, France
[18] - Department of Epidemiology and Biostatistics, School of Public Health, Imperial College London, St. Mary's Campus, Norfolk Place, W2 1PG, London, United Kingdom
[19] - National Bioinformatics Infrastructure Sweden, Uppsala University, Uppsala, Sweden
Department of Pharmaceutical Biosciences, Uppsala University, Uppsala, Sweden
[20] - German Centre for Integrative Biodiversity Research (iDiv) Halle-Jena-Leipzig, Deutscher Platz 5e, 04103 Leipzig, Germany
[21] - Distributed Computing Group, CRS4, Pula, Italy
[22] - College of Medical and Dental Sciences, University of Birmingham, Edgbaston, Birmingham, B15 2TT, United Kingdom
[23] - CEA, LIST, Laboratory for Data Analysis and Systems' Intelligence, MetaboHUB, Gif-Sur-Yvette F-91191, France
[24] - Magnetic Resonance Center (CERM) and Department of Chemistry, University of Florence and CIRMMP, 50019 Sesto Fiorentino, Florence, Italy
[25] - European Bioinformatics Institute (EMBL-EBI), European Molecular Biology Laboratory, Wellcome Genome Campus, Hinxton, Cambridge CB10 1SD, U.K.
[26] - ELIXIR-NL, Dutch Techcentre for Life Sciences, Utrecht, 3503 RM, Netherlands
[27] - Netherlands Metabolomics Center, Leiden, 2333 CC, The Netherlands
[28] - Division of Systems Biomedicine and Pharmacology, Leiden Academic Centre for Drug Research (LACDR), Leiden University, Leiden, 2333 CC, The Netherlands
[29] - BBMRI-ERIC, Graz, Austria
[30] - Cheminformatics and Computational Metabolomics, Institute for Analytical Chemistry, Lessingstr. 8, 07743 Jena, Germany

Abstract

Background: Metabolomics is the comprehensive study of a multitude of small molecules to gain insight into an organism's metabolism. The research field is dynamic and expanding with applications across biomedical, biotechnological and many other applied biological domains. Its computationally-intensive nature has driven requirements for open data formats, data repositories and data analysis tools. However, the rapid progress has resulted in a mosaic of independent – and sometimes incompatible – analysis methods that are difficult to connect into a useful and complete data analysis solution.
Findings: The PhenoMeNal (Phenome and Metabolome aNalysis) e-infrastructure provides a complete, workflow-oriented, interoperable metabolomics data analysis solution for a modern infrastructure-as-a-service (IaaS) cloud platform. PhenoMeNal seamlessly integrates a wide array of existing open source tools which are tested and packaged as Docker containers through the project's continuous integration process and deployed based on a kubernetes orchestration framework. It also provides a number of standardized, automated and published analysis workflows in the user interfaces Galaxy, Jupyter, Luigi and Pachyderm.
Conclusions: PhenoMeNal constitutes a keystone solution in cloud infrastructures available for metabolomics. It provides scientists with a ready-to-use, workflow-driven, reproducible and shareable data analysis platform harmonizing the software installation and configuration through user-friendly web interfaces. The deployed cloud environments can be dynamically scaled to enable large-scale analyses which are interfaced through standard data formats, versioned, and have been tested for reproducibility and interoperability. The flexible implementation of PhenoMeNal allows easy adaptation of the infrastructure to other application areas and 'omics research domains.

Keywords

Metabolomics, Data Analysis, e-infrastructures, NMR, Mass Spectrometry, Computational Workflows, Galaxy, Cloud Computing, Standardization, Statistics

Findings

Background

The field of metabolomics has seen remarkable progress over the last decade and has enabled fascinating discoveries in many different research areas. Metabolomics is the study of small molecules in organisms which can reveal detailed insights into metabolic biochemistry, e.g. changes in concentrations of specific molecules, metabolic fluxes between cells or compartments, identification of molecules that are involved in the pathogenesis of a disease, the study of the biochemical phenotype of animals, plants and even soil microorganisms [1–3]

The principal metabolomics technologies of mass spectrometry (MS) and nuclear magnetic resonance spectroscopy (NMR) typically generate large data sets that require computationally intensive analyses [4]. For example, biomedical investigations can involve large cohorts with many thousands of metabolite profiles and can produce terabytes of data [5]. With such large data sets, processing becomes impracticable and unmanageable on commodity hardware. Cloud computing can offer a solution by enabling the outsourcing of calculations from local workstations to scalable cloud data centers, with the possibility to allocate thousands of CPU cores simultaneously. Furthermore, cloud computing allows for resources to be instantiated on-demand (CPUs, RAM, network, storage) and access to computational tools in the form of microservices that can dynamically grow or shrink.

MS and NMR data processing usually involves selection of parameters (which are often specific to the analytical instrumentation), algorithmic peak detection, peak alignment and grouping, annotation of putative compounds and extensive statistical analyses [6,7]. Many open source tools have been developed that address these different steps in data processing and analysis. These tools, however, usually come with their own software dependencies, resource requirements and scripting languages. As a consequence, configuring and running them is often complicated, especially for researchers who are untrained in computer science [4]. Furthermore, many tools require users to input parameters that can significantly affect results and performance, and reporting of these parameters is not always clear [8].

In the last five years, a number of infrastructures and integration efforts were initiated, including metabolomics data repositories with a global scope [9,10], platforms for reproducible workflow analysis [11,12], as well as initiatives to integrate and coordinate data standards [13]. Simultaneously, multiple networks of service centers such as the international Phenome Centers and MetaboHub (http://phenomenetwork.org, http://www.metabohub.fr/metabohub.html) have formed with the goal to facilitate the acquisition, processing and analysis of metabolomics data [9,14,15] at ever increasing scales.

Here we present PhenoMeNal, a robust and performant data analysis e-infrastructure that provides a large suite of standardized and interoperable metabolomics data processing tools as a complete data analysis solution. The PhenoMeNal e-infrastructure can be easily deployed onto public and private cloud environments, enabling scalable and cost-effective

high-performance metabolomics data analysis in a way that hides the technical complexity from the user. PhenoMeNal facilitates reproducible analyses through automated, sharable and citable workflows.

Overview

The features of the PhenoMeNal e-infrastructure are encapsulated as a Cloud Research Environment (CRE). The PhenoMeNal CRE can be instantiated on major commercial public cloud providers, including Amazon Web Services (AWS) and Google Cloud Platform (GCE), as well as OpenStack-based private clouds. Technical complexity is hidden from the users, simplifying setting up the cloud infrastructure for administrators. From a web-based portal, users can deploy the CRE, which includes several web services and software tools. Data can be processed directly in the e-infrastructure without the need to install additional software. Scientific workflows can be executed via user friendly web-based platforms such as Galaxy, as well as programmatic interfaces and notebooks. Each service has been supplied with a rich source of documentation and training material to assist researchers.

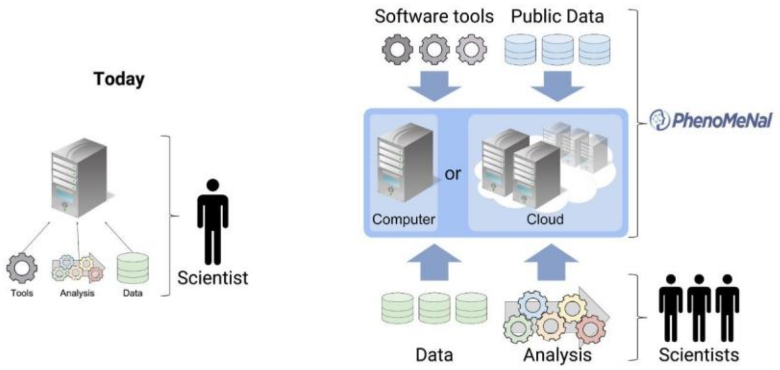

Fig. 1: Conceptual design of the PhenoMeNal cloud e-infrastructure (right hand side) compared to traditional approaches in biomedicine (left side).

The PhenoMeNal Portal

The PhenoMeNal portal (https://portal.phenomenal-h2020.eu/) allows users to deploy, manage and delete PhenoMeNal CREs simply through a web interface. Deployments to the three major commercial cloud platforms (AWS and GCP) as well as OpenStack, an open source cloud platform, can be made using an easy to follow wizard (Fig. 2). OpenStack deployments can be deployed behind clinical firewalls, which is especially pertinent when dealing with sensitive (i.e. patient) data.

The PhenoMeNal public instance allows users to test-run a CRE without the need to deploy on a cloud platform. It can be deployed and accessed through the portal (Fig. 2). Once credentials for users have been generated, analyses can be run through a Galaxy instance containing the tools and workflows present in any deployed CRE. The portal also includes user and developer documentation, workflow tutorials and links to training videos.

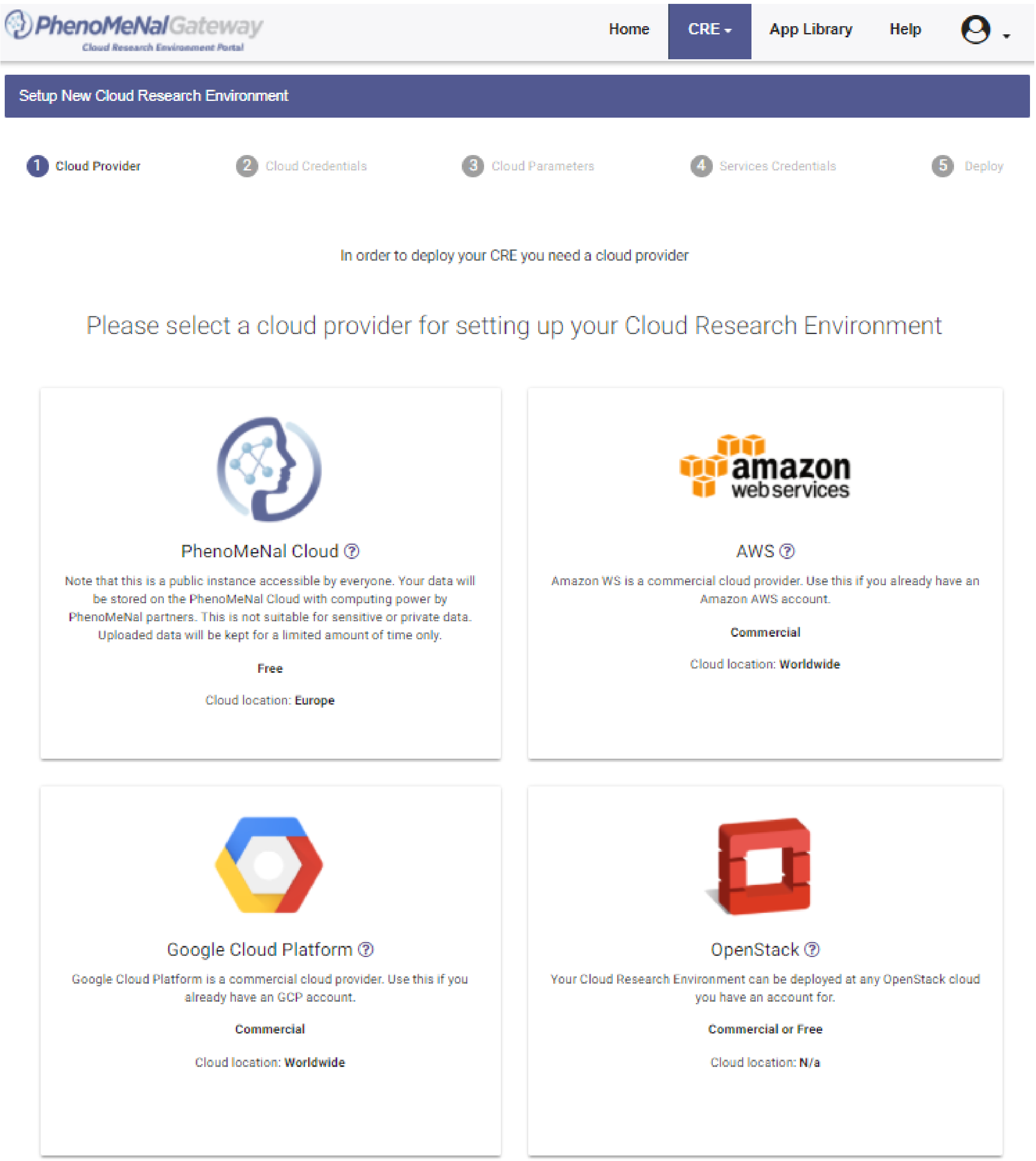

Fig. 2: Screenshot of selecting a cloud provider during setup of the Cloud Research Environment (CRE) using the dedicated PhenoMeNal portal.

Scientific workflows

A scientific workflow is a set of computational steps that are carried out to process and analyze data [16]. Usually, a workflow is comprised of several linked software tools that are each executed during a particular step of the workflow. In order to manage and automate scientific workflows, PhenoMeNal uses the well-established dedicated workflow management system Galaxy, which presents the user with an easy to use graphical user interface as well as providing a programmatic interface [17,18]. Galaxy facilitates collaborative exchange, reproducibility and traceability of data analysis by enabling users to share entire workflows and analysis histories [19]. In addition to Galaxy, programmatic

executable notebooks (Jupyter) and the programmatic interfaces Luigi and Pachyderm are also supported [20].

In order to cover typical use cases in metabolomics and to illustrate the usage and applicability of given analytical pipelines and software tools, five representative scientific workflows are available in the PhenoMeNal CRE Galaxy environment (Table 2, Fig. 6), each having different computational demands and purposes. More than 250 individual modules have been integrated in Galaxy (see section scientific workflows in Methods).

Software tools

The Portal App Library (https://portal.phenomenal-h2020.eu/app-library) shows all the software tools packaged in PhenoMeNal that are available through the CRE deployment (Fig. 3). The range of software tools available cover several metabolomics domains, making PhenoMeNal relevant for use in a wide range of data analysis scenarios. The domains covered include clinical metabolomics, plant metabolomics, fluxomics and eco-metabolomics. Data from both targeted and untargeted analysis can be analyzed for metabolite profiling and fingerprinting approaches [1,2]. NMR and MS (LC/MS, GC/MS, DIMS) data can be processed.

PhenoMeNal also provides tools for data management (e.g. via the ISA format and API), metabolite feature detection (e.g. XCMS, CAMERA, nmrProcFlow), metabolite identification (MetFrag, BATMAN, MetaboMatching) and (bio)statistics (e.g. univariate, multivariate and power analyses) (Table 1). Tools can be filtered for functionality, approaches and instrument (data) types to readily find the most appropriate software tools (Fig. 3). Some tools that implement specific functionality (e.g. Rnmr1D which performs baseline correction of NMR spectra as part of nmrProcFlow) are available through dedicated Galaxy modules or through software containers (Table 1).

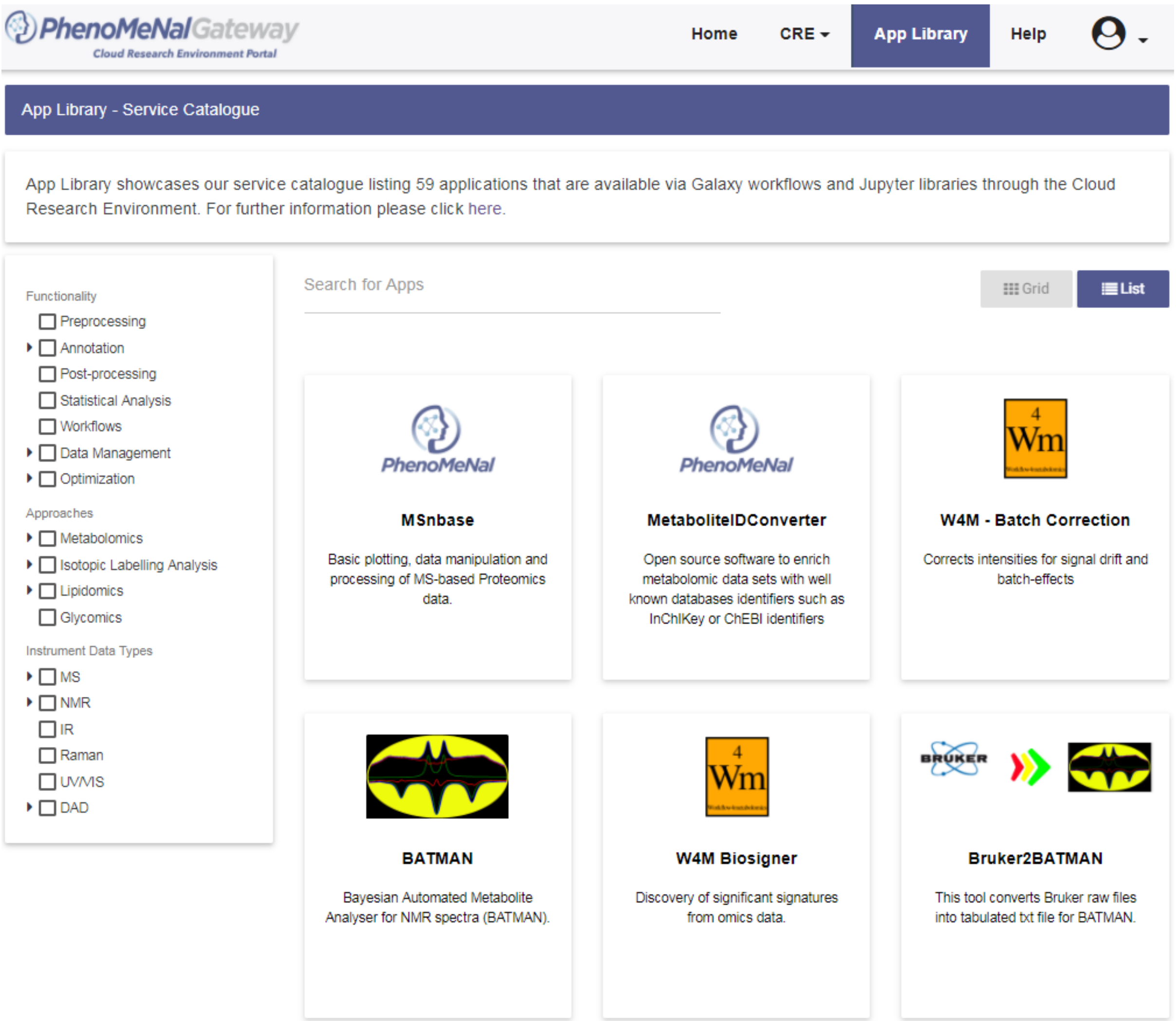

Fig. 3: Screenshot of the PhenoMeNal Portal App library.

Study Design

PhenoMeNal was designed to use standardized protocols, software tools and comply with state-of-the-art dedicated specifications and data formats across the entire project. Development was geared towards implementation of open standards for tracking provenance of both data and metadata generated by clinical phenotyping projects. In PhenoMeNal, the ISA model and specifications were implemented using the ISA format to generate, annotate, validate and deposit experimental metadata information of data sets and studies to public repositories such as MetaboLights [21,22]. ISA based metadata tracking is used for the different analysis pipelines which are specific to the distinct metabolomics domains. PhenoMeNal reached native support for the ISA format by developing a dedicated Galaxy composite data type. Such component affords direct recognition of the ISA format by the Galaxy environment, thus ensuring seamless integration with downstream workflow component.

Data deposition

PhenoMeNal encourages the metabolomics data repository MetaboLights as a primary source of data deposition [23]. Private and public data sets are supported, as well as

download and upload to MetaboLights. If the storage in a data repository such as MetaboLights is not possible, data can be stored locally or in the cloud e-infrastructure. Access to the data is strictly controlled and secured. To support data deposition, ISA based Galaxy modules are available allowing to publish and disseminate scientific results in standard compliant ways (see also Table 2).

Reproducibility

One of the challenges of cloud computing is that analyses need to be run continuously and successfully in different environments [24]. Specifically, it has to be ensured that, given the same input, workflows and tools produce identical results regardless of the underlying environment [4,24]. When these requirements are fulfilled, end users can be confident that their data will be analyzed correctly. PhenoMeNal has implemented three major testing strategies to ensure technical reproducibility using a continuous integration framework [25]. Tests were implemented for the infrastructure components, individual software containers and for data involved in computational workflows.

Sustainability

PhenoMeNal is part of a number of initiatives (BioMedBridges, COSMOS and ELIXIR) to foster the role of metabolomics and to harmonize experimental data and metadata usage [13,26]. Collaborations were established with EGI and Indigo Datacloud, to ensure that PhenoMeNal uses technologies that are well-supported and assure their widespread usage, continuity and further development. For example, the development of KubeNow and contributions to the Galaxy and Workflow4Metabolomics community are essential for PhenoMeNal. Core development will continue on GitHub and is fostered by collaborations with tool developers.

Dependencies on specific technologies and frameworks were avoided by focusing on open standards such as ISA-Tab / ISA-JSON, mzML and nmrML and widely accepted software [27]. By being able to deploy PhenoMeNal on multiple types of cloud environments, lock-in to specific computing resource providers are avoided. PhenoMeNal implemented continuous integration and delivery, validated by extensive testing and with clear maintenance responsibilities.

Privacy and security

With human or animal material the collection, storage and analysis of metabolomics data introduce a number of constraints due to Ethical, Legal and Social Implications (ELSI) [28]. In particular, data initially derived from clinical studies may be identifiable and will require consent for use, usually for a defined objective such as diagnosis, or be related to a particular disease study. PhenoMeNal has support for fully anonymized and pseudonymized data (where identifiers are removed, but data can be identified through a mapping such as a hash or code) [29]. In general, PhenoMeNal implements and is fully compliant to the ELSI guidelines [28]. PhenoMeNal has been designed to accommodate cases where sensitive data needs to be processed according to the General Data Protection Regulation (GDPR).

With sensitive or private data, users can always deploy PhenoMeNal on local resources only, thus avoiding the export of data altogether. Access to the e-infrastructure is strictly

controlled through authorization. Users must register in order to use the individual parts of the e-infrastructure. Transport and network communications are secured and encrypted. PhenoMeNal is part of the NeIC-Tryggve2 (https://neic.no/tryggve2/) project to further steer the development of secure data storage of biomedical research data.

Documentation and Training materials

Extensive user documentation and tutorials are provided via the PhenoMeNal Wiki page (https://github.com/phnmnl/phenomenal-h2020/wiki). The Wiki includes detailed developer resources including information about the PhenoMeNal release schedule, guidelines for tool, workflow and portal developers, continuous integration and testing. Further documentation detailing, creating and managing PhenoMeNal CREs, tutorials for the Galaxy modules and pre-configured workflows, as well as Galaxy tours that provide step by step guidance for inexperienced users are also provided.

Community engagement

The PhenoMeNal project is open source, and is hosted on GitHub (https://github.com/phnmnl/). Developers can contribute tools to PhenoMeNal and are encouraged to do so. To add a tool to PhenoMeNal, it must be containerized using Docker, and then integrated into the build process. Detailed documentation is available in the project's Wiki for developers who wish to add their tools to the PhenoMeNal CRE.

Collaborations with other projects have been actively encouraged during the development of PhenoMeNal, including Workflow4Metabolomics [11] and the developers of both nmrML and nmrProcFlow [30]. These collaborations are essential to foster greater standardization within PhenoMeNal and to increase compatibility with other metabolomics data processing infrastructures.

Availability

Information on how to access PhenoMeNal can be found at https://phenomenal-h2020.eu. The GitHub repository https://github.com/phnmnl/ hosts the source code of all development projects. The project container-galaxy-k8s-runtime contains all of the developments regarding Galaxy. The Wiki containing documentation is also hosted on GitHub https://github.com/phnmnl/phenomenal-h2020/wiki. The PhenoMeNal Portal can be reached at https://portal.phenomenal-h2020.eu. The public instance of Galaxy is accessible at https://public.phenomenal-h2020.eu. Source code and documentation are available under the terms of the Apache 2.0 license. Integrated open source projects are available under the respective licensing terms.

Conclusions

PhenoMeNal has succeeded in increasing the robustness and coverage of representative metabolomics data sets and workflows. Our efforts were also guided by feedback from real life test scenarios collected at workshops with users from the clinical domain. The e-infrastructure covers a wide range of analysis pipelines including data generation and download, data pre- and post-processing, (bio)statistics and result deposition in appropriate

data repositories. PhenoMeNal has fostered the visibility of new metabolomics tools and has enabled the development of more sophisticated data analysis workflows. A large effort has been made to introduce lower level changes to cloud e-infrastructures (e.g. the cloud deployment software KubeNow) to meet the demands of the biomedical domain. Furthermore, Galaxy has been enriched with metabolomics data standards, in particular the ISA format for study metadata and mzML and nmrML for acquired data files, as well as support for Kubernetes.

PhenoMeNal constitutes a keystone solution in cloud platforms available for metabolomics data analysis. The platform was designed to deliver optimal performance and functionality for typical use cases in the metabolomics domain. While the needs of clinicians and researchers in the biomedical and biochemical domains have been targeted, PhenoMeNal is not limited to a specific domain as the cloud infrastructure, tools and workflows can be adapted to other use cases as demonstrated with the inclusion of the eco-metabolomics workflow. The technological advancements can be reused in other scientific cloud environments and could be integrated with solutions from other ‘omics domains in the future.

Methods

Cloud e-infrastructure

The PhenoMeNal CRE is designed as a microservice architecture, with services being implemented as Virtual Machine Images (VMIs) and software containers. Containers are used to provision microservices for metabolomics data analysis tools and also long-running services such as workflow management systems. A container orchestrator runs containers on top of the scalable infrastructure. The orchestrator takes a group of machines that act as a distributed cluster and receives requests for tools as well as services executions. PhenoMeNal implements various layers to provision a container orchestrator on top of either bare metal hardware or Infrastructure as a Service (IaaS) given by a cloud provider [31] (Fig. 4).

Starting from the IaaS, the first layer is a cluster of Virtual Machines (VMs) which are started and initialized with a defined operating system (from a base image). This is called infrastructure provisioning, and in PhenoMeNal VMs are executed through the Terraform framework [32]. Terraform deploys VM setups to a number of public and private cloud providers including OpenStack, GCE and AWS. The resulting VMs run with a clean install of an operating system including the relevant networking features. Google Kubernetes is used to run software on top of the provisioned VMs [20]]. The Ansible framework is used for the software provisioning layer which performs the deployment of the container daemon and the container orchestrator [33]. Docker is used as the orchestrator daemon for the containers [34].

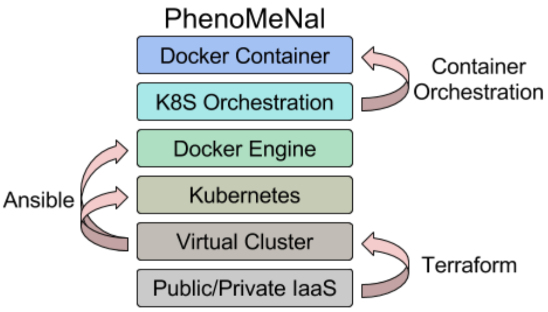

Fig. 4: PhenoMeNal implements various layers to provision containers on top of the e-infrastructure.

PhenoMeNal provides IaaS for three different cloud environments:

1. “local cloud”: local workstations or clusters where data are not allowed to leave the facility.
2. “public cloud”: the flexible use of commercial cloud providers such as GCP and AWS.
3. “shared cloud”: using OpenStack - a free and open-source software platform for cloud computing, ideal for custom environments and research networks.

The cloud infrastructure of PhenoMeNal is based upon containers that are deployed in a Kubernetes environment. Deployment is managed by KubeNow, which is developed by the PhenoMeNal team in order to simplify managing the deployment, including storage, network

and other required services [20,35]. Orchestration is handled by using Helm charts. The storage subsystem is based on the cloud storage file system GlusterFS. Security is guaranteed via HTTPS encryption (SSL certificates issued by Cloudflare). This elastic implementation allows PhenoMeNal to be instantiated on any Kubernetes-based cloud environment [16]. We use a standardized REST API to operate and communicate between the different interfaces [36].

Software tools

The PhenoMeNal portal has an Application Library that allows users to deploy tools as microservices into the cloud infrastructure (Table 1, Fig. 5). The portal is packaged into frontend and backend engines on top of Kubernetes. Orchestration is managed by Helm charts.

Most software tools in PhenoMeNal are compiled from source code and use a variety of programming languages. Linux versions of software tools and user interfaces such as Galaxy are supported in dedicated encapsulated Docker containers which are implemented as minimum-sized microservices. PhenoMeNal currently hosts 100 such projects in its GitHub repository (https://github.com/phnmnl/?q=container). Projects are indicated by the trailing `container-` name and include a ruleset to build and run the containerized tools, as well as data sets for testing and other necessary files.

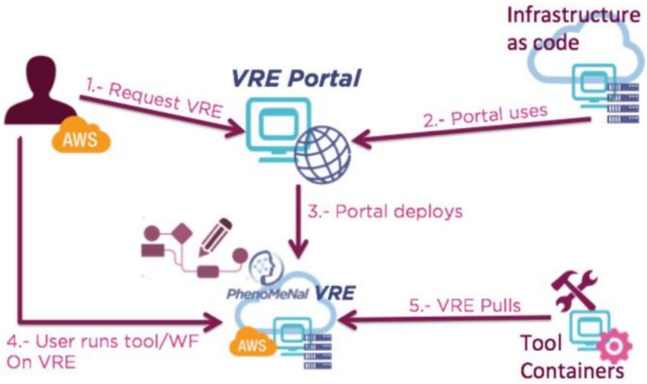

Fig. 5: User interaction with the central PhenoMeNal components. Starting from the CRE Portal users can deploy infrastructure components and services choosing from different cloud providers. When the deployment has been made, services can be used privately.

Table 1: List of external software tools that were incorporated into PhenoMeNal.

**Container Name**	**Description**	**URL**	**Reference**
ArtiMID	Corrects mass isotopomer distribution (MID) for natural isotopes abundance, giving artificial MID	https://github.com/phnmnl/container-artimid	[37]
Batch Correction	Corrects intensities for signal drift and batch-effects	https://github.com/phnmnl/container-batch_correction	[38]

BATMAN	Bayesian Automated Metabolite Analyzer for NMR spectra (BATMAN)	https://github.com/phnmnl/container-batman	[39]
Bioconductor	Metabolomics flavors of Bioconductor	https://github.com/phnmnl/bioc_docker	
Biosigner	Discovery of significant signatures from 'omics data	https://github.com/phnmnl/container-biosigner	[40]
Bruker2BATMAN	Converts Bruker raw files into tabulated txt file for BATMAN	https://github.com/phnmnl/container-bruker2batman	[40]
CAMERA	Collection of annotation related methods for mass spectrometry data	https://github.com/phnmnl/container-camera	[41]
CSI:FingerID	A framework for performing metabolomics identification	https://github.com/phnmnl/container-csifingerid	[42]
DIMSpy	Processing, filtering and analyzing direct-infusion mass spectrometry-based metabolomics and lipidomics data	https://github.com/phnmnl/container-dimspy	[43]
EcoMet	Perform diversity and multivariate analyses for eco-metabolomics data	https://github.com/phnmnl/container-ecomet	[44]
Escher Web	A web-based visualization tool for biological pathways	https://github.com/phnmnl/container-escher-fluxomics	[45]
FingerprintClustering	Performs unsupervised clustering and automatically determination of the best number of clusters	https://github.com/phnmnl/container-fingerprintClustering	[46]
FingerprintSubnetwork	Calculates distances between metabolites in a network	https://github.com/phnmnl/container-fingerprintSubnetwork	[46]
Galaxy	PhenoMeNal version of Galaxy as implemented as a container capable of running inside the Kubernetes container orchestrator	https://github.com/phnmnl/container-galaxy-k8s-runtime	
Generic Filter	Allows to remove all samples and/or variables corresponding to specific values regarding designated factors or numerical variables	https://github.com/phnmnl/container-tool-generic_filter	[11]
IPO	A Tool for automated Optimization of XCMS Parameters	https://github.com/phnmnl/container-ipo	[47]
ISA Extractor	ISA data files extractor	https://github.com/phnmnl/container-isa-extractor	[48]
ISA-Tab Slicer	Using the ISA-API for slicing ISA-Tab metadata	https://github.com/phnmnl/container-isaslicer	[49]
ISA-Tab Validator	ISA-Tab validator	https://github.com/phnmnl/container-isatab-validator	[49]
ISA-Tab to JSON Converter	Converts ISA-Tab to JSON data	https://github.com/phnmnl/container-isatab2json	[49]

ISA-Tab to JSON Validator	Create, manipulate and convert ISA-Tab formatted content and produce validation reports on a ISA-JSON formatted document	https://github.com/phnmnl/container-isajson-validator	[49]
ISA-Tab to W4M	ISA to Workflow4Metabolomics converter	https://github.com/phnmnl/container-isa2w4m	[49]
Iso2Flux	Open source software for steady state 13C Metabolic Flux Analysis	https://github.com/phnmnl/container-iso2flux	
IsoDyn	Simulating the dynamics of metabolites and their isotopic isomers in a central metabolic network using a kinetic model	https://github.com/phnmnl/container-isodyn	[50]
JSON to ISA-Tab Converter	Converts JSON to ISA-Tab format	https://github.com/phnmnl/container-json2isatab	[49]
Jupyter	Light-weight flavor (microservice architecture) of Jupyter	https://github.com/phnmnl/container-jupyter	[51]
LCMS matching	Annotation of MS peaks with matching on a spectral database	https://github.com/phnmnl/container-lcmsmatching	
Luigi	Building complex tasks for scientific notebooks and workflows	https://github.com/phnmnl/container-luigi	
MetaboLab	Non-GUI version of MetaboLab - software for processing and analyzing NMR data	https://github.com/phnmnl/container-metabolab	[52]
MetaboliteIDConverter	Enrich metabolomic data sets with well-known databases identifiers such as InChIKey or ChEBI identifiers	https://github.com/phnmnl/container-MetaboliteIDConverter	[53]
Metabomatching	Identifies metabolites in NMR data using regression, correlation, or PCA spiking	https://github.com/phnmnl/container-metabomatching	[54]
MetExplore	Exploration of metabolic networks	https://github.com/phnmnl/container-MetExploreViz	[46]
MetFrag	Annotation of high precision tandem mass spectra of metabolites	https://github.com/phnmnl/container-metfrag-cli, https://github.com/phnmnl/container-metfrag-cli-batch, https://github.com/phnmnl/container-metfrag-vis	[55]
MIDcor	Correcting 13C mass isotopomers spectra of metabolites for natural occurring isotopes and peaks overlapping	https://github.com/phnmnl/container-midcor	[37]
CDF to MIDcor Converter	Converting CDF files into MIDcor to evaluate the mass spectra of 13C-labeled metabolites	https://github.com/phnmnl/container-cdf2mid	[37]
ms-vfetc	Convert MS vendor export formats to a tabular format	https://github.com/phnmnl/container-ms-vfetc	
MSnbase	Basic plotting, data manipulation and processing of MS-based proteomics and metabolomics data	https://github.com/phnmnl/container-msnbase	

MetaboLights Labs Uploader	Facilities uploading data to MetaboLights Labs	https://github.com/phnmnl/container-mtbl-labs-uploader	[9]
MetaboLights ISA slicer	Selecting subsets of data files from ISA-Tab metadata, based on factor values	https://github.com/phnmnl/container-mtblisa	[49]
MetaboLights Downloader	Download a MetaboLights study and output an ISA-Tab data set. Partial downloading of the data is available through a slicing mechanism.	https://github.com/phnmnl/container-mtbls-dwnld	[11]
MetaboLights Factors Visualization	Create parallel sets plots to show factor values distributions in samples inside an ISA-Tab document or MTBLS study	https://github.com/phnmnl/container-mtbls-factors-viz	[49]
Multivariate	PCA, PLS(-DA) and OPLS(-DA) for multivariate analysis of 'omics data	https://github.com/phnmnl/container-multivariate	[11]
MWTab to ISA-Tab Converter	Generate ISA-Tab document from an NIH Metabolomics Workbench study	https://github.com/phnmnl/container-mw2isa	[10]
mzQuality	A tool to assess the quality of targeted mass spectrometry measurements	https://github.com/phnmnl/container-mzquality	
NMR Integrals	Compares specific metabolite levels in two NMR spectra of blood serum/plasma samples.	https://github.com/phnmnl/container-nmr-integrals	
nmrglue	A module for working with NMR data in Python	https://github.com/phnmnl/container-nmrglue	[56]
nmrML to BATMAN Converter	Convert zipped nmrML files into tabulated txt file for BATMAN	https://github.com/phnmnl/container-nmrML2BATMAN	[39]
nmrML to ISA-Tab Metadata	Convert nmrML metadata to ISA-Tab	https://github.com/phnmnl/container-nmrml2isa	[27]
nmrML Converter	Convert RAW vendor NMR files to nmrML	https://github.com/phnmnl/container-nmrmlconv	[21]
nmrPro	Processing and visualization of NMR data	https://github.com/phnmnl/container-nmrpro	[57]
nmrProcFlow + Rnmr1D	An efficient tool for spectra processing from 1D NMR metabolomics data	https://github.com/phnmnl/container-nmrprocflow	[30]
Normalization	Normalization (operation applied on each (preprocessed) individual spectrum) of preprocessed data	https://github.com/phnmnl/container-normalization	[11]
OpenMS	OpenMS open source software library for LC/MS data management and analyses	https://github.com/phnmnl/container-openms	[58]
Pachyderm	A distributed data-processing tool built on software containers that enables scalable and reproducible pipelines	https://github.com/phnmnl/MTBLS233-Pachyderm	[20]
PAPY	Estimation of statistical power and sample size in metabolic phenotyping	https://github.com/phnmnl/container-papy	[59]

Passatutto	Framework for converting metabolomics identification scores to posterior error probability	https://github.com/phnmnl/container-passatutto https://github.com/phnmnl/container-passatuttopep	[60]
PathwayEnrichement	Predict pathway enrichment into a (human) metabolic network	https://github.com/phnmnl/container-pathwayEnrichment	[46]
ProteoWizard MSConvert	Conversion of mass spectrometry vendor formats to mzML	https://github.com/phnmnl/container-pwiz	[61]
Quality Metrics	Metrics and graphics to check the quality of the data	https://github.com/phnmnl/container-qualitymetrics	[11]
RaMID	Evaluate the mass spectra of 13C-labeled metabolites	https://github.com/phnmnl/container-ramid	
rDolphin	Automatic profiling of 1H 1D NMR data sets	https://github.com/phnmnl/container-rdolphin	
reshape2	Performing cast and melt transformation on data matrices	https://github.com/phnmnl/container-reshape2-cast https://github.com/phnmnl/container-reshape2-melt	
rNMR	Identifying and quantifying metabolites in NMR spectra	https://github.com/phnmnl/container-rnmr	[62]
SBML to JSON Converter	Convert SBML files into JSON format useable in the MetExploreViz visualization module	https://github.com/phnmnl/container-SBML2MetexploreJsonGraph	[63]
SCAMID	Extract MID (mass isotopomer distribution) from mass spectra time course of 13C-labeled metabolites files	https://github.com/phnmnl/container-scamid	[37]
SIMID	Evaluate the mass spectra of 13C-labeled metabolites	https://github.com/phnmnl/container-simid	[37]
SOAP-NMR	Perform 1H-NMR data pre-treatment	https://github.com/phnmnl/container-soap-nmr	
Stadyn	Performs simple statistics on individual samples preparing data for simulation with Isodyn	https://github.com/phnmnl/container-stadyn	[37]
tameNMR	Tools for Analysis of MEtabolomic NMR	https://github.com/phnmnl/container-tamenmr	
Transformation	Transform dataMatrix intensity values	https://github.com/phnmnl/container-transformation	[11]
Univariate	Univariate statistics	https://github.com/phnmnl/container-univariate	[11]
XCMS	Framework for processing and visualization of chromatographically separated and single-spectra mass spectral data	https://github.com/phnmnl/container-xcms, https://github.com/phnmnl/container-xcms-1.x	[64]

Scientific workflows

The Galaxy workflow management system has continued to develop for nearly a decade and is widely regarded as one of the most popular scientific workflow platforms [17,65]. It provides a user-friendly web-based graphical user interface to make it easy for the end-user to configure and run individual modules and entire workflows without programming experience. Galaxy wraps command-line tools and scripts into modules that are launched via the web interface. In PhenoMeNal, we encapsulated each Galaxy module into a Docker container that can be flexibly launched in the cloud infrastructure. In PhenoMeNal, more than 250 modules were implemented and incorporated into Galaxy. Galaxy also supports more powerful features like programmatic access through a REST API and helper libraries to access the running instance of Galaxy [66].

Jupyter, which started its history as the “IPython notebook”, is the most popular among tools commonly referred to as “executable notebooks” or “computational notebooks” [67]. Jupyter lets users combine executable code with results from code executions such as text, tables and figures. Usually Jupyter notebooks are enriched with extended information that explain what the code does. As a result, they are often used for training material and for tutorials. Computational notebooks can also to some extent be used as a way to document code executions and to make executions more reproducible [68].

Luigi is a Python programming library that was originally developed by the company Spotify. It manages pipelines of computations primarily on Big Data systems such as Hadoop and Apache Spark but also supports local execution [67,68]. Luigi is a very flexible library that facilitates building complex pipelines of batch jobs handling dependency resolution, workflow management and visualization. Similarly, Pachyderm allows to process distributed data and to keep track of the data from every stage of the analysis pipeline [20]. With Pachyderm it is possible to track the provenance of results and to accurately reproduce scientific workflows. Luigi and Pachyderm are well suited for complex scientific tasks and easy to use from the python-environment in Jupyter notebooks without additional integration tooling needed.

In PhenoMeNal, we have extended Galaxy, Jupyter, Luigi and Pachyderm in such a way that they can be orchestrated throughout the cloud infrastructure together with the data analysis tools themselves [69]. Six important metabolomics workflows have been fully integrated into PhenoMeNal (Table 2) and more (mzQuality, NMR-BATMAN) are available for testing (Fig. 6) [212].

Table 2: List of workflows which are representative for their respective metabolomics domains (Identification in NMR, Fluxomics, Annotation and identification in MS and eco-metabolomics).

**Workflow name**	**Description**	**References**
1D NMR	Processes 1D NMR experiments from raw data to a data matrix required for visualisation and statistical analysis, building on nmrML and NMRProcFlow. The automatic workflow is based on the MTBLS1 data set, describing urinary changes in type 2 diabetes in humans.	[30,70,71]

Fluxomics	Quantifies steady state fluxes following 13C Metabolic Flux Analysis. The workflow was first based on the analysis of the MTBLS412 data set with 13C tracer data of human umbilical vein endothelial cells (HUVEC) under hypoxia.	[72,73]
LC-MS/MS	Processes, quantifies and annotates/identifies features in mass spectra using MetFrag - a tool which annotates molecules from compound databases of tandem mass spectrometry (MS/MS) spectra. The workflow is based on MTBLS558.	[55,69,74]
Univariate and Multivariate Statistics	Applies univariate and multivariate statistical analysis, and illustrates how data sets may be explored enabling the identification of variables of interest and the construction of predictive models. The workflow is based on MTBLS404.	[11,38]
Eco-Metabolomics	Implementation of a resource demanding metabolomics use case in ecology, used in large field experiments to describe interactions between different species of organisms in remarkable detail. The workflow is based on MTBLS520.	[44]
ISA-Create-Validate-Upload	A workflow to create ISA compliant metadata files based on study design information, augmented with semantic markup as source, implementing UK Phenome center naming conventions. Following validation, the workflow also allows visualization of overall study design and deposition to EMBL-EBI	

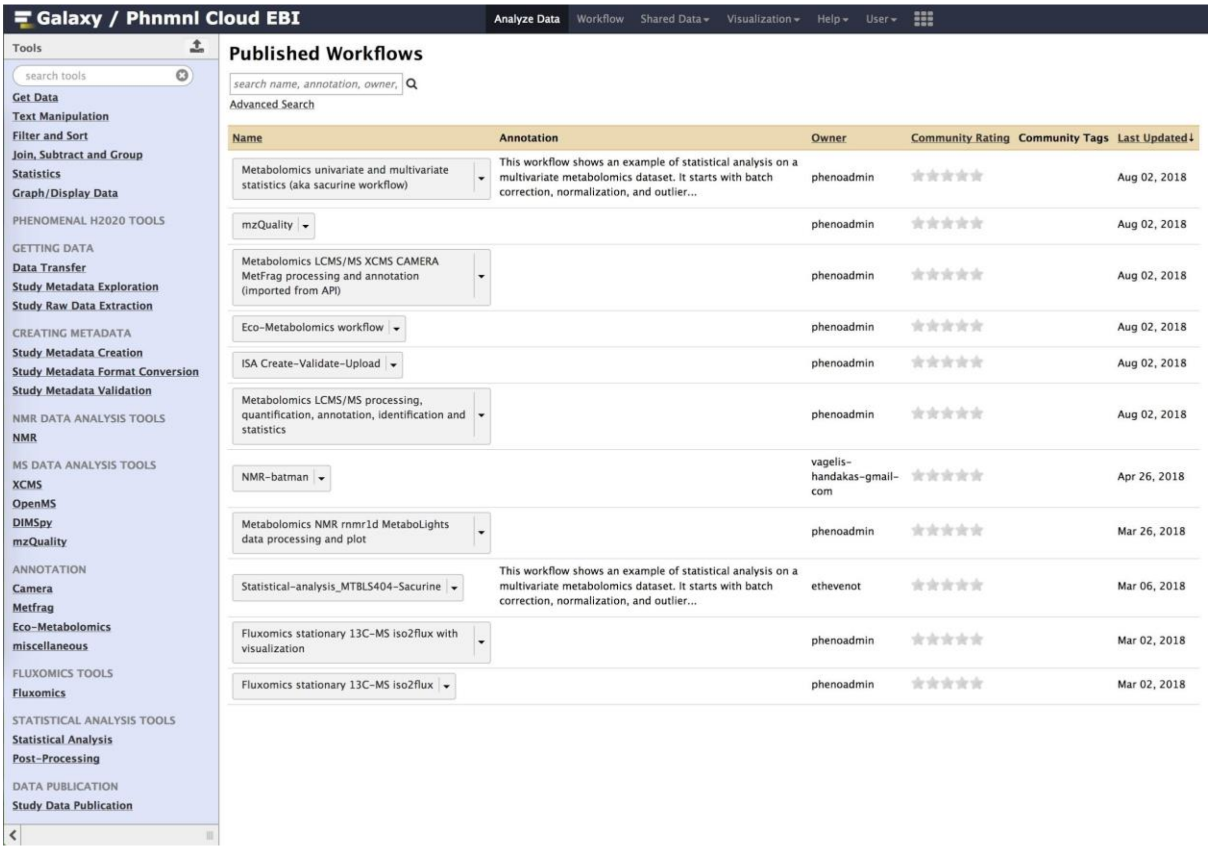

Fig. 6: Screenshot of the available workflows in Galaxy.

Reproducibility

Three strategies are implemented to ensure technical reproducibility using a continuous integration software development framework [25].

- Infrastructure testing: Procedures were implemented to ensure that each individual component (e.g. the deployment process of software containers, resource management, APIs / ABIs) within the infrastructure is interacting correctly with the other components.
- Container testing: Verification that tools, which are packaged into software containers, build and run correctly in the infrastructure. Dependencies within one container and across several interdependent containers are tested.
- Data testing: The output of tools, which process demonstration data, is checked against a data set that is known to contain the expected result. This is being done for both individual tools and for several tools running in a workflow using the workflow testing tool for Galaxy called wft4galaxy [75].

Standardization

PhenoMeNal has implemented several dedicated Galaxy modules that directly retrieve and store ISA-Tab data set descriptors from and to MetaboLights, and can convert between other formats. Native Galaxy composite data types to support ISA-Tab and ISA-JSON have also been integrated, building upon the ISA API [22,27]. The ISA data type allows for the upload of an ISA-Tab archive (a zip file containing the ISA set of files and raw data when available), which then is displayed to the users as a single Galaxy history data set. The integrated Galaxy modules include a MetaboLights downloader and uploader (for ingestion and submission), modules to explore study metadata through queries on study factors, ISA-Tab “slicing” where queries are used to select subsets of data files of interest, as well as format conversion (export to ISA-JSON and W4M) and study metadata validation (Table 1).

The ISAcreate module enables the creation of ISA-compliant archives for deposition to repositories such as MetaboLights. The tool presents users with a graphical user interface (GUI) in which to specify study design information such as a treatment plan, sampling and assay plans, as well as QA/QC plans, critical for quality control. During the specification of these plans, the GUI enables semantic markup through the selection of terms chosen from multiple community-based, open ontologies for describing the different components, namely: UBERON ontology for anatomical parts, OBI for experimental protocols [76], MSIO for metabolomics-specific terms and quality control terminology developed by the PhenoMenal project [77,78], DUO for consent and data use terms [79] thereby addressing essential ethical requirements, and STATO for statistical terms (http://www.stato-ontology.org). Based on the combination of the treatment, sampling and assay, and QA/QC plans, the ISA API calculates the experimental graph relationships between subjects, samples, and data files, prospectively. The resulting output is made available as an ISA-Tab history item in Galaxy.

PhenoMeNal also advanced the specification of the nmrML standard data format [75] and contributed a dedicated composite data type for nmrML to Galaxy. nmrML is used extensively throughout the NMR 1D workflow and conversion from raw format into nmrML is supported via dedicated Galaxy modules. Throughout the entire analysis pipeline, modules of computational workflows were designed to accept standard formats such as mzML, XML

or CSV whenever possible. Furthermore, the e-infrastructure has been designed in such a way that standardized APIs/ABIs are being used for the programmatic interfaces as well as for deploying services. Modern and standardized programming, scripting and meta languages were selected such as Go, HCL, Python, Shell, XML and YAML that are widely used in cloud computing.

Reusability

In an ongoing effort, PhenoMeNal is actively advancing the FAIR criteria for good data management and stewardship [80] to be applied not only to data, but also to software tools and computational workflows (Table 3).

Table 3: Overview of the most important FAIR criteria and implementations suggested for PhenoMeNal data, tools and workflows.

	**Data**	**Tools**	**Workflows**
**(F)indability**	Indexing in domain relevant databases (e.g. MetaboLights)	Indexing in domain relevant software repositories (e.g. the PhenoMeNal App Library, GitHub)	Indexing in workflow management systems such as Galaxy (e.g. PhenoMeNal, W4M), or libraries such as www.myexperiment.org
	Rich descriptions of metadata (e.g. ISA-Tab)	Tool descriptions follow the EDAM ontology	Persistent identifier (e.g. W4M ID, DOI) and intuitive naming patterns
**(A)ccessibility**	Data access and rights management based on e.g. data use ontology (DUO)	Accessible open source licenses	Access to workflow systems can be configured to be shared or restricted
**(I)nteroperability**	Standard formats for experimental metadata (ISA-Tab / ISA-JSON)	Standardized tool descriptions	Standardized workflow format (e.g. Galaxy GA format, Common Workflow Language CWL)
	Domain specific standards for raw data (e.g. mzML, nmrML)	Containerization of software tools	Execution in various software environments (e.g. through the use of containers)

	OboFoundry vocabularies and established domain ontologies to annotate data	EDAM ontology to annotate tools	Workflow annotation ontologies (e.g. Ontology of workflow motifs for annotating workflow specifications [81])
**(R)eusability**	Deposition in data repositories (e.g. MetaboLights) and data indexing sites (e.g. OmicsDI)	Rich documentation and usage guides	Rich documentation and tutorials (e.g. Galaxy tours)

Privacy

PhenoMeNal supports fully anonymized data, which cannot be traced back to individuals in any way [29]. This is done by irreversibly removing metadata associated with each individual and any identifier data may be encoded or hashed. It should not be possible, in a reasonable way, to map back to patient identifiers from the data. In this case, the data is generally free from constraints associated with individual patient consent. However, care must be taken when combining with data from other sources not to unexpectedly allow identification of individuals (such as those with very rare diseases or inborn errors of metabolism). Except for such extreme cases, conventional metabolomics data is considered to be non-identifiable. The primary means of achieving this is to require users to only upload fully anonymized data if processing is in a public cloud.

PhenoMeNal follows the guidelines of the European Union and treats pseudonymized data as identifiable. Pseudonymized data are anonymous to the investigator, but allow trusted third parties to link them back to identifiable individuals through mapping such as hash or code [28]. In these cases, e.g. in a hospital environment, users must deploy PhenoMeNal within a private cloud or cluster behind their institution's firewall. This is consistent with "bringing the compute to the data", avoiding the need to distribute potentially very large and sensitive data sets across networks of limited bandwidth and unknown security. It is important that ELSI considerations are at the heart of the system, allowing scientists to maintain public confidence that their data are being treated according to their wishes.

PhenoMeNal is fully implementing ELSI and GDPR and has implemented both ethical and technical frameworks to regulate and secure the use of private or sensitive data [28,29]. Thus, patients must have given individual consent to the use of their samples for defined metabolomics research purposes. This information in the European Union is protected under the GDPR[1]. Moreover, in PhenoMeNal, metabolite profile data from animal studies are also treated according to ELSI considerations. In the United Kingdom and Germany, stringent

[1] Regulation (EU), 2016/679 of the European Parliament and of the Council of 27 April 2016 on the protection of natural persons with regard to the processing of personal data and on the free movement of such data, and repealing Directive 95/46/EC, 2016, General Data Protection Regulation

regulations on the use of animals are in place. These legislation rules exceed the requirements of the EU Directive for the protection of animals used for scientific purposes. PhenoMeNal further supports the use of combining data and metadata within an ELSI compliant framework [29] and follows an example of an ELSI compliant architecture, the European Genome Phenome Archive (EGA) [82]. PhenoMeNal follows the ELIXIR policy on privacy and has designed a technical secure environment to process data [26].

Security

Open source tools are used throughout the entire e-infrastructure and this promotes community efforts to discover and resolve bugs and security issues. The container build process is steered by the key service Jenkins, which continuously builds the containers and generates reports [25]. On success, authentication container images are pushed to the PhenoMeNal container registry which is publicly available but read only. Cloud provider credentials are not stored in the cloud, but only on the deployer host. The Kubernetes cluster running the continuous integration service Jenkins and the container registry, as well as the portal, runs on a CoreOS container, which is a self-updatable, cluster-aware system with most portions being read-only. It reboots nodes sequentially to avoid lack of availability.

KubeNow is a key component that initializes the cloud infrastructure and configures access to it via Cloudflare (http://cloudflare.com/), providing dynamic DNS services and encryption for all communication with services inside the CRE. The flexible implementation of PhenoMeNal allows the user to decide to not use CloudFlare in which case encryption is disabled. KubeAdm, which manages the setup of Kubernetes, is not reachable at runtime by default. The only way to access it is by having access to the private key stored on the computer on which it was launched.

PhenoMeNal only allows access to standard ports (ssh, http, https and port 44 for the Galaxy Downloader) and implements a cloud-specific firewall for all supported cloud providers. Microservices are designed to be launched on-demand and terminated after completed analysis. The deployment uses a base image to speed up provisioning. The latest incremental security patches are applied to the image on startup. Images are built on a daily basis and tested for deployment, to avoid security patches from introducing any abnormality in the deployment process. All virtual machines accept only SSH keys, no passwords are allowed. For long-running services (e.g. Galaxy, Jupyter) the startup script checks and rejects weak application passwords on launch.

User Resources

A great deal of user resources exist for both PhenoMeNal users and developers, in the form of documentation, tutorials and training videos. The PhenoMeNal Wiki (https://github.com/phnmnl/phenomenal-h2020/wiki) contains detailed documentation on all aspects of PhenoMeNal, including general user guides, workflow and tool tutorials, developer documentation and general information on topics such as security and the e-infrastructure landscape. The PhenoMeNal portal (https://portal.phenomenal-h2020.eu/help) contains help pages generated from the Wiki, which are categorized as User Documentation, Developer Documentation and Workflow Tutorials. Interactive Galaxy tours

are directly integrated in Galaxy (https://public.phenomenal-h2020.eu/tours). Training videos are available at the project’s YouTube page (https://www.youtube.com/channel/UCXGAvsVNQk-aUpckjRC8Ang).

／# Author contributions

KP and JB contributed equally to the writing of the draft of the manuscript. CS conceived, designed and coordinated the project. PM was the technical lead of the project. The consortium members JB, MCap, MCas, PdA, TMDE, RG, AG-B, KH, SH, DJo, FJ, KK, NK, PEK, AL, MC, PM, SN, COD, KP, LP, MEP, MACR, PR-S, PR-M, AR, RR, CR, MvR, NS, RMS, S-AS, DS, OS, VS, EAT, MT, TH, MvV, MRV, RJMW, GZ, CS contributed to the overall project, including tool development. SB, CF, EH, SH, MI, DJa, BK, IK, KK, PEK, SL, JAN, JTMP, AP, LP, RR were additionally involved with external tool development for the project. All authors contributed to, read and approved the final manuscript.

Availability of supporting source code and requirements

Project name: PhenoMeNal
Project home page: http://phenomenal-h2020.eu
Operating system(s): Platform independent
Programming language: Go, HCL, Java, JavaScript, Python, R, Shell, XML, YAML
Other requirements: Linux, Docker, Kubernetes, Terraform, Ansible, Helm
License: MIT license for all code written by the PhenoMeNal project. Individual, Open Source Foundation approved licenses for all containerized tools.

Supporting data

MTBLS1 (NMR1D), MTBLS404 (Uni- and multivariate statistics), MTBLS412 (Fluxomics), MTBLS520 (Eco-Metabolomics), MTBLS558 (MetFrag), available at https://www.ebi.ac.uk/metabolights.

Competing interests

The authors declare that they have no competing interests.

Declarations

Human-derived samples in the data sets MTBLS404 and MTBLS412 were processed according to ELSI guidelines.

Funding

The project was funded by European Commission PhenoMeNal Grant EC654241. The consortium members JB, Mcap, MCas, PdA, TMDE, RG, AG-B, KH, MI, DJo, FJ, NK, PEK, AL, PM, SN, COD, KP, LP, MACR, PR-S, PR-M, AR, RR, CR, TH, MvR, MvV, NS, RMS, S-

AS, DS, OS, VS, EAT, MT, MRV, RJMW, CS received funding from the European Commission PhenoMeNal Grant EC654241.

References

1. Gowda GN, Zhang S, Gu H, Asiago V, Shanaiah N, Raftery D. Metabolomics-based methods for early disease diagnostics. Expert Rev Mol Diagn. 2008;8:617–33.

2. Bundy JG, Davey MP, Viant MR. Environmental metabolomics: a critical review and future perspectives. Metabolomics. 2009;5:3–21.

3. Peters K, Worrich A, Weinhold A, Alka O, Balcke G, Birkemeyer C, et al. Current Challenges in Plant Eco-Metabolomics. Int J Mol Sci. 2018;19:1385.

4. Weber RJM, Lawson TN, Salek RM, Ebbels TMD, Glen RC, Goodacre R, et al. Computational tools and workflows in metabolomics: An international survey highlights the opportunity for harmonisation through Galaxy. Metabolomics [Internet]. 2017 [cited 2018 Sep 3];13. Available from: http://link.springer.com/10.1007/s11306-016-1147-x

5. Joyce AR, Palsson BØ. The model organism as a system: integrating “omics” data sets. Nat Rev Mol Cell Biol. 2006;7:198–210.

6. Rosato A, Tenori L, Cascante M, De Atauri Carulla PR, Martins Dos Santos VAP, Saccenti E. From correlation to causation: analysis of metabolomics data using systems biology approaches. Metabolomics Off J Metabolomic Soc. 2018;14:37.

7. Vignoli A, Ghini V, Meoni G, Licari C, Takis PG, Tenori L, et al. High-throughput metabolomics by 1D NMR. Angew Chem Int Ed Engl. 2018;

8. Goodacre R, Broadhurst D, Smilde AK, Kristal BS, Baker JD, Beger R, et al. Proposed minimum reporting standards for data analysis in metabolomics. Metabolomics. 2007;3:231–41.

9. Haug K, Salek RM, Conesa P, Hastings J, de Matos P, Rijnbeek M, et al. MetaboLights—an open-access general-purpose repository for metabolomics studies and associated meta-data. Nucleic Acids Res. 2013;41:D781–6.

10. Sud M, Fahy E, Cotter D, Azam K, Vadivelu I, Burant C, et al. Metabolomics Workbench: An international repository for metabolomics data and metadata, metabolite standards, protocols, tutorials and training, and analysis tools. Nucleic Acids Res. 2016;44:D463–70.

11. Giacomoni F, Le Corguille G, Monsoor M, Landi M, Pericard P, Petera M, et al. Workflow4Metabolomics: a collaborative research infrastructure for computational metabolomics. Bioinformatics. 2015;31:1493–5.

12. Haug K, Salek RM, Steinbeck C. Global open data management in metabolomics. Curr Opin Chem Biol. 2017;36:58–63.

13. Salek RM, Neumann S, Schober D, Hummel J, Billiau K, Kopka J, et al. COordination of Standards in MetabOlomicS (COSMOS): facilitating integrated metabolomics data access. Metabolomics. 2015;11:1587–97.

14. Lindon JC, Nicholson JK. The emergent role of metabolic phenotyping in dynamic patient stratification. Expert Opin Drug Metab Toxicol. 2014;10:915–9.

15. Sumner LW, Hall RD. Metabolomics across the globe. Metabolomics. 2013;9:258–64.

16. Hoffa C, Mehta G, Freeman T, Deelman E, Keahey K, Berriman B, et al. On the Use of Cloud Computing for Scientific Workflows. 2008 IEEE Fourth Int Conf EScience [Internet]. Indianapolis, IN, USA: IEEE; 2008 [cited 2018 Sep 3]. p. 640–5. Available from: http://ieeexplore.ieee.org/document/4736878/

17. Afgan E, Baker D, van den Beek M, Blankenberg D, Bouvier D, Čech M, et al. The Galaxy platform for accessible, reproducible and collaborative biomedical analyses: 2016 update. Nucleic Acids Res. 2016;44:W3–10.

18. Digan W, Countouris H, Barritault M, Baudoin D, Laurent-Puig P, Blons H, et al. An architecture for genomics analysis in a clinical setting using Galaxy and Docker. GigaScience [Internet]. 2017 [cited 2018 Sep 3];6. Available from: https://academic.oup.com/gigascience/article/doi/10.1093/gigascience/gix099/4557139

19. Goecks J, Nekrutenko A, Taylor J, Galaxy Team T. Galaxy: a comprehensive approach for supporting accessible, reproducible, and transparent computational research in the life sciences. Genome Biol. 2010;11:R86.

20. Novella JA, Khoonsari PE, Herman S, Whitenack D, Capuccini M, Burman J, et al. Container-based bioinformatics with Pachyderm. Wren J, editor. Bioinformatics [Internet]. 2018 [cited 2018 Sep 3]; Available from: https://academic.oup.com/bioinformatics/advance-article/doi/10.1093/bioinformatics/bty699/5068160

21. Rocca-Serra P, Salek RM, Arita M, Correa E, Dayalan S, Gonzalez-Beltran A, et al. Data standards can boost metabolomics research, and if there is a will, there is a way. Metabolomics [Internet]. 2016 [cited 2018 Feb 27];12. Available from: http://link.springer.com/10.1007/s11306-015-0879-3

22. The OBI Consortium, Smith B, Ashburner M, Rosse C, Bard J, Bug W, et al. The OBO Foundry: coordinated evolution of ontologies to support biomedical data integration. Nat Biotechnol. 2007;25:1251–5.

23. Steinbeck C, Conesa P, Haug K, Mahendraker T, Williams M, Maguire E, et al. MetaboLights: towards a new COSMOS of metabolomics data management. Metabolomics. 2012;8:757–60.

24. Gil Y, Deelman E, Ellisman M, Fahringer T, Fox G, Gannon D, et al. Examining the Challenges of Scientific Workflows. Computer. 2007;40:24–32.

25. Duvall PM, Matyas S, Glover A. Continuous integration: improving software quality and reducing risk. Upper Saddle River, NJ: Addison-Wesley; 2007.

26. van Rijswijk M, Beirnaert C, Caron C, Cascante M, Dominguez V, Dunn WB, et al. The future of metabolomics in ELIXIR. F1000Research. 2017;6:1649.

27. Rocca-Serra P, Brandizi M, Maguire E, Sklyar N, Taylor C, Begley K, et al. ISA software suite: supporting standards-compliant experimental annotation and enabling curation at the community level. Bioinformatics. 2010;26:2354–6.

28. Sariyar M, Schluender I, Smee C, Suhr S. Sharing and Reuse of Sensitive Data and Samples: Supporting Researchers in Identifying Ethical and Legal Requirements. Biopreservation Biobanking. 2015;13:263–70.

29. Heatherly R, Rasmussen LV, Peissig PL, Pacheco JA, Harris P, Denny JC, et al. A multi-institution evaluation of clinical profile anonymization. J Am Med Inform Assoc. 2016;23:e131–7.

30. Jacob D, Deborde C, Lefebvre M, Maucourt M, Moing A. NMRProcFlow: a graphical and interactive tool dedicated to 1D spectra processing for NMR-based metabolomics. Metabolomics [Internet]. 2017 [cited 2018 Feb 27];13. Available from: http://link.springer.com/10.1007/s11306-017-1178-y

31. Mell PM, Grance T. The NIST definition of cloud computing [Internet]. Gaithersburg, MD: National Institute of Standards and Technology; 2011. Report No.: NIST SP 800-145. Available from: https://nvlpubs.nist.gov/nistpubs/Legacy/SP/nistspecialpublication800-145.pdf

32. Brikman Y. Terraform: Writing Infrastructure as Code. [Internet]. Sebastopol: O'Reilly Media; 2017 [cited 2018 Sep 3]. Available from: http://public.eblib.com/choice/publicfullrecord.aspx?p=4822376

33. Hanwell MD, de Jong WA, Harris CJ. Open chemistry: RESTful web APIs, JSON, NWChem and the modern web application. J Cheminformatics [Internet]. 2017 [cited 2018 Sep 3];9. Available from: https://jcheminf.springeropen.com/articles/10.1186/s13321-017-0241-z

34. Newman S. Building microservices: designing fine-grained systems. First Edition. Beijing Sebastopol, CA: O'Reilly Media; 2015.

35. Capuccini M, Larsson A, Carone M, Novella JA, Sadawi N, Gao J, et al. KubeNow: an On-Demand Cloud-Agnostic Platform for Microservices-Based Research Environments. ArXiv180506180 Cs [Internet]. 2018 [cited 2018 Sep 3]; Available from: http://arxiv.org/abs/1805.06180

36. Erl T, editor. SOA with REST: principles, patterns & constraints for building enterprise solutions with REST. Upper Saddle River, NJ: Prentice Hall; 2012.

37. Selivanov VA, Benito A, Miranda A, Aguilar E, Polat IH, Centelles JJ, et al. MIDcor, an R-program for deciphering mass interferences in mass spectra of metabolites enriched in stable isotopes. BMC Bioinformatics [Internet]. 2017 [cited 2018 Sep 3];18. Available from: http://bmcbioinformatics.biomedcentral.com/articles/10.1186/s12859-017-1513-3

38. Thévenot EA, Roux A, Xu Y, Ezan E, Junot C. Analysis of the Human Adult Urinary Metabolome Variations with Age, Body Mass Index, and Gender by Implementing a Comprehensive Workflow for Univariate and OPLS Statistical Analyses. J Proteome Res. 2015;14:3322–35.

39. Hao J, Liebeke M, Astle W, De Iorio M, Bundy JG, Ebbels TMD. Bayesian deconvolution and quantification of metabolites in complex 1D NMR spectra using BATMAN. Nat Protoc. 2014;9:1416–27.

40. Rinaudo P, Boudah S, Junot C, Thévenot EA. biosigner: A New Method for the Discovery of Significant Molecular Signatures from Omics Data. Front Mol Biosci [Internet]. 2016 [cited 2018 Sep 3];3. Available from: http://journal.frontiersin.org/Article/10.3389/fmolb.2016.00026/abstract

41. Kuhl C, Tautenhahn R, Böttcher C, Larson TR, Neumann S. CAMERA: An Integrated Strategy for Compound Spectra Extraction and Annotation of Liquid Chromatography/Mass Spectrometry Data Sets. Anal Chem. 2012;84:283–9.

42. Dührkop K, Shen H, Meusel M, Rousu J, Böcker S. Searching molecular structure databases with tandem mass spectra using CSI:FingerID. Proc Natl Acad Sci. 2015;112:12580–5.

43. Southam AD, Weber RJM, Engel J, Jones MR, Viant MR. A complete workflow for high-resolution spectral-stitching nanoelectrospray direct-infusion mass-spectrometry-based metabolomics and lipidomics. Nat Protoc. 2017;12:255–73.

44. Peters K, Gorzolka K, Bruelheide H, Neumann S. Computational workflow to study the seasonal variation of secondary metabolites in nine different bryophytes. Sci Data. 2018;5:180179.

45. King ZA, Dräger A, Ebrahim A, Sonnenschein N, Lewis NE, Palsson BO. Escher: A Web Application for Building, Sharing, and Embedding Data-Rich Visualizations of Biological Pathways. Gardner PP, editor. PLOS Comput Biol. 2015;11:e1004321.

46. Cottret L, Frainay C, Chazalviel M, Cabanettes F, Gloaguen Y, Camenen E, et al. MetExplore: collaborative edition and exploration of metabolic networks. Nucleic Acids Res. 2018;46:W495–502.

47. Libiseller G, Dvorzak M, Kleb U, Gander E, Eisenberg T, Madeo F, et al. IPO: a tool for automated optimization of XCMS parameters. BMC Bioinformatics [Internet]. 2015 [cited 2018 May 17];16. Available from: http://bmcbioinformatics.biomedcentral.com/articles/10.1186/s12859-015-0562-8

48. González-Beltrán A, Neumann S, Maguire E, Sansone S-A, Rocca-Serra P. The Risa R/Bioconductor package: integrative data analysis from experimental metadata and back again. BMC Bioinformatics. 2014;15:S11.

49. Sansone S-A, Rocca-Serra P, Field D, Maguire E, Taylor C, Hofmann O, et al. Toward interoperable bioscience data. Nat Genet. 2012;44:121–6.

50. Selivanov VA, Vizán P, Mollinedo F, Fan TW, Lee PW, Cascante M. Edelfosine-induced metabolic changes in cancer cells that precede the overproduction of reactive oxygen species and apoptosis. BMC Syst Biol. 2010;4:135.

51. Perez F, Granger BE. IPython: A System for Interactive Scientific Computing. Comput Sci Eng. 2007;9:21–9.

52. Ludwig C, Günther UL. MetaboLab - advanced NMR data processing and analysis for metabolomics. BMC Bioinformatics. 2011;12:366.

53. Wohlgemuth G, Haldiya PK, Willighagen E, Kind T, Fiehn O. The Chemical Translation Service--a web-based tool to improve standardization of metabolomic reports. Bioinformatics. 2010;26:2647–8.

54. Rueedi R, Mallol R, Raffler J, Lamparter D, Friedrich N, Vollenweider P, et al. Metabomatching: Using genetic association to identify metabolites in proton NMR spectroscopy. Ouzounis CA, editor. PLOS Comput Biol. 2017;13:e1005839.

55. Ruttkies C, Schymanski EL, Wolf S, Hollender J, Neumann S. MetFrag relaunched: incorporating strategies beyond in silico fragmentation. J Cheminformatics [Internet]. 2016 [cited 2018 Sep 3];8. Available from: http://www.jcheminf.com/content/8/1/3

56. Helmus JJ, Jaroniec CP. Nmrglue: an open source Python package for the analysis of multidimensional NMR data. J Biomol NMR. 2013;55:355–67.

57. Mohamed A, Nguyen CH, Mamitsuka H. NMRPro: an integrated web component for interactive processing and visualization of NMR spectra. Bioinformatics. 2016;32:2067–8.

58. Sturm M, Bertsch A, Gröpl C, Hildebrandt A, Hussong R, Lange E, et al. OpenMS – An open-source software framework for mass spectrometry. BMC Bioinformatics. 2008;9:163.

59. Blaise BJ, Correia G, Tin A, Young JH, Vergnaud A-C, Lewis M, et al. Power Analysis and Sample Size Determination in Metabolic Phenotyping. Anal Chem. 2016;88:5179–88.

60. Scheubert K, Hufsky F, Petras D, Wang M, Nothias L-F, Dührkop K, et al. Significance estimation for large scale metabolomics annotations by spectral matching. Nat Commun [Internet]. 2017 [cited 2018 Sep 3];8. Available from: http://www.nature.com/articles/s41467-017-01318-5

61. Chambers MC, Maclean B, Burke R, Amodei D, Ruderman DL, Neumann S, et al. A cross-platform toolkit for mass spectrometry and proteomics. Nat Biotechnol. 2012;30:918–20.

62. Lewis IA, Schommer SC, Markley JL. rNMR: open source software for identifying and quantifying metabolites in NMR spectra. Magn Reson Chem. 2009;47:S123–6.

63. Rodriguez N, Thomas A, Watanabe L, Vazirabad IY, Kofia V, Gómez HF, et al. JSBML 1.0: providing a smorgasbord of options to encode systems biology models: Table 1. Bioinformatics. 2015;31:3383–6.

64. Benton HP, Wong DM, Trauger SA, Siuzdak G. XCMS ^{2}: Processing Tandem Mass Spectrometry Data for Metabolite Identification and Structural Characterization. Anal Chem. 2008;80:6382–9.

65. Nekrutenko A, Taylor J. Next-generation sequencing data interpretation: enhancing reproducibility and accessibility. Nat Rev Genet. 2012;13:667–72.

66. Sloggett C, Goonasekera N, Afgan E. BioBlend: automating pipeline analyses within Galaxy and CloudMan. Bioinformatics. 2013;29:1685–6.

67. Thomas K, Benjamin R-K, Fernando P, Brian G, Matthias B, Jonathan F, et al. Jupyter Notebooks – a publishing format for reproducible computational workflows. Stand Alone. 2016;87–90.

68. Lampa S, Alvarsson J, Spjuth O. Towards agile large-scale predictive modelling in drug discovery with flow-based programming design principles. J Cheminformatics [Internet]. 2016 [cited 2018 Sep 3];8. Available from: http://jcheminf.springeropen.com/articles/10.1186/s13321-016-0179-6

69. Emami Khoonsari P, Moreno P, Bergmann S, Burman J, Capuccini M, Carone M, et al. Interoperable and scalable data analysis with microservices: Applications in Metabolomics. 2018 [cited 2018 Sep 3]; Available from: http://biorxiv.org/lookup/doi/10.1101/213603

70. Schober D, Jacob D, Wilson M, Cruz JA, Marcu A, Grant JR, et al. nmrML: A Community Supported Open Data Standard for the Description, Storage, and Exchange of NMR Data. Anal Chem. 2018;90:649–56.

71. Salek RM, Maguire ML, Bentley E, Rubtsov DV, Hough T, Cheeseman M, et al. A metabolomic comparison of urinary changes in type 2 diabetes in mouse, rat, and human. Physiol Genomics. 2007;29:99–108.

72. Buescher JM, Antoniewicz MR, Boros LG, Burgess SC, Brunengraber H, Clish CB, et al. A roadmap for interpreting 13 C metabolite labeling patterns from cells. Curr Opin Biotechnol. 2015;34:189–201.

73. Niedenführ S, Wiechert W, Nöh K. How to measure metabolic fluxes: a taxonomic guide for 13 C fluxomics. Curr Opin Biotechnol. 2015;34:82–90.

74. Herman S, Khoonsari PE, Tolf A, Steinmetz J, Zetterberg H, Åkerfeldt T, et al. Integration of magnetic resonance imaging and protein and metabolite CSF measurements to enable early diagnosis of secondary progressive multiple sclerosis. Theranostics. 2018;8:4477–90.

75. Piras ME, Pireddu L, Zanetti G. wft4galaxy: a workflow testing tool for galaxy. Bioinformatics. 2017;33:3805–7.

76. Bandrowski A, Brinkman R, Brochhausen M, Brush MH, Bug B, Chibucos MC, et al. The Ontology for Biomedical Investigations. Xue Y, editor. PLOS ONE. 2016;11:e0154556.

77. Sansone S-A, Rocca-Serra P, Field D, Maguire E, Taylor C, Hofmann O, et al. Toward interoperable bioscience data. Nat Genet. 2012;44:121–6.

78. Sansone S-A, Schober D, Atherton HJ, Fiehn O, Jenkins H, Rocca-Serra P, et al. Metabolomics standards initiative: ontology working group work in progress. Metabolomics. 2007;3:249–56.

79. Dyke SOM, Philippakis AA, Rambla De Argila J, Paltoo DN, Luetkemeier ES, Knoppers BM, et al. Consent Codes: Upholding Standard Data Use Conditions. Barsh GS, editor. PLOS Genet. 2016;12:e1005772.

80. Wilkinson MD, Dumontier M, Aalbersberg IjJ, Appleton G, Axton M, Baak A, et al. The FAIR Guiding Principles for scientific data management and stewardship. Sci Data. 2016;3:160018.

81. Cohen-Boulakia S, Belhajjame K, Collin O, Chopard J, Froidevaux C, Gaignard A, et al. Scientific workflows for computational reproducibility in the life sciences: Status, challenges and opportunities. Future Gener Comput Syst. 2017;75:284–98.

82. Lappalainen I, Almeida-King J, Kumanduri V, Senf A, Spalding JD, ur-Rehman S, et al. The European Genome-phenome Archive of human data consented for biomedical research. Nat Genet. 2015;47:692–5.

Author contact addresses

Name	email	ORCID	Institute
Kristian Peters	kpeters@ipb-halle.de	0000-0002-4321-0257	Leibniz Institute of Plant Biochemistry, Stress and Developmental Biology, Weinberg 3, 06120 Halle (Saale), Germany
James Bradbury	j.bradbury@bham.ac.uk		School of Biosciences, University of Birmingham, Edgbaston, Birmingham, B15 2TT, United Kingdom
Sven Bergmann	sven.bergmann@unil.ch		Department of Computational Biology, Rue du Bugnon 27, 1011 Lausanne, Switzerland
Marco Capuccini	marco.capuccini@it.uu.se	0000-0002-4851-759X	Division of Scientific Computing, Department of Information Technology, Uppsala University, Sweden. Department of Pharmaceutical Biosciences, Uppsala University, Sweden
Marta Cascante	martacascante@ub.edu	0000-0002-2062-4633	Department of Biochemistry and Molecular Biomedicine, Universitat de Barcelona; Centro de Investigación Biomédica en Red de Enfermedades Hepáticas y Digestivas (CIBEREHD), Instituto de Salud Carlos III (ISCIII), Spain.
Pedro de Atauri	pde_atauri@ub.edu	0000-0002-7754-7851	Department of Biochemistry and Molecular Biomedicine, Universitat de Barcelona; Centro de Investigación

			Biomédica en Red de Enfermedades Hepáticas y Digestivas (CIBEREHD), Instituto de Salud Carlos III (ISCIII), Spain
Tim Ebbels	timebbels@gmail.com		Department of Surgery & Cancer, Imperial College London, South Kensington, London, SW7 2AZ, United Kingdom
Carles Foguet	cfoguet@ub.edu	0000-0001-8494-9595	Department of Biochemistry and Molecular Biomedicine, Universitat de Barcelona; Centro de Investigación Biomédica en Red de Enfermedades Hepáticas y Digestivas (CIBEREHD), Instituto de Salud Carlos III (ISCIII), Spain.
Robert Glen	robert.c.glen@gmail.com		Centre for Molecular Informatics, Department of Chemistry, Lensfield Road, Cambridge CB2 1EW, UK
Alejandra N. Gonzalez-Beltran	alejandra.gonzalezbeltran@oerc.ox.ac.uk	0000-0003-3499-8262	Oxford e-Research Centre, Department of Engineering Science, University of Oxford, 7 Keble Road, OX1 3QG, Oxford, UK.
Evangelos Handakas	e.chandakas@imperial.ac.uk		Department of Surgery & Cancer, Imperial College London, South Kensington, London, SW7 2AZ, United Kingdom
Thomas Hankemeier	hankemeier@lacdr.leidenuniv.nl	0000-0001-7871-2073	Division of Systems Biomedicine and Pharmacology, Leiden Academic Centre for Drug Research (LACDR), Leiden University, Leiden, 2333 CC, The Netherlands.

Stephanie Herman	stephanie.herman@medsci.uu.se	0000-0001-9382-3273	Department of Medical Sciences, Clinical Chemistry, Uppsala University, 751 85 Uppsala, Sweden
Kenneth Haug	kenneth@ebi.ac.uk	0000-0003-3168-4145	European Molecular Biology Laboratory, European Bioinformatics Institute (EMBL-EBI), Wellcome Genome Campus, Hinxton, Cambridge CB10 1SD, UK
Petr Holub	petr.holub@bbmri-eric.eu	0000-0002-5358-616X	BBMRI-ERIC, Graz, Austria
Massimiliano Izzo	massimiliano.izzo@oerc.ox.ac.uk	0000-0002-8100-6142	Oxford e-Research Centre, Department of Engineering Science, University of Oxford, 7 Keble Road, OX1 3QG, Oxford, UK.
Daniel Jacob	djacob@u-bordeaux.fr		INRA, Univ. Bordeaux, UMR1332 Fruit Biology and Pathology, Metabolome Facility of Bordeaux Functional Genomics Center, MetaboHUB, IBVM, Centre INRA Bordeaux, 71 av Edouard Bourlaux, F-33140 Villenave d'Ornon, France
David Johnson	david.johnson@oerc.ox.ac.uk	0000-0002-2323-6847	Oxford e-Research Centre, Department of Engineering Science, University of Oxford, 7 Keble Road, OX1 3QG, Oxford, UK.
Fabien Jourdan	fabienjourdan@gmail.com		INRA - French National Institute for Agricultural Research, UMR1331, Toxalim, Research Centre in Food Toxicology, Toulouse, France
Namrata Kale	namrat@ebi.ac.uk	0000-0002-4255-8104	European Molecular Biology Laboratory, European Bioinformatics Institute (EMBL-EBI), Wellcome

			Genome Campus, Hinxton, Cambridge CB10 1SD, UK
Ibrahim Karaman	i.karaman@imperial.ac.uk	0000-0001-9341-8155	Department of Epidemiology and Biostatistics, School of Public Health, Imperial College London, St. Mary's Campus, Norfolk Place, W2 1PG, London, United Kingdom
Bita Khalili	bita.khalili@unil.ch		Department of Computational Biology, Rue du Bugnon 27, 1011 Lausanne, Switzerland
Payam Emami Khoonsari	payam.emami@medsci.uu.se	0000-0002-4137-5517	Department of Medical Sciences, Clinical Chemistry, Uppsala University, 751 85 Uppsala, Sweden
Kim Kultima	kim.kultima@medsci.uu.se	0000-0002-0680-1410	Department of Medical Sciences, Clinical Chemistry, Uppsala University, 751 85 Uppsala, Sweden
Samuel Lampa	samuel.lampa@farmbio.uu.se	0000-0001-6740-9212	Department of Pharmaceutical Biosciences, Uppsala University, Box 591, 751 24 Uppsala, Sweden
Anders Larsson	anders.larsson@icm.uu.se	0000-0002-2096-8102	National Bioinformatics Infrastructure Sweden, Uppsala University, Uppsala, Sweden Department of Pharmaceutical Biosciences, Uppsala University, Uppsala, Sweden
Pablo Moreno	pmoreno@ebi.ac.uk	0000-0002-9856-1679	European Molecular Biology Laboratory, European Bioinformatics Institute (EMBL-EBI), Wellcome Genome Campus, Hinxton, Cambridge CB10 1SD, UK

Steffen Neumann	sneumann@ipb-halle.de	0000-0002-7899-7192	Leibniz Institute of Plant Biochemistry, Stress and Developmental Biology, Weinberg 3, 06120 Halle (Saale), Germany
Jon Ander Novella	jon.novella@farmbio.uu.se	0000-0002-2187-5426	Department of Pharmaceutical Biosciences, Uppsala University, Sweden.
Claire O'Donovan	odonovan@ebi.ac.uk	0000-0001-8051-7429	European Molecular Biology Laboratory, European Bioinformatics Institute (EMBL-EBI), Wellcome Genome Campus, Hinxton, Cambridge CB10 1SD, UK
Jake TM Pearce	jake.pearce@gmail.com	0000-0003-4637-3171	Department of Surgery & Cancer, Imperial College London, South Kensington, London, SW7 2AZ, United Kingdom
Alina Peluso	a.peluso@imperial.ac.uk	0000-0003-2895-0406	Department of Surgery & Cancer, Imperial College London, South Kensington, London, SW7 2AZ, United Kingdom
Luca Pireddu	luca.pireddu@crs4.it	0000-0002-4663-5613	Distributed Computing Group, CRS4, Italy
Marco Enrico Piras	marcoenrico.piras@crs4.it	0000-0002-5207-0030	Distributed Computing Group, CRS4, Italy
Michelle AC Reed	m.thompson@bham.ac.uk	0000-0002-0667-4490	College of Medical and Dental Sciences, University of Birmingham, Edgbaston, Birmingham, B15 2TT, United Kingdom
Philippe Rocca-Serra	philippe.rocca-serra@oerc.ox.ac.uk	0000-0001-9853-5668	Oxford e-Research Centre, Department of Engineering Science, University of Oxford, 7 Keble Road, OX1

			3QG, Oxford, UK.
Pierrick Roger	pierrick.roger@gmail.com		CEA, LIST, Laboratory for Data Analysis and Systems' Intelligence, MetaboHUB, Gif-Sur-Yvette F-91191, France
Antonio Rosato	rosato@cerm.unifi.it	0000-0001-6172-0368	Magnetic Resonance Center (CERM) and Department of Chemistry, University of Florence and CIRMMP, 50019 Sesto Fiorentino, Florence, Italy
Rico Rueedi	rico.rueedi@unil.ch	0000-0002-6713-2214	Department of Computational Biology, Rue du Bugnon 27, 1011 Lausanne, Switzerland
Christoph Ruttkies	ruttkies@web.de	0000-0002-8621-8689	Leibniz Institute of Plant Biochemistry, Stress and Developmental Biology, Weinberg 3, 06120 Halle (Saale), Germany
Noureddin Sadawi	noureddin.sadawi@gmail.com	0000-0002-2195-8264	Faculty of Medicine, Department of Surgery & Cancer Department of Surgery and Cancer, Imperial College London, London, UK
Reza M Salek	reza.salek@ebi.ac.uk	0000-0001-8604-1732	European Bioinformatics Institute (EMBL-EBI), European Molecular Biology Laboratory, Wellcome Genome Campus, Hinxton, Cambridge CB10 1SD, U.K.
Susanna-Assunta Sansone	susanna-assunta.sansone@oerc.ox.ac.uk	0000-0001-5306-5690	Oxford e-Research Centre, Department of Engineering Science, University of Oxford, 7 Keble Road, OX1 3QG, Oxford, UK.
Vitaly Selivanov	seliv55@gmail.com	0000-0002-7937-9249	Department of

			Biochemistry and Molecular Biomedicine, Universitat de Barcelona; Centro de Investigación Biomédica en Red de Enfermedades Hepáticas y Digestivas (CIBEREHD), Instituto de Salud Carlos III (ISCIII), Spain.
Ola Spjuth	ola.spjuth@farmbio.uu.se	0000-0002-8083-2864	Department of Pharmaceutical Biosciences, Uppsala University, Box 591, 751 24 Uppsala, Sweden
Daniel Schober	DanielSawstMsi@gmail.com	0000-0001-8014-6648	Leibniz Institute of Plant Biochemistry, Stress and Developmental Biology, Weinberg 3, 06120 Halle (Saale), Germany
Etienne A. Thévenot	etienne.thevenot@cea.fr	0000-0003-1019-4577	CEA, LIST, Laboratory for Data Analysis and Systems' Intelligence, MetaboHUB, Gif-Sur-Yvette F-91191, France
Mattia Tomasoni	mattia.tomasoni@unil.ch		Department of Computational Biology, Rue du Bugnon 27, 1011 Lausanne, Switzerland
Merlijn van Rijswijk	merlijn.van.rijswijk@dtls.nl	0000-0002-1067-7766	ELIXIR-NL, Dutch Techcentre for Life Sciences, Utrecht, 3503 RM, Netherlands / Netherlands Metabolomics Center, Leiden, 2333 CC, Netherlands
Michael van Vliet	m.s.vanvliet@lacdr.leidenuniv.nl	0000-0002-5034-5766	Division of Systems Biomedicine and Pharmacology, Leiden Academic Centre for Drug Research (LACDR), Leiden University, Leiden,

			2333 CC, The Netherlands
Mark Viant	m.viant@bham.ac.uk	0000-0001-5898-4119	School of Biosciences, University of Birmingham, Edgbaston, Birmingham, B15 2TT, United Kingdom
Ralf Weber	r.j.weber@bham.ac.uk		School of Biosciences, University of Birmingham, Edgbaston, Birmingham, B15 2TT, United Kingdom
Gianluigi Zanetti	gianluigi.zanetti@crs4.it	0000-0003-1683-7350	Distributed Computing Group, CRS4, Italy
Christoph Steinbeck	christoph.steinbeck@uni-jena.de	0000-0001-6966-0814	Institute for Inorganic and Analytical Chemistry, Friedrich-Schiller-University, 07743 Jena, Germany

PDF of manuscript

Click here to access/download
Supplementary Material
giga-409151.pdf
