# Supplementary material for: PhenoMeNal: processing and analysis of metabolomics data in the cloud
Source: Gigascience. 2018 Dec 7;8(2):giy149. doi: 10.1093/gigascience/giy149 (PMC6377398; doi:10.1093/gigascience/giy149)
Supplement: GIGA-D-18-00347_Revision_1.pdf [file giy149_giga-d-18-00347_revision_1.pdf]

GigaScience

PhenoMeNal: Processing and analysis of Metabolomics data in the Cloud

--Manuscript Draft--

**Manuscript Number:**	GIGA-D-18-00347R1
**Full Title:**	PhenoMeNal: Processing and analysis of Metabolomics data in the Cloud
**Article Type:**	Technical Note
**Funding Information:**	H2020 European Research Council (EC654241) — Not applicable
**Abstract:**	Background: Metabolomics is the comprehensive study of a multitude of small molecules to gain insight into an organism's metabolism. The research field is dynamic and expanding with applications across biomedical, biotechnological and many other applied biological domains. Its computationally-intensive nature has driven requirements for open data formats, data repositories and data analysis tools. However, the rapid progress has resulted in a mosaic of independent – and sometimes incompatible – analysis methods that are difficult to connect into a useful and complete data analysis solution.<br>Findings: PhenoMeNal (Phenome and Metabolome aNalysis) is an advanced and complete solution to set up Infrastructure-as-a-Service (IaaS) that brings workflow-oriented, interoperable metabolomics data analysis platforms into the cloud. PhenoMeNal seamlessly integrates a wide array of existing open source tools which are tested and packaged as Docker containers through the project's continuous integration process and deployed based on a kubernetes orchestration framework. It also provides a number of standardized, automated and published analysis workflows in the user interfaces Galaxy, Jupyter, Luigi and Pachyderm.<br>Conclusions: PhenoMeNal constitutes a keystone solution in cloud e-infrastructures available for metabolomics. PhenoMeNal is a unique and complete solution for setting up cloud e-infrastructures through easy-to-use web interfaces that can be scaled to any custom public and private cloud environment. By harmonizing and automating software installation and configuration and through ready-to-use scientific workflow user interfaces, PhenoMeNal has succeeded in providing scientists with workflow-driven, reproducible and shareable metabolomics data analysis platforms which are interfaced through standard data formats, representative datasets, versioned, and have been tested for reproducibility and interoperability. The elastic implementation of PhenoMeNal further allows easy adaptation of the infrastructure to other application areas and 'omics research domains.
**Corresponding Author:**	Kristian Peters<br>Leibniz-Institut für Pflanzenbiochemie<br>Halle, Sachsen-Anhalt GERMANY
**Corresponding Author Secondary Information:**	
**Corresponding Author's Institution:**	Leibniz-Institut für Pflanzenbiochemie
**Corresponding Author's Secondary Institution:**	
**First Author:**	James Bradbury
**First Author Secondary Information:**	
**Order of Authors:**	James Bradbury
	Sven Bergmann
	Marco Capuccini
	Marta Cascante
	Pedro de Atauri
	Tim Ebbels

Powered by Editorial Manager® and ProduXion Manager® from Aries Systems Corporation

	Carles Foguet
	Robert C Glen
	Alejandra Gonzalez-Beltran
	Evangelos Handakas
	Thomas Hankemeier
	Stephanie Herman
	Kenneth Haug
	Petr Holub
	Massimiliano Izzo
	Daniel Jacob
	David Johnson
	Fabien Jourdan
	Namrata Kale
	Ibrahim Karaman
	Bita Khalili
	Payam Emami Khoonsari
	Kim Kultima
	Samuel Lampa
	Anders Larsson
	Pablo Moreno
	Steffen Neumann
	Jon Ander Novella
	Claire O'Donovan
	Jake TM Pearce
	Alina Peluso
	Luca Pireddu
	Marco Enrico Piras
	Michelle AC Reed
	Philippe Rocca-Serra
	Pierrick Roger
	Antonio Rosato
	Rico Rueedi
	Christoph Ruttkies
	Noureddin Sadawi
	Reza Salek
	Susanna-Assunta Sansone
	Vitaly Selivanov
	Ola Spjuth
	Daniel Schober
	Etienne A. Thévenot
	Mattia Tomasoni

Powered by Editorial Manager® and ProduXion Manager® from Aries Systems Corporation

	Merlijn van Rijswijk
	Michael van Vliet
	Mark Viant
	Ralf Weber
	Gianluigi Zanetti
	Christoph Steinbeck, Dr. rer. net.
	Kristian Peters
	Ulrich L. Günther
	Christian Ludwig
**Order of Authors Secondary Information:**	
**Response to Reviewers:**	First of all, we thank the reviewers and editors for the very helpful and very proficient comments. We have revised the manuscript according to the comments.<br><br>In order to coordinate the revision process between all the involved authors, we have created a Google doc which contains the changes from the individual authors:<br><br>https://docs.google.com/document/d/1OlDE-05TFzP6NfITJkMBNm7tAy5J5j9aTfLP6ArUErA/edit<br><br>Changes were then transferred to a Word document and the references updated.<br><br><br>Dear Prof. Dr. Steinbeck,<br><br>Your manuscript "PhenoMeNal: Processing and analysis of Metabolomics data in the Cloud" (GIGA-D-18-00347) has been assessed by our reviewers. Based on these reports, and my own assessment as Editor, I am pleased to inform you that it is potentially acceptable for publication in GigaScience, once you have carried out some essential revisions suggested by our reviewers.<br><br>A comparison is required against other tools such as MetaboAnalyst, XCMS Online, Galaxy and other cloud-based metabolomics tools, as well as including a few sentences to highlight it's uniqueness and novelty would be beneficial.<br><br>We have added a comparison in the introduction.<br><br>Their reports, together with any other comments, are below. Please also take a moment to check our website at https://giga.editorialmanager.com/ for any additional comments that were saved as attachments.<br><br>In addition, please register any new software application in the SciCrunch.org database to receive a RRID (Research Resource Identification Initiative ID) number, and include this in your manuscript. This will facilitate tracking, reproducibility and re-use of your tool.<br><br>We have registered the project to SciCrunch.org and have added the ID to the Availability section.<br><br>Once you have made the necessary corrections, please submit a revised manuscript online at:<br><br>https://giga.editorialmanager.com/<br><br>If you have forgotten your username or password please use the "Send Login Details" link to get your login information. For security reasons, your password will be reset.<br><br>Please include a point-by-point within the 'Response to Reviewers' box in the submission system. Please ensure you describe additional experiments that were

carried out and include a detailed rebuttal of any criticisms or requested revisions that you disagreed with. Please also ensure that your revised manuscript conforms to the journal style, which can be found in the Instructions for Authors on the journal homepage.

The manuscript has been formatted according to the guidelines.

The due date for submitting the revised version of your article is 24 Dec 2018.

We look forward to receiving your revised manuscript soon.

Best wishes,

Nicole Nogoy, Ph.D
GigaScience
www.gigasciencejournal.com

Reviewer reports:
Reviewer #1: Review for PhenoMeNal: Processing and analysis of Metabolomics data in the Cloud

The authors have put together an impressive smorgasbord of software to allow for the data processing of multiple types of metabolomics datasets and continue on with post-processing. Wrapping the Galaxy software into a software-as-a-service system while also integrating other software that may not have been previously integrated into Galaxy. The authors seem to have gone to great lengths to consider open standards and have contacted many universities and institutes.

After reading the notes to authors and reviewers' guidelines it is still difficult to tell if the journal is expecting this type of manuscript. Additionally, due to this being published online I'll use first person.

The authors would like to thank reviewer 1 for the very helpful and valuable comments. We have revised the manuscript according to the comments. The manuscript is intended to be published as a Technical Note in GigaScience. We have formatted the manuscript according to the guidelines appropriate for this publication format.

In general, the manuscript in its current form reads more as a detailed documentation for developers, describing the underlying system. The manuscript is a bit strange in this way that it is presenting a heavy bioinformatic tool with details about company connections and European data regulations that are not often seen in informatic papers.

PhenoMeNal is a comprehensive project with participation of over 50 scientists from different research areas. Thus, PhenoMeNal includes the entire implementation workflow including the technical implementation, reproducibility, sustainability, regulations and ethics. We have revised the manuscript in such a way that also technically/informatically less experienced users understand it. To this end, we have removed very technical parts and added links to documentation in our wiki instead and also moved some informatic parts to the Supplemental.

There is a noticeable lack of comparison against other systems such as MetaboAnalyst, XCMS Online, Galaxy and other cloud-based metabolomics tools.

We have added a comparison to other tools. See our response above.

I would encourage the authors to have a distinct sentence or two saying why the manuscript is novel or why I should use it. I'm very sure that if published it will receive many citations.

The novelty of the project is now specified more clearly in the abstract and throughout the manuscript.

As someone who is already generally familiar with a lot of the discussed underlying

technologies it is a difficult read. I would not expect a non-informatic scientist to be able to understand the paper on their initial read. Again, to reiterate the manuscript needs to state why it is publishable.

We have revised the manuscript to be better understandable by scientists who are not bioinformaticians and also removed or relocated very technical parts. However, a certain level of informatic terminology is needed (i.e., discussing the underlying cloud technologies) to meet the requirements of GigaScience.

The abstract findings section is more of methods than what was discovered/found and conclusion does not state why PhenoMeNal is unique in to the aforementioned cloud systems.

The abstract has been rewritten to emphasize the uniqueness of PhenoMeNal.

Major:
1.The authors need to show why the manuscript is novel or what the system brings to the field. There is some attempt to do this via the 2 and ½ page table of programs that can be used however, a more direct comment on this would be very helpful.

The table containing the list of software tools has been moved to the Supplemental as it does not provide key information for the main text of the manuscript. The manuscript has been rewritten to show the uniqueness of PhenoMeNal (see response above).

2.Who is in charge of security checks on all open source apps into phenomenal? As was recently shown with python-pip unless someone is checking each and every app open source software can leak security.

In PhenoMeNal the tool developers and the release manager are in charge of security. They are automatically notified by GitHub on security issues. When security issues are reported, they trigger a new build in our CI system Jenkins and containers are built that contain the latest security patches and also include the latest stable versions from python-pip as dependencies. If security requires explicitly to install new or updated versions, the versions can be adapted in the Dockerfile. A concise version has been added in the security paragraph.

3.Figure 1 for the "today" seems to be very inaccurate again please cite and compare to other preexisting online cloud-based systems.

We have updated Figure 1.

4.What is the phenomal Cloud? How many cores can I allocate to this? How much data can I upload? This isn't discussed much in the documentation - do the authors not want people to use this ?

We are not sure what you mean…. We have specified the nature of PhenoMeNal and compared it to similar solutions in the Findings section. We further pointed out that limits on data storage and cpu cores really depends on the environment PhenoMeNal was deployed on and the parameters that were chosen.

5.The review suggests that figure 2, rather than a screen shot could demonstrate a workflow for the scientific workflow section.

Figure 2 has been redesigned as suggested showing 4 screenshots how to set up a PhenoMeNal e-infrastructure.

6.Reproducibility section a book is cited but a short description of what framework is used here would be nice as the book is rather long and not freely available.

The reference has been updated with an appropriate paper.

7.I noticed that the paper was supported by a European grant named phenomenal and it makes me wonder how long this grant will continue to get funded. I ask only because of the sustainability section. With such a complex system people need to be dedicated

Powered by Editorial Manager® and ProduXion Manager® from Aries Systems Corporation

to work on this. Many open source projects have become rust-ware, open source does not promise sustainability, simple-ness does. This software contains 9 programming languages and up to 6 platform dependencies.

The European Metabolomics Infrastructure Foundation was recently established through PhenoMeNal project members, that will do maintenance tasks on the developed infrastructure on a best-effort basis. The physical cloud infrastructure required to run PhenoMeNal is independently operated by third parties, including Amazon or Google, or scientific cloud installations like de.NBI or EOSC.

8.Where does the continuous integration happen? Again, this is import for the sustainability!

The Continuous Integration (CI) strategy is implemented in Jenkins-CI. We have added instructions in the Reproducibility section in Methods and linked from the Findings section to make the process more transparent.

9.NeIC-Tryggve2 - a short description of what this is and why it matters to the reader. Google brings up 5 listings for this so very few people probably know about it.

As Tryggve has started as an individual project, we have removed the slightly misleading reference from the manuscript.

10.Methods section is again very informatic heavy. Most scientist will not understand this please make this clearer and help the reader to understand why this is needed.

The methods section has mostly been rewritten for clarity and purpose. Specific informatic topics have been removed to improve the readability so that scientists from other fields do understand the section better.

11.In the scientific workflows the authors add clarity that PhenoMeNal is Galaxy, encapsulated. What does PhenoMeNal do that helps me run Galaxy. I do not feel this has be made clear.

This must be a misunderstanding. In PhenoMeNal, a specific metabolomics “flavour” of Galaxy can be deployed alongside other workflow management systems. The text in the manuscript has been rewritten to make it more concise and understandable.

12.Figure 6 does not add to the understanding of the manuscript. I understand this is digital and colour images are not costly to print however, figures should add content and help the reader to understand.

Figure 6 has been removed as suggested.

13.The manuscript cites that data was used however, I did not see any discussion about data and or processing of that data.

We have added a clarification to the supporting data section.

Minor:
1.I'm unaware of any dataset public or private that are terabytes in size. Many projects with multiple parts including transcriptomics, proteomics, histopathology and others can well exceed the terabytes size but normally it's hundreds of gigabytes. The cited paper talks about file sizes but does not mention datasets. Please find an additional citation if your saying this is in terms of epidemiological studies where there are 1000s of samples.

Phenome Centres process many thousands of metabolite profiles each year. References have been added and the relevant text has been rewritten. Multiple authors are also involved with a large-scale study (which is not published so far) in the field eco-metabolomics that has acquired over 1000 profiles.

2.The authors spend a lot of time talking about how to setup the system on amazon or

Powered by Editorial Manager® and ProduXion Manager® from Aries Systems Corporation

	google both of which can be pricy for academic users. They suggest openstack as a local based alternative. However, many institutes/universities (US based at least) do not run openstack. For an end user this is a lot of configuration to do. What about baremetal, HyperV etc…<br><br>The web-based portal supports deployments to AWS, GCE and OpenStack. From the command line, we also support Microsoft Azure, EOSC and bare metal installations. We have added links to our wiki pages which provides step-by-step instructions.<br><br>3.A description of what Datacloud and ECI bring to the project and why they are relevant. Many readers may not know<br><br>It is beyond the scope of the manuscript to describe these initiatives. We have added qualified references and URLs.<br><br>4.The authors cite the recently gone into effect GDPR. This is under the security section and I wonder how this is possible since patients will not know about this system and the metabolomics personal are a rather long way down the line from where the request will happen. Apologizes if I've not fully understood the GDPR.<br><br>In PhenoMeNal, GDPR is basically relevant with regard to patient consent. As this is just one minor aspect, explanation of GDPR has been shortened.<br><br>5.Table 1 could be in the supplementary. I'm not sure that it adds to the manuscript.<br><br>Table 1 has been relocated to the Supplement.<br><br>6.I would encourage the use of page numbers<br><br>Page numbers have been added to the manuscript.<br><br><br>Reviewer #2:<br>The authors have presented an exhaustive system that I believe would benefit the Metabolomics community vastly. I am glad to see that PhenoMeNal has taken into consideration the aspects of data openness, data standardisation and security whilst building this system. There are no improvements that I can think of from either a software engineering perspective or from the breadth of usability. I agree that PhenoMeNal is indeed a keystone solution and am looking forward to using it.<br><br>The authors would like to thank reviewer 2 for his/her positive feedback.
**Additional Information:**	
**Question**	**Response**
Are you submitting this manuscript to a special series or article collection?	No
**Experimental design and statistics**<br><br>Full details of the experimental design and statistical methods used should be given in the Methods section, as detailed in our Minimum Standards Reporting Checklist. Information essential to interpreting the data presented should be made available in the figure legends.<br><br>Have you included all the information	Yes

Powered by Editorial Manager® and ProduXion Manager® from Aries Systems Corporation

requested in your manuscript?	
**Resources**<br><br>A description of all resources used, including antibodies, cell lines, animals and software tools, with enough information to allow them to be uniquely identified, should be included in the Methods section. Authors are strongly encouraged to cite Research Resource Identifiers (RRIDs) for antibodies, model organisms and tools, where possible.<br><br>Have you included the information requested as detailed in our Minimum Standards Reporting Checklist?	Yes
**Availability of data and materials**<br><br>All datasets and code on which the conclusions of the paper rely must be either included in your submission or deposited in publicly available repositories (where available and ethically appropriate), referencing such data using a unique identifier in the references and in the “Availability of Data and Materials” section of your manuscript.<br><br>Have you have met the above requirement as detailed in our Minimum Standards Reporting Checklist?	Yes

Manuscript with DOI
Click here to view linked References
Click here to access/download;Manuscript;_Technical_Note_GigaScience_P

PhenoMeNal: Processing and analysis of Metabolomics data in the Cloud

Kristian Peters*[1], James Bradbury*[2], Sven Bergmann[3,4], Marco Capuccini[5,6], Marta Cascante[7], Pedro de Atauri[8], Timothy M D Ebbels[9], Carles Foguet[8], Robert Glen[9,10], Alejandra Gonzalez-Beltran[11], Ulrich L. Günther[12], Evangelos Handakas[9], Thomas Hankemeier[13,14], Kenneth Haug[15], Stephanie Herman[6,16], Petr Holub[17], Massimiliano Izzo[11], Daniel Jacob[18], David Johnson[11,12], Fabien Jourdan[20], Namrata Kale[16], Ibrahim Karaman[21], Bita Khalili[3,4], Payam Emami Khonsari[17], Kim Kultima[17], Samuel Lampa[6], Anders Larsson[22], Christian Ludwig[23], Pablo Moreno[16], Steffen Neumann[1,24], Jon Ander Novella[22], Claire O'Donovan[16], Jake TM Pearce[9], Alina Peluso[9], Marco Enrico Piras[25], Luca Pireddu[25], Michelle AC Reed[13], Philippe Rocca-Serra[11], Pierrick Roger[26], Antonio Rosato[27], Rico Rueedi[3,4], Christoph Ruttkies[1], Noureddin Sadawi[9], Reza M Salek[16], Susanna-Assunta Sansone[11], Vitaly Selivanov[8], Ola Spjuth[6], Daniel Schober[1], Etienne A. Thévenot[26], Mattia Tomasoni[3,4], Merlijn van Rijswijk[28,29], Michael van Vliet[30], Mark R Viant[2], Ralf J. M. Weber[2], Gianluigi Zanetti[25], Christoph Steinbeck*[31]

* - corresponding authors
1 - Leibniz Institute of Plant Biochemistry, Stress and Developmental Biology, Weinberg 3, 06120 Halle (Saale), Germany
2 - School of Biosciences, University of Birmingham, Edgbaston, Birmingham, B15 2TT, United Kingdom
3 - Department of Computational Biology, University of Lausanne, Lausanne, Switzerland
4 - Swiss Institute of Bioinformatics, Lausanne, Switzerland
5 - Division of Scientific Computing, Department of Information Technology, Uppsala University, Sweden
6 - Department of Pharmaceutical Biosciences, Uppsala University, Box 591, 751 24 Uppsala, Sweden
7 - Department of Biochemistry and Molecular Biomedicine, Universitat de Barcelona; Centro de Investigación Biomédica en Red de Enfermedades Hepáticas y Digestivas
8 - Department of Biochemistry and Molecular Biomedicine, Universitat de Barcelona; Centro de Investigación Biomédica en Red de Enfermedades Hepáticas y Digestivas (CIBEREHD), Instituto de Salud Carlos III (ISCIII), Spain
9 - Department of Surgery & Cancer, Imperial College London, South Kensington, London, SW7 2AZ, United Kingdom
10 - Centre for Molecular Informatics, Department of Chemistry, University of Cambridge, Lensfield Road, Cambridge, CB21EW, United Kingdom
11 - Oxford e-Research Centre, Department of Engineering Science, University of Oxford, 7 Keble Road, OX1 3QG, Oxford, United Kingdom.
12 - Department of Informatics and Media, Uppsala University, Box 513, 751 20 Uppsala, Sweden
13 - Institute of Cancer and Genomic Sciences, University of Birmingham, Edgbaston, Birmingham, B15 2TT, United Kingdom
14 - Netherlands Metabolomics Center, Leiden, 2333 CC, Netherlands
15 - Division of Systems Biomedicine and Pharmacology, Leiden Academic Centre for Drug

Formatted: Position Horizontal: Center, Relative to: Margin, Vertical: 0", Relative to: Paragraph, Wrap Around

[16] - European Molecular Biology Laboratory, European Bioinformatics Institute (EMBL-EBI), Wellcome Genome Campus, Hinxton, Cambridge CB10 1SD, United Kingdom
[17] - Department of Medical Sciences, Clinical Chemistry, Uppsala University, 751 85 Uppsala, Sweden
[18] - BBMRI-ERIC, Graz, Austria
[19] - INRA, University of Bordeaux, Plateforme Métabolome Bordeaux-MetaboHUB, 33140 Villenave d'Ornon, France
[20] - INRA - French National Institute for Agricultural Research, UMR1331, Toxalim, Research Centre in Food Toxicology, Toulouse, France
[21] - Department of Epidemiology and Biostatistics, School of Public Health, Imperial College London, St. Mary's Campus, Norfolk Place, W2 1PG, London, United Kingdom
[22] - National Bioinformatics Infrastructure Sweden, Uppsala University, Uppsala, Sweden Department of Pharmaceutical Biosciences, Uppsala University, Uppsala, Sweden
[23] - Institute of Metabolism and Systems Research (IMSR), University of Birmingham, Edgbaston, Birmingham, B15 2TT, United Kingdom
[24] - German Centre for Integrative Biodiversity Research (iDiv) Halle-Jena-Leipzig, Deutscher Platz 5e, 04103 Leipzig, Germany
[25] - Distributed Computing Group, CRS4, Pula, Italy
[26] - CEA, LIST, Laboratory for Data Analysis and Systems' Intelligence, MetaboHUB, Gif-Sur-Yvette F-91191, France
[27] - Magnetic Resonance Center (CERM) and Department of Chemistry, University of Florence and CIRMMP, 50019 Sesto Fiorentino, Florence, Italy
[28] - ELIXIR-NL, Dutch Techcentre for Life Sciences, Utrecht, 3503 RM, Netherlands
[29] - Netherlands Metabolomics Center, Leiden, 2333 CC, The Netherlands
[30] - Division of Systems Biomedicine and Pharmacology, Leiden Academic Centre for Drug Research (LACDR), Leiden University, Leiden, 2333 CC, The Netherlands
[31] - Cheminformatics and Computational Metabolomics, Institute for Analytical Chemistry, Lessingstr. 8, 07743 Jena, Germany

ORCIDs:

Kristian Peters kpeters@ipb-halle.de 0000-0002-4321-0257
James Bradbury j.bradbury@bham.ac.uk
Sven Bergmann sven.bergmann@unil.ch
Marco Capuccini marco.capuccini@it.uu.se 0000-0002-4851-759X
Marta Cascante martacascante@ub.edu 0000-0002-2062-4633
Pedro de Atauri pde_atauri@ub.edu 0000-0002-7754-7851
Tim Ebbels timebbels@gmail.com
Carles Foguet cfoguet@ub.edu 0000-0001-8494-9595
Robert Glen r.glen@imperial.ac.uk
Alejandra N. Gonzalez-Beltran alejandra.gonzalezbeltran@oerc.ox.ac.uk 0000-0003-3499-8262
Ulrich Guenther u.l.gunther@bham.ac.uk
Evangelos Handakas e.chandakas@imperial.ac.uk
Thomas Hankemeier hankemeier@lacdr.leidenuniv.nl 0000-0001-7871-2073
Stephanie Herman stephanie.herman@medsci.uu.se 0000-0001-9382-3273
Kenneth Haug kenneth@ebi.ac.uk 0000-0003-3168-4145
Petr Holub petr.holub@bbmri-eric.eu 0000-0002-5358-616X
Massimiliano Izzo massimiliano.izzo@oerc.ox.ac.uk 0000-0002-8100-6142

Formatted: Position Horizontal: Center, Relative to: Margin, Vertical: 0", Relative to: Paragraph, Wrap Around

Daniel Jacob djacob@u-bordeaux.fr 0000-0002-6687-7169
David Johnson david.johnson@im.uu.se 0000-0002-2323-6847
Fabien Jourdan fabienjourdan@gmail.com
Namrata Kale namrat@ebi.ac.uk 0000-0002-4255-8104
Ibrahim Karaman i.karaman@imperial.ac.uk 0000-0001-9341-8155
Bita Khalili bita.khalili@unil.ch
Payam Emami Khoonsari payam.emami@medsci.uu.se 0000-0002-4137-5517
Kim Kultima kim.kultima@medsci.uu.se 0000-0002-0680-1410
Samuel Lampa samuel.lampa@farmbio.uu.se 0000-0001-6740-9212
Anders Larsson anders.larsson@icm.uu.se 0000-0002-2096-8102
Christian Ludwig c.ludwig@bham.ac.uk
Pablo Moreno pmoreno@ebi.ac.uk 0000-0002-9856-1679
Steffen Neumann sneumann@ipb-halle.de 0000-0002-7899-7192
Jon Ander Novella jon.novella@farmbio.uu.se 0000-0002-2187-5426
Claire O'Donovan odonovan@ebi.ac.uk 0000-0001-8051-7429
Jake TM Pearce jake.pearce@gmail.com 0000-0003-4637-3171
Alina Peluso a.peluso@imperial.ac.uk 0000-0003-2895-0406
Luca Pireddu luca.pireddu@crs4.it 0000-0002-4663-5613
Marco Enrico Piras marcoenrico.piras@crs4.it 0000-0002-5207-0030
Michelle AC Reed m.thompson@bham.ac.uk 0000-0002-0667-4490
Philippe Rocca-Serra philippe.rocca-serra@oerc.ox.ac.uk 0000-0001-9853-5668
Pierrick Roger pierrick.roger@gmail.com
Antonio Rosato rosato@cerm.unifi.it 0000-0001-6172-0368
Rico Rueedi rico.rueedi@unil.ch 0000-0002-6713-2214
Christoph Ruttkies ruttkies@web.de 0000-0002-8621-8689
Noureddin Sadawi noureddin.sadawi@gmail.com 0000-0002-2195-8264
Reza M Salek reza.salek@ebi.ac.uk 0000-0001-8604-1732
Susanna-Assunta Sansone susanna-assunta.sansone@oerc.ox.ac.uk 0000-0001-5306-5690
Vitaly Selivanov seliv55@gmail.com 0000-0002-7937-9249
Ola Spjuth ola.spjuth@farmbio.uu.se 0000-0002-8083-2864
Daniel Schober DanielSawstMsi@gmail.com 0000-0001-8014-6648
Etienne A. Thévenot etienne.thevenot@cea.fr 0000-0003-1019-4577
Mattia Tomasoni mattia.tomasoni@unil.ch
Merlijn van Rijswijk merlijn.van.rijswijk@dtls.nl 0000-0002-1067-7766
Michael van Vliet m.s.vanvliet@lacdr.leidenuniv.nl 0000-0002-5034-5766
Mark Viant m.viant@bham.ac.uk 0000-0001-5898-4119
Ralf Weber r.j.weber@bham.ac.uk
Gianluigi Zanetti gianluigi.zanetti@crs4.it 0000-0003-1683-7350
Christoph Steinbeck christoph.steinbeck@uni-jena.de 0000-0001-6966-0814

Formatted: Position Horizontal: Center, Relative to: Margin, Vertical: 0", Relative to: Paragraph, Wrap Around

Abstract

Background: Metabolomics is the comprehensive study of a multitude of small molecules to gain insight into an organism's metabolism. The research field is dynamic and expanding with applications across biomedical, biotechnological and many other applied biological domains. Its computationally-intensive nature has driven requirements for open data formats, data repositories and data analysis tools. However, the rapid progress has resulted in a mosaic of independent – and sometimes incompatible – analysis methods that are difficult to connect into a useful and complete data analysis solution.
Findings: PhenoMeNal (Phenome and Metabolome aNalysis) is an advanced and complete solution to set up Infrastructure-as-a-Service (IaaS) that brings workflow-oriented, interoperable metabolomics data analysis platforms into the cloud. PhenoMeNal seamlessly integrates a wide array of existing open source tools which are tested and packaged as Docker containers through the project's continuous integration process and deployed based on a kubernetes orchestration framework. It also provides a number of standardized, automated and published analysis workflows in the user interfaces Galaxy, Jupyter, Luigi and Pachyderm.
Conclusions: PhenoMeNal constitutes a keystone solution in cloud e-infrastructures available for metabolomics. PhenoMeNal is a unique and complete solution for setting up cloud e-infrastructures through easy-to-use web interfaces that can be scaled to any custom public and private cloud environment. By harmonizing and automating software installation and configuration and through ready-to-use scientific workflow user interfaces, PhenoMeNal has succeeded in providing scientists with workflow-driven, reproducible and shareable metabolomics data analysis platforms which are interfaced through standard data formats, representative datasets, versioned, and have been tested for reproducibility and interoperability. The elastic implementation of PhenoMeNal further allows easy adaptation of the infrastructure to other application areas and 'omics research domains.

Keywords

Metabolomics, Data Analysis, e-infrastructures, NMR, Mass Spectrometry, Computational Workflows, Galaxy, Cloud Computing, Standardization, Statistics

Formatted: Position Horizontal: Center, Relative to: Margin, Vertical: 0", Relative to: Paragraph, Wrap Around

Findings

Background

The field of metabolomics has seen remarkable progress over the last decade and has enabled fascinating discoveries in many different research areas. Metabolomics is the study of small molecules in organisms which can reveal detailed insights into metabolic biochemistry, e.g. changes in concentrations of specific molecules, metabolic fluxes between cells or compartments, identification of molecules that are involved in the pathogenesis of a disease, the study of the biochemical phenotype of animals, plants and even soil microorganisms [1–3].

The principal metabolomics technologies of mass spectrometry (MS) and nuclear magnetic resonance spectroscopy (NMR) typically generate large data sets that require computationally intensive analyses [4]. Biomedical investigations can involve large cohorts with many thousands of metabolite profiles and can produce hundreds of gigabytes of data [5–8]. With such large data sets, processing becomes impracticable and unmanageable on commodity hardware. Cloud computing can offer a solution by enabling the outsourcing of calculations from local workstations to scalable cloud data centers, with the possibility to allocate thousands of CPU cores simultaneously. Furthermore, cloud computing allows for resources to be instantiated on-demand (CPUs, RAM, network, storage) and access to computational tools in the form of microservices that can dynamically grow or shrink.

MS and NMR data processing usually involve selection of parameters (which are often specific to the analytical instrumentation), algorithmic peak detection, peak alignment and grouping, annotation of putative compounds and extensive statistical analyses [9,10]. Many open source tools have been developed that address these different steps in data processing and analysis. These tools, however, usually come with their own software dependencies, resource requirements and scripting languages. As a consequence, configuring and running them is often complicated, especially for researchers who are untrained in computer science [4]. Furthermore, many tools require users to input parameters that can significantly affect results and performance, and reporting of these parameters is not always clear [11].

In the last five years, a number of infrastructures and integration efforts were initiated, including metabolomics data repositories with a global scope [6,12], platforms for reproducible workflow analysis [13,14], as well as initiatives to integrate and coordinate data standards [15]. Simultaneously, multiple networks of service centers such as the international Phenome Centers [16] and MetaboHub [17] have formed with the goal to facilitate the acquisition, processing and analysis of metabolomics data [6–8] at ever increasing scales.

Currently, several web-based metabolomics data processing platforms are available. XCMSOnline provides a platform based on XCMS for downstream data analysis, visualization, data sharing and access to Metlin to facilitate metabolite identification and pathway analysis [18]. MetaboAnalyst presents a wide variety of data processing and analysis tools including statistical analysis, time-series analysis, functional analysis and pathway analysis [19]. Workflow4Metabolomics is based on Galaxy and provides various metabolomics processing workflows, including NMR [13,20]. These common tools for analysing metabolomics data provide web-based graphical user interfaces (GUIs) with different functionality.

Formatted: Position Horizontal: Center, Relative to: Margin, Vertical: 0", Relative to: Paragraph, Wrap Around

Here we present PhenoMeNal (Phenome and Metabolome aNalysis), a unique, easy-to-use, complete, robust and performant cloud e-infrastructure that provides a large suite of standardized and interoperable metabolomics data processing tools as a complete data analysis solution. In contrast to current metabolomics processing platforms, PhenoMeNal provides Infrastructure-as-a-Service (IaaS) and seamlessly integrates a wide array of existing open source tools.

A major advantage over other platforms is that PhenoMeNal allows to instantiate many different services in the cloud and provides a number of standardized, automated and published analysis workflows in the user interfaces Galaxy, Jupyter, Luigi and Pachyderm (Fig. 1). Moreover, the PhenoMeNal e-infrastructure can be easily deployed onto public and private cloud environments and can be configured elastically to fit into any cloud-based environment, and thus enabling scalable and cost-effective high-performance metabolomics data analysis in a way that hides the technical complexity from the user. PhenoMeNal further facilitates reproducible analyses through automated, sharable and citable workflows.

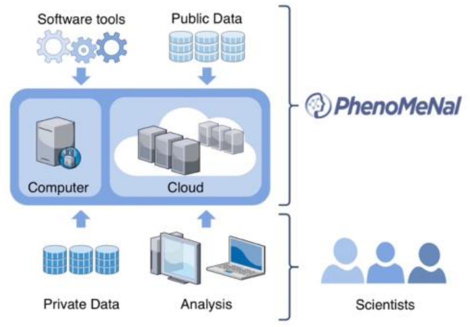

Figure 1: Conceptual design of the PhenoMeNal cloud e-infrastructure, which brings compute to the data for any large number of data scientists.

Overview

The features of the PhenoMeNal e-infrastructure are encapsulated as a Cloud Research Environment (CRE). The PhenoMeNal CRE can be instantiated on major commercial public cloud providers, including Amazon Web Services (AWS) and Google Cloud Platform (GCE), as well as OpenStack-based private clouds and in custom environments. Technical complexity is hidden from the users, simplifying setting up the cloud infrastructure for administrators (Fig. 2).

From a web-based portal, users can deploy the CRE, which includes several web services and software tools (Fig. 2). Data can be processed directly in the e-infrastructure without the need to install additional software. Scientific workflows can be executed via user friendly web-based platforms such as Galaxy, as well as programmatic interfaces and notebooks. Each service has been supplied with a rich source of documentation and training material to assist researchers.

Formatted: Position Horizontal: Center, Relative to: Margin, Vertical: 0", Relative to: Paragraph, Wrap Around

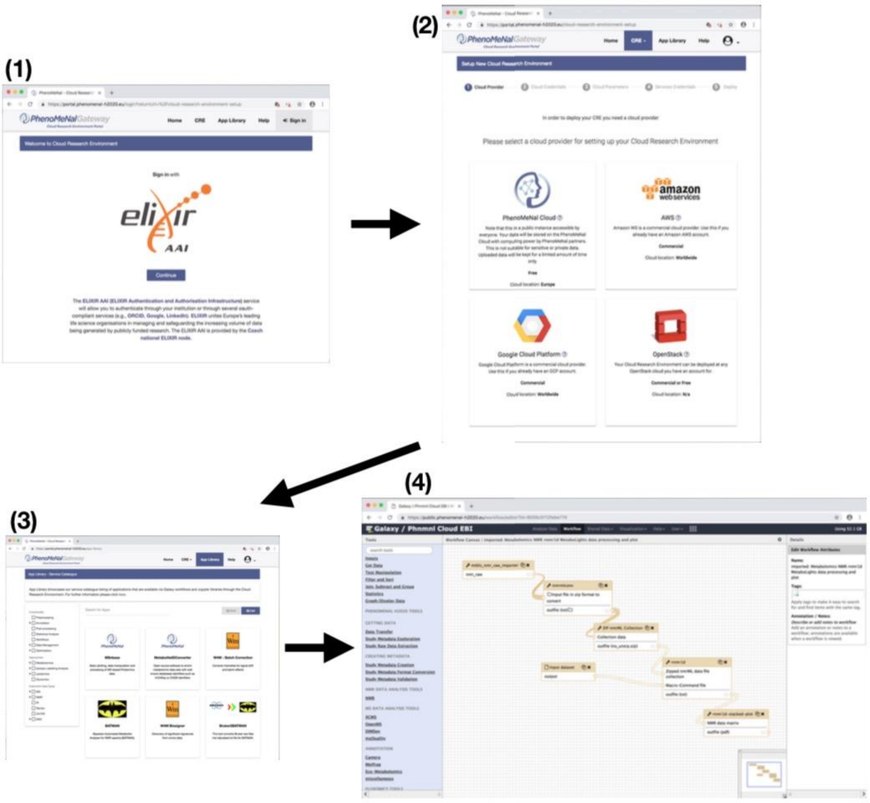

Figure 2: Screenshots of creating and using the PhenoMeNal cloud e-infrastructure. First, login with ELIXIR to the Cloud Research Environment (CRE) portal. Second, selecting a public or private cloud provider. After entering cloud credentials and setting up parameters in the dedicated portal, the deployment of the PhenoMeNal e-infrastructure into the cloud environment can be made. Third, in the PhenoMeNal Portal App Library there are several services ready to be deployed and used in the set-up infrastructure. Fourth, dedicated web services such as Galaxy are readily available in the cloud e-infrastructure. All steps can be operated from an easy-to-use web interface which is accessible from any standard web browser.

The PhenoMeNal Portal

The PhenoMeNal Portal [21], allows users to deploy, manage and delete PhenoMeNal CREs simply through a web interface. Deployments to major commercial cloud platforms (AWS and GCP) as well as OpenStack, an open source cloud platform, can be made using an easy to follow wizard (Fig. 2). OpenStack deployments can be deployed behind clinical firewalls, which is especially pertinent when dealing with sensitive (i.e. patient) data.

Formatted: Position Horizontal: Center, Relative to: Margin, Vertical: 0", Relative to: Paragraph, Wrap Around

The PhenoMeNal public instance allows users to test-run a CRE without the need to deploy on a cloud platform. It can be deployed and accessed through the portal. Once credentials for users have been generated, analyses can be run through a Galaxy instance containing the tools and workflows present in any deployed CRE. The portal also includes user and developer documentation, workflow tutorials and links to training videos.

Scientific workflows

A scientific workflow is a set of computational steps that are carried out to process and analyze data [22]. Usually, a workflow is comprised of several linked software tools that are each executed during a particular step of the workflow. In order to manage and automate scientific workflows, in PhenoMeNal the well-established dedicated workflow management system Galaxy can be deployed, which presents the user with an easy to use graphical user interface as well as providing a programmatic interface [20,23]. Galaxy facilitates collaborative exchange, reproducibility and traceability of data analysis by enabling users to share entire workflows and analysis histories [24]. In addition to Galaxy, programmatic executable notebooks (Jupyter) and the workflow tools exposed as programmatic interfaces Luigi and Pachyderm are also supported [25].

In order to cover typical use cases in metabolomics and to illustrate the usage and applicability of given analytical pipelines and software tools, five representative scientific workflows are available in the PhenoMeNal Galaxy (Table 1), each having different computational demands and purposes. More than 250 individual modules have been integrated in Galaxy (see section scientific workflows in Methods).

Software tools

The Portal App Library [26], shows the software tools packaged in PhenoMeNal that are available through the CRE deployment (Fig. 2). The range of software tools available cover several metabolomics domains, making PhenoMeNal relevant for use in a wide range of data analysis scenarios. The domains covered include clinical metabolomics, plant metabolomics, fluxomics and eco-metabolomics. Data from both targeted and untargeted analysis can be analyzed for metabolite profiling and fingerprinting approaches [1,2]. NMR and MS (LC/MS, GC/MS, DIMS) data can be processed.

PhenoMeNal also provides tools for data management (e.g. via the ISA format and API), metabolite feature detection (e.g. XCMS, CAMERA, nmrProcFlow), metabolite identification (MetFrag, BATMAN, MetaboMatching) and (bio)statistics (e.g. univariate, multivariate and power analyses) (Supplemental Table 1). Tools can be filtered for functionality, approaches and instrument (data) types to readily find the most appropriate software tools. Some tools that implement specific functionality (e.g. Rnmr1D which performs baseline correction of NMR spectra as part of nmrProcFlow) are available through dedicated Galaxy modules or through software containers (Supplemental Table 1).

Study Design

PhenoMeNal was designed to use standardized protocols, software tools and comply with state-of-the-art dedicated specifications and data formats across the entire project. Development was geared towards implementation of open standards for tracking provenance of both data and metadata generated by clinical phenotyping projects. In PhenoMeNal, the ISA-model and specifications were implemented using the ISA format to

Formatted: Position Horizontal: Center, Relative to: Margin, Vertical: 0", Relative to: Paragraph, Wrap Around

generate, annotate, validate and deposit experimental metadata information of data sets and studies to public repositories such as MetaboLights [27,28]. ISA-based metadata tracking is used for the different analysis pipelines which are specific to the distinct metabolomics domains. PhenoMeNal reached native support for the ISA format by developing a dedicated Galaxy composite data type. Such component affords direct recognition of the ISA format by the Galaxy environment, thus ensuring seamless integration with downstream workflow component.

Data deposition

PhenoMeNal encourages the metabolomics data repository MetaboLights as a primary source of data deposition [29]. Private and public data sets are supported, as well as download and upload to MetaboLights. If the storage in a data repository such as MetaboLights is not possible, data can be stored locally or in the cloud e-infrastructure. Access to the data is strictly controlled and secured. To support data deposition, ISA-based Galaxy modules are available allowing to publish and disseminate scientific results in standard compliant ways.

Reproducibility

One of the challenges of cloud computing is that analyses need to be run continuously and successfully in different environments [30]. Specifically, it has to be ensured that, given the same input, workflows and tools produce identical results regardless of the underlying environment [4,30]. When these requirements are fulfilled, end users can be confident that their data will be analyzed correctly. PhenoMeNal has implemented three major testing strategies to ensure technical reproducibility using a continuous integration framework [31]. Tests were implemented for the infrastructure components, individual software containers and for data involved in computational workflows.

Sustainability

PhenoMeNal is part of a number of initiatives (BioMedBridges, COSMOS and ELIXIR) to foster the role of metabolomics and to harmonize experimental data and metadata usage [15,32]. Collaborations were established with EGI [33] and Indigo Datacloud [34] infrastructure providers and initiatives [35,36], to ensure that PhenoMeNal uses technologies that are well-supported and assure their widespread usage, continuity and further development. For example, the development of KubeNow and contributions to the Galaxy and Workflow4Metabolomics community are essential for PhenoMeNal [37]. Core development will continue on GitHub and is fostered by collaborations with tool developers.

Dependencies on specific technologies and frameworks were avoided by focusing on open standards such as ISA-Tab / ISA-JSON, mzML and nmrML and widely accepted software [38]. By being able to deploy PhenoMeNal on multiple types of cloud environments, lock-in to specific computing resource providers are avoided. PhenoMeNal implemented continuous integration and delivery, validated by extensive testing and with clear maintenance responsibilities (see sections in Methods).

Formatted: Position Horizontal: Center, Relative to: Margin, Vertical: 0", Relative to: Paragraph, Wrap Around

Privacy and security

With human or animal material the collection, storage and analysis of metabolomics data introduce a number of constraints due to Ethical, Legal and Social Implications (ELSI) [39]. In particular, data initially derived from human clinical studies may be identifiable and will require consent for use, usually for a defined objective such as diagnosis, or be related to a particular disease study. Where data is identifiable or pseudonymized, users can deploy PhenoMeNal on local secure resources, thus avoiding the export of data. In this scenario, access to the e-infrastructure should be strictly controlled through local access and authorization. It is recommended that clinical data is fully anonymized before analysis in PhenoMeNal [39,40].

The PhenoMeNal portal provides substantial guidance to enable users to comply with ELSI and General Data Protection Regulation (GDPR) requirements. Users must register in order to use the individual parts of the e-infrastructure. PhenoMeNal was implemented to use secured and encrypted transport and network communications.

Documentation and Training materials

Extensive user documentation and tutorials are provided via the PhenoMeNal Wiki page [41]. The Wiki includes detailed developer resources including information about the PhenoMeNal release schedule, guidelines for tool, workflow and portal developers, continuous integration and testing. Further documentation is also provided detailing, creating and managing PhenoMeNal CREs, tutorials for the Galaxy modules and pre-configured workflows, as well as Galaxy tours that provide step by step guidance for inexperienced users.

Community engagement

The PhenoMeNal project is open source, and is hosted on GitHub [42]. Developers can contribute tools to PhenoMeNal and are encouraged to do so. To add a tool to PhenoMeNal, it must be containerized using Docker, and then integrated into the build process. Detailed documentation is available in the project's Wiki for developers who wish to add their tools to PhenoMeNal.

Collaborations with other projects have been actively encouraged during the development of PhenoMeNal, including Workflow4Metabolomics [13] and the developers of both nmrML and nmrProcFlow [43]. These collaborations are essential to foster greater standardization within PhenoMeNal and to increase compatibility with other metabolomics data processing infrastructures.

Availability

Information on how to access PhenoMeNal can be found at the project's website [44]. The GitHub repository hosts the source code of all development projects [42]. The project container-galaxy-k8s-runtime contains all of the developments regarding Galaxy. The Wiki containing documentation is also hosted on GitHub [41]. The PhenoMeNal Portal can be reached at [21]. The public instance of Galaxy is accessible at [45]. Source code and documentation are available under the terms of the Apache 2.0 license. Integrated open source projects are available under the respective licensing terms.

Formatted: Position Horizontal: Center, Relative to: Margin, Vertical: 0", Relative to: Paragraph, Wrap Around

Conclusions

PhenoMeNal has succeeded in increasing the robustness and coverage of representative metabolomics data processing in scientific cloud e-infrastructures. The presented cloud e-infrastructure covers a wide range of analysis pipelines including data generation and download, data pre- and post-processing, (bio)statistics and result deposition in data repositories. A large effort has been made to introduce lower level changes to cloud e-infrastructures (e.g. the cloud deployment software KubeNow) to meet the demands of the biomedical domain. Furthermore, Galaxy has been enriched with metabolomics data standards, in particular the ISA format for study metadata and mzML and nmrML for acquired data files, as well as support for Kubernetes. PhenoMeNal has fostered the visibility of new metabolomics tools and has enabled the development of more sophisticated data analysis workflows. Our efforts were also guided by feedback from real life test scenarios collected at workshops with users from the clinical domain.

PhenoMeNal constitutes a keystone solution in cloud platforms available for metabolomics data analysis. The platform was designed to deliver optimal performance and functionality for typical use cases in the metabolomics domain. While the needs of clinicians and researchers in the biomedical and biochemical domains have been targeted, PhenoMeNal is not limited to a specific domain as the cloud infrastructure, tools and workflows can be adapted to other use cases as demonstrated with the inclusion of the eco-metabolomics workflow. The technological advancements can be reused in other scientific cloud environments and could be integrated with solutions from other ‘omics domains in the future.

Formatted: Position Horizontal: Center, Relative to: Margin, Vertical: 0", Relative to: Paragraph, Wrap Around

Methods

Cloud e-infrastructure

The PhenoMeNal CRE is designed as a microservice architecture, with services being implemented as Virtual Machine Images (VMIs) and software containers. Containers are used to provision microservices for metabolomics data analysis tools and also long-running services such as workflow management systems. A container orchestrator runs containers on top of the scalable infrastructure. The orchestrator takes a group of machines that act as a distributed cluster and receives requests for tools as well as services executions. PhenoMeNal implements various layers to provision a container orchestrator on top of either bare metal hardware or Infrastructure-as-a-Service (IaaS) given by a cloud provider [46] (Supplemental Fig. 1).

During the setup process and while PhenoMeNal is deployed, data storage and cpu limits can be configured and dynamically scaled to fit any cloud environment. Deployments can be made to GCE, AWS and OpenStack-based private clouds from the PhenoMeNal portal. Deployments are also supported from the command line to Microsoft Azure [47], the European Science Cloud (EOSC) [48] and local servers (bare metal) [49], and we provide step-by-step instructions for these solutions.

PhenoMeNal provides IaaS for three different cloud environments:

1. “local cloud”: local workstations or bare metal clusters where data are not allowed to leave the facility.
2. “public cloud”: the flexible use of commercial cloud providers such as GCP and AWS.
3. “shared cloud”: using OpenStack - a free and open-source software platform for cloud computing, ideal for custom environments and research networks.

Software tools

The PhenoMeNal portal has an Application Library that allows users to deploy tools as microservices into the cloud infrastructure (Fig. 2, Supplemental Table 1). The portal is packaged into frontend and backend engines on top of Kubernetes.

Most software tools in PhenoMeNal are compiled from source code and use a variety of programming languages. Linux versions of software tools and user interfaces such as Galaxy are supported in dedicated encapsulated Docker containers which are implemented as minimum-sized microservices. PhenoMeNal currently hosts 100 such projects in its GitHub repository (https://github.com/phnmnl/?q=container) (Supplemental Table 1). Projects are indicated by the trailing `container-` name and include a ruleset to build and run the containerized tools, as well as data sets for testing and other necessary files.

PhenoMeNal provides tutorials for developers who want to integrate their tools into our e-infrastructure [50].

Scientific workflows

In PhenoMeNal, a number of options are available for running reproducible and standardized workflows (Table 1).

Formatted: Position Horizontal: Center, Relative to: Margin, Vertical: 0", Relative to: Paragraph, Wrap Around

Galaxy

The Galaxy workflow management system is widely regarded as one of the most popular scientific workflow platforms [20,51]. It provides a user-friendly web-based graphical user interface (GUI) to make it easy for the end-user to configure and run individual modules and entire workflows without programming experience. Command-line tools and scripts are encapsulated into modules that are launched via the web interface. Galaxy also supports more powerful features like programmatic access through a REST API and helper libraries to access the running instance of Galaxy [52].

PhenoMeNal has achieved to adapt Galaxy for use with a microservices based architecture [53]. To this end, modules are encapsulated into Docker containers that can be flexibly launched within the cloud e-infrastructure. Galaxy is available in all deployed PhenoMeNal CREs and contains more than 250 modules that have been implemented as part of PhenoMeNal.

Six representative metabolomics Galaxy workflows have been fully integrated into PhenoMeNal (Table 1) and more workflows (mzQuality, NMR-BATMAN) are available for testing.

Jupyter

Jupyter, which started its history as the IPython notebook, is the most popular among tools commonly referred to as executable notebooks or computational notebooks [54]. Jupyter lets users combine executable code with results from code executions such as text, tables and figures. Usually Jupyter notebooks are enriched with extended information that explain what the code does. As a result, they are often used for training material and for tutorials. Computational notebooks can also to some extent be used as a way to document code executions and to make executions more reproducible [55].

Luigi and Pachyderm

Luigi is a Python workflow programming library that was originally developed by the company Spotify. It manages pipelines of computations primarily on Big Data systems such as Hadoop and Apache Spark but also supports local execution [54,55]. Luigi is a very flexible library that facilitates building complex pipelines of batch jobs handling dependency resolution, workflow management and visualization.

Similarly, Pachyderm allows to process distributed data and to keep track of the data from every stage of the analysis pipeline [25]. With Pachyderm it is possible to track the provenance of results and to accurately reproduce scientific workflows. Luigi and Pachyderm are well suited for complex scientific tasks and are easy to use from the python-environment in Jupyter notebooks without additional integration tooling needed.

In PhenoMeNal, we have extended Galaxy, Jupyter, Luigi and Pachyderm in such a way that they can be orchestrated throughout the cloud infrastructure together with the data analysis tools themselves [53]. Six important metabolomics workflows have been fully integrated into PhenoMeNal (Table 1) and more (mzQuality, NMR-BATMAN) are available for testing (Fig. 6) [212].

Table 1: List of workflows which are representative for their respective metabolomics domains (Identification in NMR, Fluxomics, Annotation and identification in MS and eco-metabolomics).

Formatted: Position Horizontal: Center, Relative to: Margin, Vertical: 0", Relative to: Paragraph, Wrap Around

Workflow name	Description	References
1D NMR	Processes 1D NMR experiments from raw data to a data matrix required for visualisation and statistical analysis, building on nmrML and NMRProcFlow. The automatic workflow is based on the MTBLS1 data set, describing urinary changes in type 2 diabetes in humans.	[43,56,57]
Fluxomics	Quantifies steady state fluxes following ^{13}C Metabolic Flux Analysis. The workflow was first based on the analysis of the MTBLS412 data set with 13C tracer data of human umbilical vein endothelial cells (HUVEC) under hypoxia.	[58,59]
LC-MS/MS	Processes, quantifies and annotates/identifies features in mass spectra using MetFrag - a tool which annotates molecules from compound databases of tandem mass spectrometry (MS/MS) spectra. The workflow is based on MTBLS558.	[53,60,61]
Univariate and Multivariate Statistics	Applies univariate and multivariate statistical analysis, and illustrates how data sets may be explored enabling the identification of variables of interest and the construction of predictive models. The workflow is based on MTBLS404.	[13,62]
Eco-Metabolomics	Implementation of a resource demanding metabolomics use case in ecology, used in large field experiments to describe interactions between different species of organisms in remarkable detail. The workflow is based on MTBLS520.	[63]
ISA-Create-Validate-Upload	A workflow to create ISA compliant metadata files based on study design information, augmented with semantic markup as source, implementing UK Phenome center naming conventions. Following validation, the workflow also allows visualization of overall study design and deposition to EMBL-EBI	

Reproducibility

Three strategies are realized to ensure technical reproducibility. They are implemented in the continuous integration (CI) software development framework Jenkins [31] which is accessible at [64]. These strategies are implemented as tests in our Jenkins and a tutorial guide is available at [65].

- Infrastructure testing: Procedures were implemented to ensure that each individual component (e.g. the deployment process of software containers, resource management, APIs / ABIs) within the infrastructure is interacting correctly with the other components.
- Container testing: Verification that tools, which are packaged into software containers, build and run correctly in the infrastructure. Dependencies within one container and across several interdependent containers are tested.
- Data testing: The output of tools, which process demonstration data, is checked against a data set that is known to contain the expected result. This is being done for both individual tools and for several tools running in a workflow using the workflow testing tool for Galaxy called wft4galaxy [66].

Formatted: Position Horizontal: Center, Relative to: Margin, Vertical: 0", Relative to: Paragraph, Wrap Around

Standardization

PhenoMeNal has implemented several dedicated Galaxy modules that directly retrieve and store ISA-Tab data set descriptors from and to MetaboLights, and can convert between other formats. Native Galaxy composite data types to support ISA-Tab and ISA-JSON have also been integrated, building upon the ISA API [28,38]. The ISA data type allows for the upload of an ISA-Tab archive (a zip file containing the ISA set of files and raw data when available), which is displayed to the users as a single Galaxy history data set. The integrated Galaxy modules include a MetaboLights downloader and uploader (for ingestion and submission), an ISAcreate module for the creation of ISA compliant archives, modules to explore study metadata through queries on study factors, ISA-Tab “slicing” where queries are used to select subsets of data files of interest, as well as format conversion (export to ISA-JSON and W4M) and study metadata validation (Supplemental Table 1).

PhenoMeNal also advanced the specification of the nmrML standard data format [56] and contributed a dedicated composite data type for nmrML to Galaxy. nmrML is used extensively throughout the NMR 1D workflow and conversion from raw format into nmrML is supported via dedicated Galaxy modules (Table 1).

Throughout the entire analysis pipeline, modules of computational workflows were designed to accept standard formats such as mzML, XML or CSV whenever possible.

Standardized APIs/ABIs are being used for the programmatic interfaces as well as for deploying services. To this end, modern and standardized programming, scripting and meta languages were selected such as Go, HCL, Python, Shell, XML and YAML that are widely used in cloud computing.

Reusability

In an ongoing effort, PhenoMeNal is actively advancing the FAIR criteria for good data management and stewardship [67] to be applied not only to data, but also to software tools and computational workflows (Table 2).

Table 2: Overview of the most important FAIR criteria and implementations suggested for PhenoMeNal data, tools and workflows.

	**Data**	**Tools**	**Workflows**
**(F)indability**	Indexing in domain relevant databases (e.g. MetaboLights)	Indexing in domain relevant software repositories (e.g. the PhenoMeNal App Library, GitHub)	Indexing in workflow management systems such as Galaxy (e.g. PhenoMeNal, W4M), or libraries such as www.myexperiment.org

Formatted: Position Horizontal: Center, Relative to: Margin, Vertical: 0", Relative to: Paragraph, Wrap Around

	Rich descriptions of metadata (e.g. ISA-Tab)	Tool descriptions follow the EDAM ontology	Persistent identifier (e.g. W4M ID, DOI) and intuitive naming patterns
**(A)ccessibility**	Data access and rights management based on e.g. data use ontology (DUO)	Accessible open source licenses	Access to workflow systems can be configured to be shared or restricted
**(I)nteroperability**	Standard formats for experimental metadata (ISA-Tab / ISA-JSON)	Standardized tool descriptions	Standardized workflow format (e.g. Galaxy GA format, Common Workflow Language CWL)
	Domain specific standards for raw data (e.g. mzML, nmrML)	Containerization of software tools	Execution in various software environments (e.g. through the use of containers)
	OboFoundry vocabularies and established domain ontologies to annotate data	EDAM ontology to annotate tools	Workflow annotation ontologies (e.g. Ontology of workflow motifs for annotating workflow specifications [68])
**(R)eusability**	Deposition in data repositories (e.g. MetaboLights) and data indexing sites (e.g. OmicsDI)	Rich documentation and usage guides	Rich documentation and tutorials (e.g. Galaxy tours)

Privacy

PhenoMeNal supports fully anonymized data, which cannot be traced back to individuals in any way [40] and treats pseudonymized data as identifiable. As pseudonymized data are anonymous to the investigator, third parties may be able to link pseudonymized data back to identifiable individuals through mappings such as a hash or code [39]. In these cases, e.g. in a hospital environment, users must deploy PhenoMeNal within a private cloud or bare metal cluster behind their institution's firewall.

PhenoMeNal provides guidance on ethical and technical frameworks to regulate and secure the use of private or sensitive data [39,40]. It is possible to combine data and metadata within an ELSI compliant framework [40] and in such cases users can follow the example of the European Genome Phenome Archive (EGA) [69]. In public installations of

Formatted: Position Horizontal: Center, Relative to: Margin, Vertical: 0", Relative to: Paragraph, Wrap Around

PhenoMeNal, the ELIXIR policy on privacy has been implemented within a technically secure environment to process data [32].

Security

Open source tools are used throughout the entire e-infrastructure and this promotes community efforts to discover and resolve bugs and security issues. The container build process is steered by the continuous integration (CI) service Jenkins, which continuously builds the containers and generates reports. On success and through authentication container images are pushed to the PhenoMeNal container registry which is publicly available but read only. Cloud provider credentials are not stored in the cloud, but only on the deployer host. The Kubernetes cluster running the Jenkins-CI and the container registry, as well as the portal, runs on a CoreOS container, which is a self-updatable, cluster-aware system with most portions being read-only. It reboots nodes sequentially to avoid lack of availability.

KubeNow is a key component that initializes the cloud infrastructure and configures access to it via Cloudflare [70], providing dynamic DNS services and encryption for all network communication. The flexible implementation of PhenoMeNal allows the user to decide to not use CloudFlare in which case encryption is disabled. KubeAdm, which manages the setup of Kubernetes, is not reachable at runtime by default. The only way to access it is by having access to the private key stored on the computer on which it was launched. PhenoMeNal only allows access to standard ports (ssh, http, https and port 44 for the Galaxy Downloader) and implements a cloud-specific firewall for all supported cloud providers.

Microservices are designed to be launched on-demand and terminated after completed analysis. If security issues are reported for the microservices, tools or dependencies, or incremental security patches are available, new builds are automatically triggered in the CI system and developers and the release manager are notified to take additional actions if required. Images are built on a daily basis and tested for deployment, to avoid security patches from introducing any abnormality in the deployment process.

User Resources

A great deal of user resources exist for both PhenoMeNal users and developers, in the form of documentation, tutorials and training videos. The PhenoMeNal Wiki [41] contains detailed documentation on all aspects of PhenoMeNal, including general user guides, workflow and tool tutorials, developer documentation and general information on topics such as security and the e-infrastructure landscape. The PhenoMeNal portal contains help pages generated from the Wiki [71], which are categorized as User Documentation, Developer Documentation and Workflow Tutorials. Interactive Galaxy tours are directly integrated in Galaxy [72]. Training videos are available at the project's YouTube page [73].

Formatted: Position Horizontal: Center, Relative to: Margin, Vertical: 0", Relative to: Paragraph, Wrap Around

Availability of supporting source code and requirements

Project name: PhenoMeNal,
Project home page: http://phenomenal-h2020.eu
Operating system(s): Platform independent
Programming language: Go, HCL, Java, JavaScript, Python, R, Shell, XML, YAML
Other requirements: Linux, Docker, Kubernetes, Terraform, Ansible, Helm
License: MIT license for all code written by the PhenoMeNal project. Individual, Open Source Foundation approved licenses for all containerized tools.
RRID:SCR_016605

Supporting data

The following MetaboLights datasets are integrated into PhenoMeNal and are used to demonstrate the cloud integration and reproducibility of Galaxy workflows: MTBLS1 (NMR1D), MTBLS404 (Uni- and multivariate statistics), MTBLS412 (Fluxomics), MTBLS520 (Eco-Metabolomics), MTBLS558 (MetFrag). Datasets are available at https://www.ebi.ac.uk/metabolights. Snapshots of the code and further supporting data are available in the *GigaScience* repository, GigaDB [74].

Abbreviations

ABI – Application Binary Interface
API – Application Programming Interface
AWS – Amazon Web Services
CI – Continuous Integration
CRE – Cloud Research Environment
DIMS – Direct infusion mass spectrometry
ELSI – Ethical, Legal and Social Implications
FAIR – Criteria for good data management and stewardship based on Findability, Accessibility, Interoperability and Reusability
GC/MS – Gas chromatography coupled with mass spectrometry
GCP – Google Cloud Platform
GDPR – General Data Protection Regulation
GUI – Graphical User Interface
ISA – Investigation, Study and Assay data model framework
IaaS – Infrastructure-as-a-Service
LC/MS – Liquid chromatography coupled with mass spectrometry
MS – Mass Spectrometry
NMR – Nuclear Magnetic Resonance Spectroscopy
PhenoMeNal – Phenome and Metabolome aNalysis
VMI – Virtual Machine Image
W4M – Workflow4Metabolomics

Formatted: Position Horizontal: Center, Relative to: Margin, Vertical: 0", Relative to: Paragraph, Wrap Around

Competing interests

The authors declare that they have no competing interests.

Declarations

Human-derived samples in the data sets MTBLS404 and MTBLS412 were processed according to ELSI guidelines.

Author contributions

Writing original draft: KP and JB.
Conceptualization: CS.
Supervision: RG, ULG, KH, SN, AR, MvR, CS, OS, PR-S, RW.
Project Administration: NK.
Technical lead: PM.
Review and editing: JB, MC, MCap, MCas, PdA, TMDE, RG, AG-B, KH, SH, DJa, DJo, FJ, KK, NK, PEK, AL, SL, PM, SN, COD, KP, LP, MEP, MACR, PR-S, PR-M, AR, RR, CR, MvR, NS, RMS, S-AS, DS, OS, VS, EAT, MT, TH, MvV, MRV, RJMW, GZ, CS.
Software: JB, MCap, MCas, PdA, AG-B, ULG, KH, SH, DJo, FJ, PEK, AL, CL, PM, SN, COD, KP, LP, MEP, MACR, PR-S, PR-M, AR, RR, CR, MvR, NS, RMS, S-AS, OS, VS, EAT, MT, TH, MvV, RJMW, GZ.
External software: SB, CF, EH, SH, MI, DJa, BK, IK, KK, PEK, SL, JAN, JTMP, AP, LP, RR.
Data curation: KH, S-AS, PR-S.
Funding acquisition: RG, ULG, KH, SN, AR, MvR, CS, OS, PR-S, RW.
All authors contributed to, read and approved the final manuscript.

Funding

The project was funded by European Commission PhenoMeNal Grant EC654241. The consortium members JB, Mcap, MCas, PdA, TMDE, RG, AG-B, KH, MI, DJo, FJ, NK, PEK, AL, PM, SN, COD, KP, LP, MACR, PR-S, PR-M, AR, RR, CR, TH, MvR, MvV, NS, RMS, S-AS, DS, OS, VS, EAT, MT, MRV, RJMW, CS received funding from the European Commission PhenoMeNal Grant EC654241.

Formatted: Position Horizontal: Center, Relative to: Margin, Vertical: 0", Relative to: Paragraph, Wrap Around

Supplemental Material

Infrastructure layout

Starting from the IaaS, the first layer is a cluster of Virtual Machines (VMs) which are started and initialized with a defined operating system (from a base image). This is called infrastructure provisioning, and in PhenoMeNal VMs are executed through the Terraform framework [75]. Terraform deploys VM setups to a number of public and private cloud providers including OpenStack, GCE and AWS. The resulting VMs run with a clean install of an operating system including the relevant networking features. KubeAdm manages the setup of these VMs and the Ansible framework is used for the software provisioning layer which performs the deployment of the container daemon and the container orchestrator [76]. Google Kubernetes is used to run software on top of the provisioned VMs [25]. Docker is used as the orchestrator daemon for the containers [77].

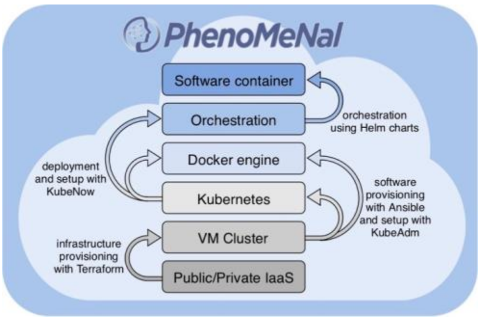

Supplemental Figure 1: PhenoMeNal implements various layers to provision containers on top of the e-infrastructure.

The cloud infrastructure of PhenoMeNal is based upon containers that are deployed in a Kubernetes environment. Deployment is managed by KubeNow, which is developed by the PhenoMeNal team in order to simplify managing the deployment, including storage, network and other required services [25,37]. Orchestration is handled by using Helm charts. The storage subsystem is based on the cloud storage file system GlusterFS. Security is guaranteed via HTTPS encryption (SSL certificates issued by Cloudflare). This elastic implementation allows PhenoMeNal to be instantiated on any Kubernetes-based cloud environment, including bare metal clusters [22]. We use a standardized REST API to operate and communicate between the different interfaces [78].

Formatted: Position Horizontal: Center, Relative to: Margin, Vertical: 0", Relative to: Paragraph, Wrap Around

Standardization through ISA

The ISAcreate module enables the creation of ISA-compliant archives for deposition to repositories such as MetaboLights. The tool presents users with a graphical user interface (GUI) in which to specify study design information such as a treatment plan, sampling and assay plans, as well as QA/QC plans, critical for quality control. During the specification of these plans, the GUI enables semantic markup through the selection of terms chosen from multiple community-based, open ontologies for describing the different components, namely: UBERON ontology for anatomical parts, OBI for experimental protocols [79], MSIO for metabolomics-specific terms and quality control terminology developed by the PhenoMenal project [80,81], DUO for consent and data use terms [82] thereby addressing essential ethical requirements, and STATO for statistical terms (http://www.stato-ontology.org). Based on the combination of the treatment, sampling and assay, and QA/QC plans, the ISA API calculates the experimental graph relationships between subjects, samples, and data files, prospectively. The resulting output is made available as an ISA-Tab history item in Galaxy.

Formatted: Position Horizontal: Center, Relative to: Margin, Vertical: 0", Relative to: Paragraph, Wrap Around

Supplemental Table 1: List of external software tools that were incorporated into PhenoMeNal.

Container Name	Description	URL	Reference
ArtiMID	Corrects mass isotopomer distribution (MID) for natural isotopes abundance, giving artificial MID	https://github.com/phnmnl/container-artimid	[83]
Batch Correction	Corrects intensities for signal drift and batch-effects	https://github.com/phnmnl/container-batch_correction	[62]
BATMAN	Bayesian Automated Metabolite Analyzer for NMR spectra (BATMAN)	https://github.com/phnmnl/container-batman	[84]
Bioconductor	Metabolomics flavors of Bioconductor	https://github.com/phnmnl/bioc_docker	
Biosigner	Discovery of significant signatures from 'omics data	https://github.com/phnmnl/container-biosigner	[85]
Bruker2BATMAN	Converts Bruker raw files into tabulated txt file for BATMAN	https://github.com/phnmnl/container-bruker2batman	[85]
CAMERA	Collection of annotation related methods for mass spectrometry data	https://github.com/phnmnl/container-camera	[86]
CSI:FingerID	A framework for performing metabolomics identification	https://github.com/phnmnl/container-csifingerid	[87]
DIMSpy	Processing, filtering and analyzing direct-infusion mass spectrometry-based metabolomics and lipidomics data	https://github.com/phnmnl/container-dimspy	[88]
EcoMet	Perform diversity and multivariate analyses for eco-metabolomics data	https://github.com/phnmnl/container-ecomet	[63]
Escher Web	A web-based visualization tool for biological pathways	https://github.com/phnmnl/container-escher-fluxomics	[89]
FingerprintClustering	Performs unsupervised clustering and automatically determination of the best number of clusters	https://github.com/phnmnl/container-fingerprintClustering	[90]
FingerprintSubnetwork	Calculates distances between metabolites in a network	https://github.com/phnmnl/container-fingerprintSubnetwork	[90]
Galaxy	PhenoMeNal version of Galaxy as implemented as a container capable of running inside the Kubernetes container orchestrator	https://github.com/phnmnl/container-galaxy-k8s-runtime	
Generic Filter	Allows to remove all samples and/or variables corresponding to specific values regarding designated factors or numerical variables	https://github.com/phnmnl/container-tool-generic_filter	[13]
IPO	A Tool for automated Optimization of XCMS Parameters	https://github.com/phnmnl/container-ipo	[91]

Formatted: Position Horizontal: Center, Relative to: Margin, Vertical: 0", Relative to: Paragraph, Wrap Around

ISA Extractor	ISA data files extractor	https://github.com/phnmnl/container-isa-extractor	[92]
ISA-Tab Slicer	Using the ISA-API for slicing ISA-Tab metadata	https://github.com/phnmnl/container-isaslicer	[93]
ISA-Tab Validator	ISA-Tab validator	https://github.com/phnmnl/container-isatab-validator	[93]
ISA-Tab to JSON Converter	Converts ISA-Tab to JSON data	https://github.com/phnmnl/container-isatab2json	[93]
ISA-Tab to JSON Validator	Create, manipulate and convert ISA-Tab formatted content and produce validation reports on a ISA-JSON formatted document	https://github.com/phnmnl/container-isajson-validator	[93]
ISA-Tab to W4M	ISA to Workflow4Metabolomics converter	https://github.com/phnmnl/container-isa2w4m	[93]
Iso2Flux	Open source software for steady state ^{13}C Metabolic Flux Analysis	https://github.com/phnmnl/container-iso2flux	
IsoDyn	Simulating the dynamics of metabolites and their isotopic isomers in a central metabolic network using a kinetic model	https://github.com/phnmnl/container-isodyn	[94]
JSON to ISA-Tab Converter	Converts JSON to ISA-Tab format	https://github.com/phnmnl/container-json2isatab	[93]
Jupyter	Light-weight flavor (microservice architecture) of Jupyter	https://github.com/phnmnl/container-jupyter	[95]
LCMS matching	Annotation of MS peaks with matching on a spectral database	https://github.com/phnmnl/container-lcmsmatching	
Luigi	Building complex tasks for scientific notebooks and workflows	https://github.com/phnmnl/container-luigi	
MetaboLab	Non-GUI version of MetaboLab - software for processing and analyzing NMR data	https://github.com/phnmnl/container-metabolab	[96]
MetaboliteIDConverter	Enrich metabolomic data sets with well-known databases identifiers such as InChIKey or ChEBI identifiers	https://github.com/phnmnl/container-MetaboliteIDConverter	[97]
Metabomatching	Identifies metabolites in NMR data using regression, correlation, or PCA spiking	https://github.com/phnmnl/container-metabomatching	[98]
MetExplore	Exploration of metabolic networks	https://github.com/phnmnl/container-MetExploreViz	[90]
MetFrag	Annotation of high precision tandem mass spectra of metabolites	https://github.com/phnmnl/container-metfrag-cli, https://github.com/phnmnl/container-metfrag-cli-batch, https://github.com/phnmnl/container-metfrag-vis	[60]

Formatted: Position Horizontal: Center, Relative to: Margin, Vertical: 0", Relative to: Paragraph, Wrap Around

MIDcor	Correcting ^{13}C mass isotopomers spectra of metabolites for natural occurring isotopes and peaks overlapping	https://github.com/phnmnl/container-midcor	[83]
CDF to MIDcor Converter	Converting CDF files into MIDcor to evaluate the mass spectra of ^{13}C-labeled metabolites	https://github.com/phnmnl/container-cdf2mid	[83]
ms-vfetc	Convert MS vendor export formats to a tabular format	https://github.com/phnmnl/container-ms-vfetc	
MSnbase	Basic plotting, data manipulation and processing of MS-based proteomics and metabolomics data	https://github.com/phnmnl/container-msnbase	
MetaboLights Labs Uploader	Facilities uploading data to MetaboLights Labs	https://github.com/phnmnl/container-mtbl-labs-uploader	[6]
MetaboLights ISA slicer	Selecting subsets of data files from ISA-Tab metadata, based on factor values	https://github.com/phnmnl/container-mtblisa	[93]
MetaboLights Downloader	Download a MetaboLights study and output an ISA-Tab data set. Partial downloading of the data is available through a slicing mechanism.	https://github.com/phnmnl/container-mtbls-dwnld	[13]
MetaboLights Factors Visualization	Create parallel sets plots to show factor values distributions in samples inside an ISA-Tab document or MTBLS study	https://github.com/phnmnl/container-mtbls-factors-viz	[93]
Multivariate	PCA, PLS(-DA) and OPLS(-DA) for multivariate analysis of 'omics data	https://github.com/phnmnl/container-multivariate	[13]
MWTab to ISA-Tab Converter	Generate ISA-Tab document from an NIH Metabolomics Workbench study	https://github.com/phnmnl/container-mw2isa	[12]
mzQuality	A tool to assess the quality of targeted mass spectrometry measurements	https://github.com/phnmnl/container-mzquality	
NMR Integrals	Compares specific metabolite levels in two NMR spectra of blood serum/plasma samples.	https://github.com/phnmnl/container-nmr-integrals	
nmrglue	A module for working with NMR data in Python	https://github.com/phnmnl/container-nmrglue	[99]
nmrML to BATMAN Converter	Convert zipped nmrML files into tabulated txt file for BATMAN	https://github.com/phnmnl/container-nmrML2BATMAN	[84]
nmrML to ISA-Tab Metadata	Convert nmrML metadata to ISA-Tab	https://github.com/phnmnl/container-nmrml2isa	[38]
nmrML Converter	Convert RAW vendor NMR files to nmrML	https://github.com/phnmnl/container-nmrmlconv	[27]
nmrPro	Processing and visualization of NMR data	https://github.com/phnmnl/container-nmrpro	[100]
nmrProcFlow + Rnmr1D	An efficient tool for spectra processing from 1D NMR metabolomics data	https://github.com/phnmnl/container-nmrprocflow	[43]
Normalization	Normalization (operation applied on each (preprocessed) individual spectrum) of preprocessed data	https://github.com/phnmnl/container-normalization	[13]

OpenMS	OpenMS open source software library for LC/MS data management and analyses	https://github.com/phnmnl/container-openms	[101]
Pachyderm	A distributed data-processing tool built on software containers that enables scalable and reproducible pipelines	https://github.com/phnmnl/MTBLS233-Pachyderm	[25]
PAPY	Estimation of statistical power and sample size in metabolic phenotyping	https://github.com/phnmnl/container-papy	[102]
Passatutto	Framework for converting metabolomics identification scores to posterior error probability	https://github.com/phnmnl/container-passatutto https://github.com/phnmnl/container-passatuttopep	[103]
PathwayEnrichment	Predict pathway enrichment into a (human) metabolic network	https://github.com/phnmnl/container-pathwayEnrichment	[90]
ProteoWizard MSConvert	Conversion of mass spectrometry vendor formats to mzML	https://github.com/phnmnl/container-pwiz	[104]
Quality Metrics	Metrics and graphics to check the quality of the data	https://github.com/phnmnl/container-qualitymetrics	[13]
RaMID	Evaluate the mass spectra of 13C-labeled metabolites	https://github.com/phnmnl/container-ramid	
rDolphin	Automatic profiling of 1H 1D NMR data sets	https://github.com/phnmnl/container-rdolphin	
reshape2	Performing cast and melt transformation on data matrices	https://github.com/phnmnl/container-reshape2-cast https://github.com/phnmnl/container-reshape2-melt	
rNMR	Identifying and quantifying metabolites in NMR spectra	https://github.com/phnmnl/container-rnmr	[105]
SBML to JSON Converter	Convert SBML files into JSON format useable in the MetExploreViz visualization module	https://github.com/phnmnl/container-SBML2MetexploreJsonGraph	[106]
SCAMID	Extract MID (mass isotopomer distribution) from mass spectra time course of ^{13}C-labeled metabolites files	https://github.com/phnmnl/container-scamid	[83]
SIMID	Evaluate the mass spectra of ^{13}C-labeled metabolites	https://github.com/phnmnl/container-simid	[83]
SOAP-NMR	Perform 1H-NMR data pre-treatment	https://github.com/phnmnl/container-soap-nmr	
Stadyn	Performs simple statistics on individual samples preparing data for simulation with Isodyn	https://github.com/phnmnl/container-stadyn	[83]
tameNMR	Tools for Analysis of MEtabolomic NMR	https://github.com/phnmnl/container-tamenmr	
Transformation	Transform dataMatrix intensity values	https://github.com/phnmnl/container-transformation	[13]

Formatted: Position Horizontal: Center, Relative to: Margin, Vertical: 0", Relative to: Paragraph, Wrap Around

Univariate	Univariate statistics	https://github.com/phnmnl/container-univariate	[13]
XCMS	Framework for processing and visualization of chromatographically separated and single-spectra mass spectral data	https://github.com/phnmnl/container-xcms, https://github.com/phnmnl/container-xcms-1.x	[107]

Formatted: Position Horizontal: Center, Relative to: Margin, Vertical: 0", Relative to: Paragraph, Wrap Around

References

1. Gowda GN, Zhang S, Gu H, Asiago V, Shanaiah N, Raftery D. Metabolomics-based methods for early disease diagnostics. Expert Rev Mol Diagn. 2008;8:617–33.

2. Bundy JG, Davey MP, Viant MR. Environmental metabolomics: a critical review and future perspectives. Metabolomics. 2009;5:3–21.

3. Peters K, Worrich A, Weinhold A, Alka O, Balcke G, Birkemeyer C, et al. Current Challenges in Plant Eco-Metabolomics. Int J Mol Sci. 2018;19:1385.

4. Weber RJM, Lawson TN, Salek RM, Ebbels TMD, Glen RC, Goodacre R, et al. Computational tools and workflows in metabolomics: An international survey highlights the opportunity for harmonisation through Galaxy. Metabolomics 2017;13:12.

5. Joyce AR, Palsson BØ. The model organism as a system: integrating “omics” data sets. Nat Rev Mol Cell Biol. 2006;7:198–210.

6. Haug K, Salek RM, Conesa P, Hastings J, de Matos P, Rijnbeek M, et al. MetaboLights—an open-access general-purpose repository for metabolomics studies and associated meta-data. Nucleic Acids Res. 2013;41:D781–6.

7. Lindon JC, Nicholson JK. The emergent role of metabolic phenotyping in dynamic patient stratification. Expert Opin Drug Metab Toxicol. 2014;10:915–9.

8. Sumner LW, Hall RD. Metabolomics across the globe. Metabolomics. 2013;9:258–64.

9. Rosato A, Tenori L, Cascante M, De Atauri Carulla PR, Martins Dos Santos VAP, Saccenti E. From correlation to causation: analysis of metabolomics data using systems biology approaches. Metabolomics Off J Metabolomic Soc. 2018;14:37.

10. Vignoli A, Ghini V, Meoni G, Licari C, Takis PG, Tenori L, et al. High-throughput metabolomics by 1D NMR. Angew Chem Int Ed Engl. 2018;

11. Goodacre R, Broadhurst D, Smilde AK, Kristal BS, Baker JD, Beger R, et al. Proposed minimum reporting standards for data analysis in metabolomics. Metabolomics. 2007;3:231–41.

12. Sud M, Fahy E, Cotter D, Azam K, Vadivelu I, Burant C, et al. Metabolomics Workbench: An international repository for metabolomics data and metadata, metabolite standards, protocols, tutorials and training, and analysis tools. Nucleic Acids Res. 2016;44:D463–70.

13. Giacomoni F, Le Corguille G, Monsoor M, Landi M, Pericard P, Petera M, et al. Workflow4Metabolomics: a collaborative research infrastructure for computational metabolomics. Bioinformatics. 2015;31:1493–5.

14. Haug K, Salek RM, Steinbeck C. Global open data management in metabolomics. Curr Opin Chem Biol. 2017;36:58–63.

15. Salek RM, Neumann S, Schober D, Hummel J, Billiau K, Kopka J, et al. COordination of Standards in MetabOlomicS (COSMOS): facilitating integrated metabolomics data access. Metabolomics. 2015;11:1587–97.

16. IPCN. International Phenome Centre Network. http://phenomenetwork.org (2018). Accessed 25 Oct 2018.

Formatted: Position Horizontal: Center, Relative to: Margin, Vertical: 0", Relative to: Paragraph, Wrap Around

17. French Ministry of Research, Higher Education and the National Agency for Science. MetaboHUB. http://www.metabohub.fr/metabohub.html (2018). Accessed 25 Oct 2018.

18. Tautenhahn R, Patti GJ, Rinehart D, Siuzdak G. XCMS Online: A Web-Based Platform to Process Untargeted Metabolomic Data. Anal Chem. 2012;84:5035–9.

19. Chong J, Soufan O, Li C, Caraus I, Li S, Bourque G, et al. MetaboAnalyst 4.0: towards more transparent and integrative metabolomics analysis. Nucleic Acids Res. 2018;46:W486–94.

20. Afgan E, Baker D, van den Beek M, Blankenberg D, Bouvier D, Čech M, et al. The Galaxy platform for accessible, reproducible and collaborative biomedical analyses: 2016 update. Nucleic Acids Res. 2016;44:W3–10.

21. PhenoMeNal: The PhenoMeNal Portal. https://portal.phenomenal-h2020.eu (2018). Accessed 25 Oct 2018.

22. Hoffa C, Mehta G, Freeman T, Deelman E, Keahey K, Berriman B, et al. On the Use of Cloud Computing for Scientific Workflows. 2008 IEEE Fourth Int Conf EScience. Indianapolis, IN, USA: IEEE; 2008 [cited 2018 Sep 3]. p. 640–5. Available from: http://ieeexplore.ieee.org/document/4736878/

23. Digan W, Countouris H, Barritault M, Baudoin D, Laurent-Puig P, Blons H, et al. An architecture for genomics analysis in a clinical setting using Galaxy and Docker. GigaScience. 2017;6; doi:10.1093/gigascience/gix099/4557139

24. Goecks J, Nekrutenko A, Taylor J, Galaxy Team T. Galaxy: a comprehensive approach for supporting accessible, reproducible, and transparent computational research in the life sciences. Genome Biol. 2010;11:R86.

25. Novella JA, Khoonsari PE, Herman S, Whitenack D, Capuccini M, Burman J, et al. Container-based bioinformatics with Pachyderm. Wren J, editor. Bioinformatics. 2018; doi:10.1093/bioinformatics/bty699/5068160

26. PhenoMeNal. The Portal App Library. https://portal.phenomenal-h2020.eu/app-library (2018). Accessed 25 Oct 2018.

27. Rocca-Serra P, Salek RM, Arita M, Correa E, Dayalan S, Gonzalez-Beltran A, et al. Data standards can boost metabolomics research, and if there is a will, there is a way. Metabolomics. 2016;12. doi:10.1007/s11306-015-0879-3

28. The OBI Consortium, Smith B, Ashburner M, Rosse C, Bard J, Bug W, et al. The OBO Foundry: coordinated evolution of ontologies to support biomedical data integration. Nat Biotechnol. 2007;25:1251–5.

29. Steinbeck C, Conesa P, Haug K, Mahendraker T, Williams M, Maguire E, et al. MetaboLights: towards a new COSMOS of metabolomics data management. Metabolomics. 2012;8:757–60.

30. Gil Y, Deelman E, Ellisman M, Fahringer T, Fox G, Gannon D, et al. Examining the Challenges of Scientific Workflows. Computer. 2007;40:24–32.

31. Moutsatsos IK, Hossain I, Agarinis C, Harbinski F, Abraham Y, Dobler L, et al. Jenkins-CI, an Open-Source Continuous Integration System, as a Scientific Data and Image-Processing Platform. SLAS Discov Adv Life Sci RD. 2017;22:238–49.

Formatted: Position Horizontal: Center, Relative to: Margin, Vertical: 0", Relative to: Paragraph, Wrap Around

32. van Rijswijk M, Beirnaert C, Caron C, Cascante M, Dominguez V, Dunn WB, et al. The future of metabolomics in ELIXIR. F1000Research. 2017;6:1649.

33. EGI Foundation. EGI: Advanced Computing for Research. https://www.egi.eu (2018). Accessed 25 Oct 2018.

34. INIGO Datacloud. INtegrating Distributed data Infrastructures for Global ExplOitation. https://www.indigo-datacloud.eu (2018). Accessed 25 Oct 2018.

35. Viljoen M, Dutka Ł, Kryza B, Chen Y. Towards European Open Science Commons: The EGI Open Data Platform and the EGI DataHub. Procedia Comput Sci. 2016;97:148–52.

36. Salomoni D, Campos I, Gaido L, Donvito G, Antonacci M, Fuhrman P, et al. INDIGO-Datacloud: foundations and architectural description of a Platform as a Service oriented to scientific computing. ArXiv160309536 Cs. 2016; http://arxiv.org/abs/1603.09536

37. Capuccini M, Larsson A, Carone M, Novella JA, Sadawi N, Gao J, et al. On-Demand Virtual Research Environments using Microservices. ArXiv180506180 Cs. 2018; http://arxiv.org/abs/1805.06180

38. Rocca-Serra P, Brandizi M, Maguire E, Sklyar N, Taylor C, Begley K, et al. ISA software suite: supporting standards-compliant experimental annotation and enabling curation at the community level. Bioinformatics. 2010;26:2354–6.

39. Sariyar M, Schluender I, Smee C, Suhr S. Sharing and Reuse of Sensitive Data and Samples: Supporting Researchers in Identifying Ethical and Legal Requirements. Biopreservation Biobanking. 2015;13:263–70.

40. Heatherly R, Rasmussen LV, Peissig PL, Pacheco JA, Harris P, Denny JC, et al. A multi-institution evaluation of clinical profile anonymization. J Am Med Inform Assoc. 2016;23:e131–7.

41. PhenoMeNal. Wiki. https://github.com/phnmnl/phenomenal-h2020/wiki (2018). Accessed 25 Oct 2018.

42. PhenoMeNal. GitHub Project Repository. https://github.com/phnmnl/ (2018). Accessed 25 Oct 2018.

43. Jacob D, Deborde C, Lefebvre M, Maucourt M, Moing A. NMRProcFlow: a graphical and interactive tool dedicated to 1D spectra processing for NMR-based metabolomics. Metabolomics. 2017;13; doi:10.1007/s11306-017-1178-y

44. PhenoMeNal. Phenome and Metabolome aNalysis. https://phenomenal-h2020.eu (2018). Accessed 25 Oct 2018.

45. PhenoMeNal. Public Galaxy Instance. https://public.phenomenal-h2020.eu (2018). Accessed 25 Oct 2018.

46. Mell PM, Grance T. The NIST definition of cloud computing. In: Gaithersburg, MD: National Institute of Standards and Technology; 2011. Report No.: NIST SP 800-145. Available from: https://nvlpubs.nist.gov/nistpubs/Legacy/SP/nistspecialpublication800-145.pdf

47. PhenoMeNal. Deploy on Microsoft Azure. https://github.com/phnmnl/phenomenal-h2020/wiki/Deploy-on-Microsoft-Azure (2018). Accessed 25 Oct 2018.

Formatted: Position Horizontal: Center, Relative to: Margin, Vertical: 0", Relative to: Paragraph, Wrap Around

48. PhenoMeNal. Deploy on European Open Science Cloud (EOSC). https://github.com/phnmnl/phenomenal-h2020/wiki/Deploy-on-European-Open-Science-Cloud-(EOSC) (2018). Accessed 25 Oct 2018.

49. PhenoMeNal. Deploy on a local server (bare metal). https://github.com/phnmnl/phenomenal-h2020/wiki/Deploy-on-a-local-server-(bare-metal) (2018). Accessed 25 Oct 2018.

50. PhenoMeNal. How to make your software tool available through PhenoMeNal. https://github.com/phnmnl/phenomenal-h2020/wiki/How-to-make-your-software-tool-available-through-PhenoMeNal (2018). Accessed 25 Oct 2018.

51. Nekrutenko A, Taylor J. Next-generation sequencing data interpretation: enhancing reproducibility and accessibility. Nat Rev Genet. 2012;13:667–72.

52. Sloggett C, Goonasekera N, Afgan E. BioBlend: automating pipeline analyses within Galaxy and CloudMan. Bioinformatics. 2013;29:1685–6.

53. Emami Khoonsari P, Moreno P, Bergmann S, Burman J, Capuccini M, Carone M, et al. Interoperable and scalable data analysis with microservices: Applications in Metabolomics. 2018 [cited 2018 Sep 3]; Available from: http://biorxiv.org/lookup/doi/10.1101/213603

54. Thomas K, Benjamin R-K, Fernando P, Brian G, Matthias B, Jonathan F, et al. Jupyter Notebooks – a publishing format for reproducible computational workflows. Stand Alone. 2016;87–90.

55. Lampa S, Alvarsson J, Spjuth O. Towards agile large-scale predictive modelling in drug discovery with flow-based programming design principles. J Cheminformatics. 2016;8. doi:10.1186/s13321-016-0179-6

56. Schober D, Jacob D, Wilson M, Cruz JA, Marcu A, Grant JR, et al. nmrML: A Community Supported Open Data Standard for the Description, Storage, and Exchange of NMR Data. Anal Chem. 2018;90:649–56.

57. Salek RM, Maguire ML, Bentley E, Rubtsov DV, Hough T, Cheeseman M, et al. A metabolomic comparison of urinary changes in type 2 diabetes in mouse, rat, and human. Physiol Genomics. 2007;29:99–108.

58. Buescher JM, Antoniewicz MR, Boros LG, Burgess SC, Brunengraber H, Clish CB, et al. A roadmap for interpreting 13 C metabolite labeling patterns from cells. Curr Opin Biotechnol. 2015;34:189–201.

59. Niedenführ S, Wiechert W, Nöh K. How to measure metabolic fluxes: a taxonomic guide for 13 C fluxomics. Curr Opin Biotechnol. 2015;34:82–90.

60. Ruttkies C, Schymanski EL, Wolf S, Hollender J, Neumann S. MetFrag relaunched: incorporating strategies beyond in silico fragmentation. J Cheminformatics. 2016;8. Available from: http://www.jcheminf.com/content/8/1/3

61. Herman S, Khoonsari PE, Tolf A, Steinmetz J, Zetterberg H, Åkerfeldt T, et al. Integration of magnetic resonance imaging and protein and metabolite CSF measurements to enable early diagnosis of secondary progressive multiple sclerosis. Theranostics. 2018;8:4477–90.

62. Thévenot EA, Roux A, Xu Y, Ezan E, Junot C. Analysis of the Human Adult Urinary Metabolome Variations with Age, Body Mass Index, and Gender by Implementing a Comprehensive Workflow for Univariate and OPLS Statistical Analyses. J Proteome Res. 2015;14:3322–35.

Formatted: Position Horizontal: Center, Relative to: Margin, Vertical: 0", Relative to: Paragraph, Wrap Around

63. Peters K, Gorzolka K, Bruelheide H, Neumann S. Computational workflow to study the seasonal variation of secondary metabolites in nine different bryophytes. Sci Data. 2018;5:180179.

64. PhenoMeNal. Jenkins-CI Instance. http://phenomenal-h2020.eu/jenkins/ (2018). Accessed 25 Oct 2018.

65. PhenoMeNal. Jenkins Guide. https://github.com/phnmnl/phenomenal-h2020/wiki/Jenkins-Guide (2018). Accessed 25 Oct 2018.

66. Piras ME, Pireddu L, Zanetti G. wft4galaxy: a workflow testing tool for galaxy. Bioinformatics. 2017;33:3805–7.

67. Wilkinson MD, Dumontier M, Aalbersberg IjJ, Appleton G, Axton M, Baak A, et al. The FAIR Guiding Principles for scientific data management and stewardship. Sci Data. 2016;3:160018.

68. Cohen-Boulakia S, Belhajjame K, Collin O, Chopard J, Froidevaux C, Gaignard A, et al. Scientific workflows for computational reproducibility in the life sciences: Status, challenges and opportunities. Future Gener Comput Syst. 2017;75:284–98.

69. Lappalainen I, Almeida-King J, Kumanduri V, Senf A, Spalding JD, ur-Rehman S, et al. The European Genome-phenome Archive of human data consented for biomedical research. Nat Genet. 2015;47:692–5.

70. Cloudflare Inc. Cloudflare. https://www.cloudflare.com/ (2018). Accessed 25 Oct 2018.

71. PhenoMeNal. Portal Help. https://portal.phenomenal-h2020.eu/help (2018). Accessed 25 Oct 2018.

72. PhenoMeNal. Interactive Galaxy Tours. https://public.phenomenal-h2020.eu/tours (2018). Accessed 25 Oct 2018.

73. PhenoMeNal. The PhenoMeNal YouTube page. https://www.youtube.com/channel/UCXGAvsVNQk-aUpckjRC8Ang (2018). Accessed 25 Oct 2018.

74. Peters K; Bradbury J; Bergmann S; Capuccini M; Cascante M; Atauri Pd; D Ebbels TM; Foguet C; Glen R; Gonzalez-Beltran A; Günther UL; Handakas E; Hankemeier T; Haug K; Herman S; Holub P; Izzo M; Jacob D; Johnson D; Jourdan F; Kale N; Karaman I; Khalili B; Khonsari PE; Kultima K; Lampa S; Larsson A; Ludwig C; Moreno P; Neumann S; Novella JA; O'Donovan C; Pearce JTM; Peluso A; Piras ME; Pireddu L; Reed MAC; Rocca-Serra P; Roger P; Rosato A; Rueedi R; Ruttkies C; Sadawi N; Salek RM; Sansone S; Selivanov V; Spjuth O; Schober D; Thévenot EA; Tomasoni M; Rijswijk Mv; Vliet Mv; Viant MR; Weber RJM; Zanetti G; Steinbeck C: Supporting data for "PhenoMeNal: Processing and analysis of Metabolomics data in the Cloud" *GigaScience* Database. 2018. http://dx.doi.org/10.5524/100528

75. Brikman Y. Terraform: Writing Infrastructure as Code. Sebastopol: O'Reilly Media; 2017. Available from: http://public.eblib.com/choice/publicfullrecord.aspx?p=4822376

76. Hanwell MD, de Jong WA, Harris CJ. Open chemistry: RESTful web APIs, JSON, NWChem and the modern web application. J Cheminformatics. 2017;9. doi:10.1186/s13321-017-0241-z

77. Newman S. Building microservices: designing fine-grained systems. First Edition. Beijing Sebastopol, CA: O'Reilly Media; 2015.

78. Erl T, editor. SOA with REST: principles, patterns & constraints for building enterprise solutions with REST. Upper Saddle River, NJ: Prentice Hall; 2012.

Formatted: Position Horizontal: Center, Relative to: Margin, Vertical: 0", Relative to: Paragraph, Wrap Around

79. Bandrowski A, Brinkman R, Brochhausen M, Brush MH, Bug B, Chibucos MC, et al. The Ontology for Biomedical Investigations. Xue Y, editor. PLOS ONE. 2016;11:e0154556.

80. Sansone S-A, Rocca-Serra P, Field D, Maguire E, Taylor C, Hofmann O, et al. Toward interoperable bioscience data. Nat Genet. 2012;44:121–6.

81. Sansone S-A, Schober D, Atherton HJ, Fiehn O, Jenkins H, Rocca-Serra P, et al. Metabolomics standards initiative: ontology working group work in progress. Metabolomics. 2007;3:249–56.

82. Dyke SOM, Philippakis AA, Rambla De Argila J, Paltoo DN, Luetkemeier ES, Knoppers BM, et al. Consent Codes: Upholding Standard Data Use Conditions. Barsh GS, editor. PLOS Genet. 2016;12:e1005772.

83. Selivanov VA, Benito A, Miranda A, Aguilar E, Polat IH, Centelles JJ, et al. MIDcor, an R-program for deciphering mass interferences in mass spectra of metabolites enriched in stable isotopes. BMC Bioinformatics. 2017;18. doi:10.1186/s12859-017-1513-3

84. Hao J, Liebeke M, Astle W, De Iorio M, Bundy JG, Ebbels TMD. Bayesian deconvolution and quantification of metabolites in complex 1D NMR spectra using BATMAN. Nat Protoc. 2014;9:1416–27.

85. Rinaudo P, Boudah S, Junot C, Thévenot EA. biosigner: A New Method for the Discovery of Significant Molecular Signatures from Omics Data. Front Mol Biosci. 2016;3. doi:10.3389/fmolb.2016.00026/abstract

86. Kuhl C, Tautenhahn R, Böttcher C, Larson TR, Neumann S. CAMERA: An Integrated Strategy for Compound Spectra Extraction and Annotation of Liquid Chromatography/Mass Spectrometry Data Sets. Anal Chem. 2012;84:283–9.

87. Dührkop K, Shen H, Meusel M, Rousu J, Böcker S. Searching molecular structure databases with tandem mass spectra using CSI:FingerID. Proc Natl Acad Sci. 2015;112:12580–5.

88. Southam AD, Weber RJM, Engel J, Jones MR, Viant MR. A complete workflow for high-resolution spectral-stitching nanoelectrospray direct-infusion mass-spectrometry-based metabolomics and lipidomics. Nat Protoc. 2017;12:255–73.

89. King ZA, Dräger A, Ebrahim A, Sonnenschein N, Lewis NE, Palsson BO. Escher: A Web Application for Building, Sharing, and Embedding Data-Rich Visualizations of Biological Pathways. Gardner PP, editor. PLOS Comput Biol. 2015;11:e1004321.

90. Cottret L, Frainay C, Chazalviel M, Cabanettes F, Gloaguen Y, Camenen E, et al. MetExplore: collaborative edition and exploration of metabolic networks. Nucleic Acids Res. 2018;46:W495–502.

91. Libiseller G, Dvorzak M, Kleb U, Gander E, Eisenberg T, Madeo F, et al. IPO: a tool for automated optimization of XCMS parameters. BMC Bioinformatics. 2015;16. doi:10.1186/s12859-015-0562-8

92. González-Beltrán A, Neumann S, Maguire E, Sansone S-A, Rocca-Serra P. The Risa R/Bioconductor package: integrative data analysis from experimental metadata and back again. BMC Bioinformatics. 2014;15:S11.

93. Sansone S-A, Rocca-Serra P, Field D, Maguire E, Taylor C, Hofmann O, et al. Toward interoperable bioscience data. Nat Genet. 2012;44:121–6.

Formatted: Position Horizontal: Center, Relative to: Margin, Vertical: 0", Relative to: Paragraph, Wrap Around

94. Selivanov VA, Vizán P, Mollinedo F, Fan TW, Lee PW, Cascante M. Edelfosine-induced metabolic changes in cancer cells that precede the overproduction of reactive oxygen species and apoptosis. BMC Syst Biol. 2010;4:135.

95. Perez F, Granger BE. IPython: A System for Interactive Scientific Computing. Comput Sci Eng. 2007;9:21–9.

96. Ludwig C, Günther UL. MetaboLab - advanced NMR data processing and analysis for metabolomics. BMC Bioinformatics. 2011;12:366.

97. Wohlgemuth G, Haldiya PK, Willighagen E, Kind T, Fiehn O. The Chemical Translation Service--a web-based tool to improve standardization of metabolomic reports. Bioinformatics. 2010;26:2647–8.

98. Rueedi R, Mallol R, Raffler J, Lamparter D, Friedrich N, Vollenweider P, et al. Metabomatching: Using genetic association to identify metabolites in proton NMR spectroscopy. Ouzounis CA, editor. PLOS Comput Biol. 2017;13:e1005839.

99. Helmus JJ, Jaroniec CP. Nmrglue: an open source Python package for the analysis of multidimensional NMR data. J Biomol NMR. 2013;55:355–67.

100. Mohamed A, Nguyen CH, Mamitsuka H. NMRPro: an integrated web component for interactive processing and visualization of NMR spectra. Bioinformatics. 2016;32:2067–8.

101. Sturm M, Bertsch A, Gröpl C, Hildebrandt A, Hussong R, Lange E, et al. OpenMS – An open-source software framework for mass spectrometry. BMC Bioinformatics. 2008;9:163.

102. Blaise BJ, Correia G, Tin A, Young JH, Vergnaud A-C, Lewis M, et al. Power Analysis and Sample Size Determination in Metabolic Phenotyping. Anal Chem. 2016;88:5179–88.

103. Scheubert K, Hufsky F, Petras D, Wang M, Nothias L-F, Dührkop K, et al. Significance estimation for large scale metabolomics annotations by spectral matching. Nat Commun. 2017;8. doi:s41467-017-01318-5

104. Chambers MC, Maclean B, Burke R, Amodei D, Ruderman DL, Neumann S, et al. A cross-platform toolkit for mass spectrometry and proteomics. Nat Biotechnol. 2012;30:918–20.

105. Lewis IA, Schommer SC, Markley JL. rNMR: open source software for identifying and quantifying metabolites in NMR spectra. Magn Reson Chem. 2009;47:S123–6.

106. Rodriguez N, Thomas A, Watanabe L, Vazirabad IY, Kofia V, Gómez HF, et al. JSBML 1.0: providing a smorgasbord of options to encode systems biology models: Table 1. Bioinformatics. 2015;31:3383–6.

107. Benton HP, Wong DM, Trauger SA, Siuzdak G. XCMS ^{2} : Processing Tandem Mass Spectrometry Data for Metabolite Identification and Structural Characterization. Anal Chem. 2008;80:6382–9.

Formatted: Position Horizontal: Center, Relative to: Margin, Vertical: 0", Relative to: Paragraph, Wrap Around

Figure 1

Click here to access/download;Figure;Figure_1_draw.io.pdf

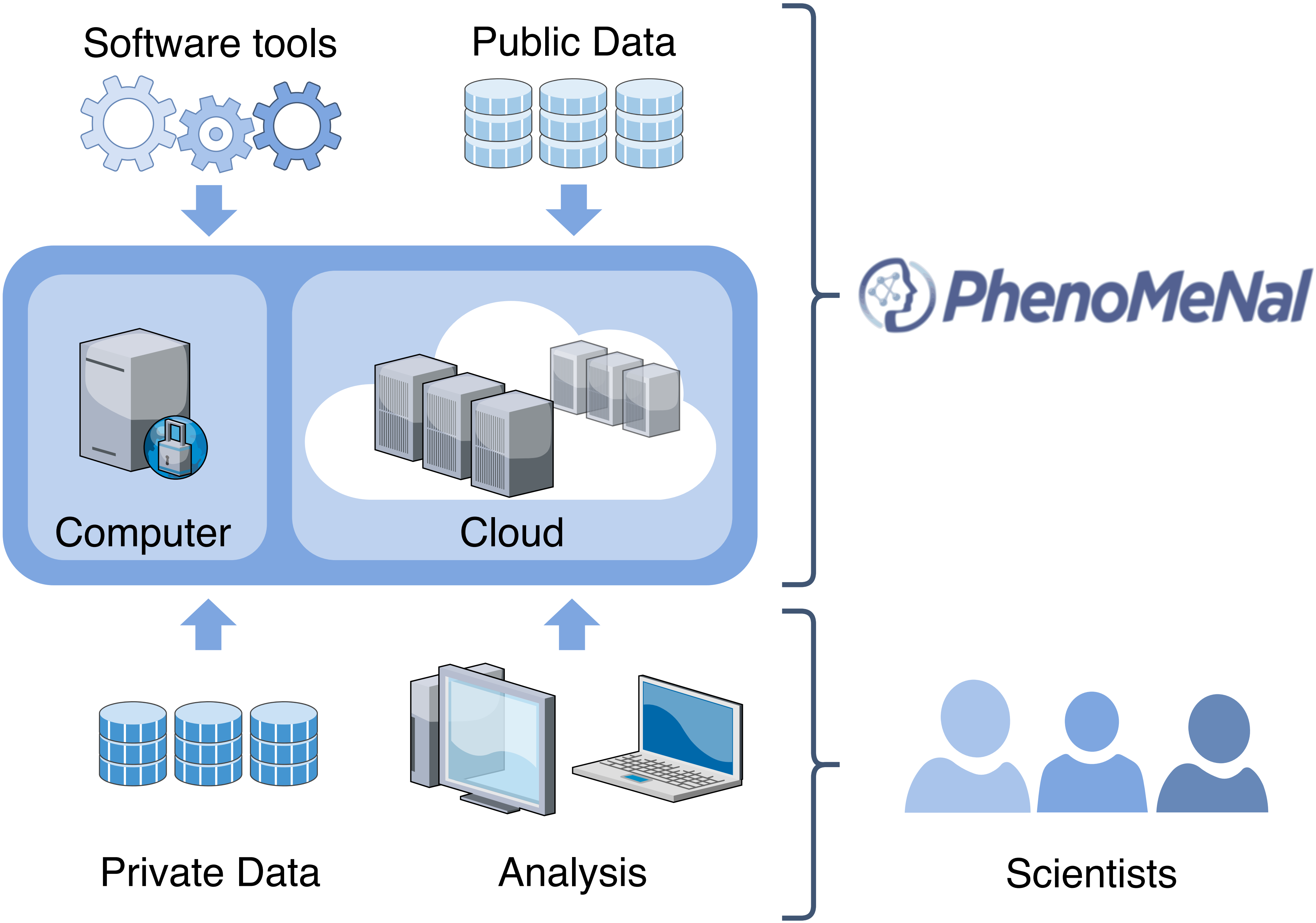

Figure 2

Click here to access/download;Figure;Figure_2.pdf

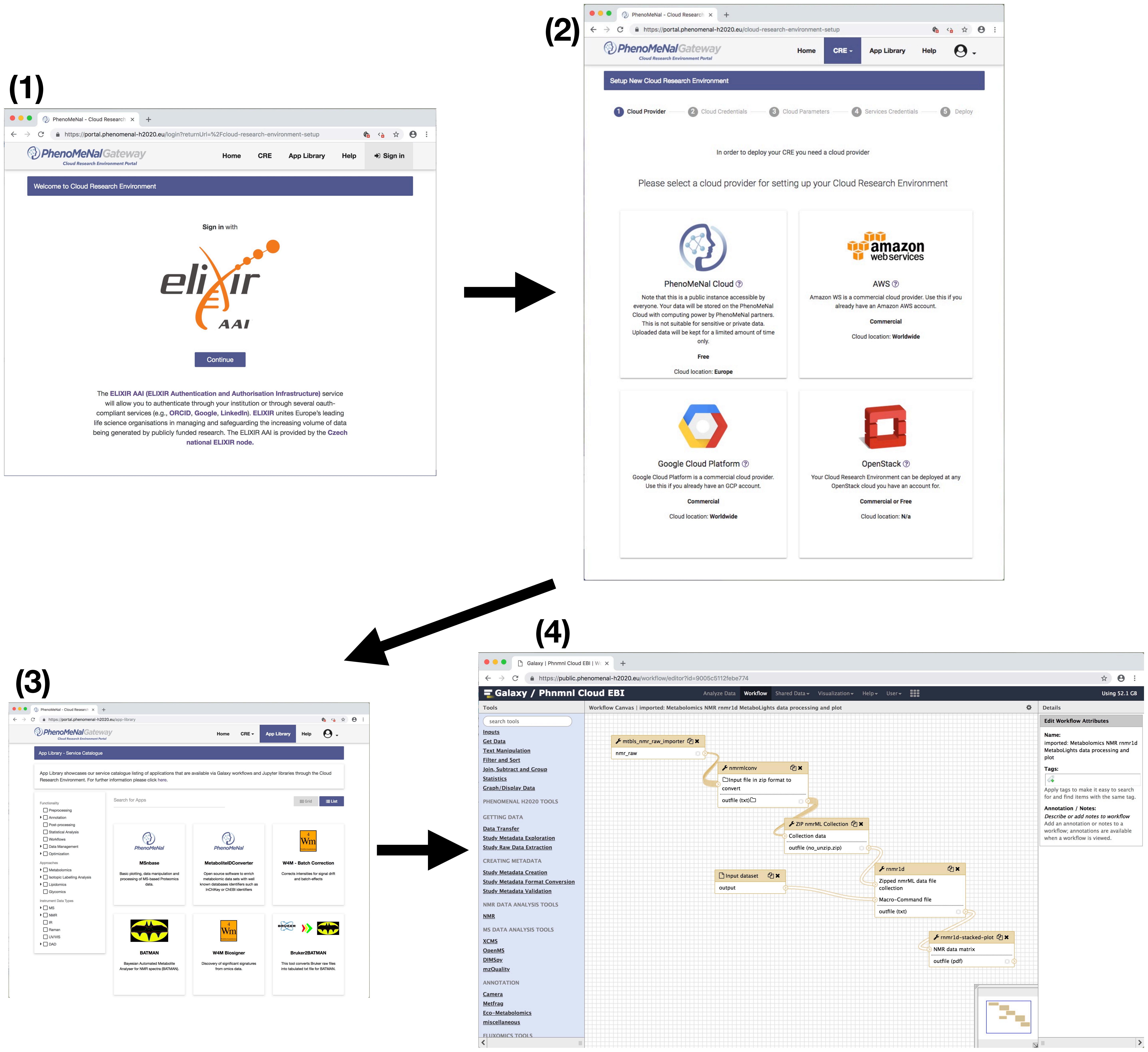

Supplementary Figure 1

Click here to access/download
Supplementary Material
Figure_3_draw.io.pdf

References in RDF format

Click here to access/download
Supplementary Material
phenomenal.rdf

First of all, we thank the reviewers and editors for the very helpful and very proficient comments. We have revised the manuscript according to the comments.

In order to coordinate the revision process between all the involved authors, we have created a Google doc which contains the changes from the individual authors:

https://docs.google.com/document/d/1OlDE-05TFzP6NfITJkMBNm7tAy5J5j9aTfLP6ArUErA/edit

Changes were then transferred to a Word document and the references updated.

Dear Prof. Dr. Steinbeck,

Your manuscript "PhenoMeNal: Processing and analysis of Metabolomics data in the Cloud" (GIGA-D-18-00347) has been assessed by our reviewers. Based on these reports, and my own assessment as Editor, I am pleased to inform you that it is potentially acceptable for publication in GigaScience, once you have carried out some essential revisions suggested by our reviewers.

A comparison is required against other tools such as MetaboAnalyst, XCMS Online, Galaxy and other cloud-based metabolomics tools, as well as including a few sentences to highlight it's uniqueness and novelty would be beneficial.

We have added a comparison in the introduction.

Their reports, together with any other comments, are below. Please also take a moment to check our website at https://giga.editorialmanager.com/ for any additional comments that were saved as attachments.

In addition, please register any new software application in the SciCrunch.org database to receive a RRID (Research Resource Identification Initiative ID) number, and include this in your manuscript. This will facilitate tracking, reproducibility and re-use of your tool.

We have registered the project to SciCrunch.org and have added the ID to the Availability section.

Once you have made the necessary corrections, please submit a revised manuscript online at:

https://giga.editorialmanager.com/

If you have forgotten your username or password please use the "Send Login Details" link to get your login information. For security reasons, your password will be reset.

Please include a point-by-point within the 'Response to Reviewers' box in the submission system. Please ensure you describe additional experiments that were carried out and include a detailed rebuttal of any criticisms or requested revisions that you disagreed with. Please also ensure that your revised manuscript conforms to the journal style, which can be found in the Instructions for Authors on the journal homepage.

The manuscript has been formatted according to the guidelines.

The due date for submitting the revised version of your article is 24 Dec 2018.

We look forward to receiving your revised manuscript soon.

Best wishes,

Nicole Nogoy, Ph.D
GigaScience
www.gigasciencejournal.com

Reviewer reports:
Reviewer #1: Review for PhenoMeNal: Processing and analysis of Metabolomics data in the Cloud

The authors have put together an impressive smorgasbord of software to allow for the data processing of multiple types of metabolomics datasets and continue on with post-processing. Wrapping the Galaxy software into a software-as-a-service system while also integrating other software that may not have been previously integrated into Galaxy. The authors seem to have gone to great lengths to consider open standards and have contacted many universities and institutes.

After reading the notes to authors and reviewers' guidelines it is still difficult to tell if the journal is expecting this type of manuscript. Additionally, due to this being published online I'll use first person.

The authors would like to thank reviewer 1 for the very helpful and valuable comments. We have revised the manuscript according to the comments. The manuscript is intended to be published as a Technical Note in GigaScience. We have formatted the manuscript according to the guidelines appropriate for this publication format.

In general, the manuscript in its current form reads more as a detailed documentation for developers, describing the underlying system. The manuscript is a bit strange in this way that it is presenting a heavy bioinformatic tool with details about company connections and European data regulations that are not often seen in informatic papers.

PhenoMeNal is a comprehensive project with participation of over 50 scientists from different research areas. Thus, PhenoMeNal includes the entire implementation workflow including the technical implementation, reproducibility, sustainability, regulations and ethics. We have revised the manuscript in such a way that also technically/informatically less experienced users understand it. To this end, we have removed very technical parts and added links to documentation in our wiki instead and also moved some informatic parts to the Supplemental.

There is a noticeable lack of comparison against other systems such as MetaboAnalyst, XCMS Online, Galaxy and other cloud-based metabolomics tools.

We have added a comparison to other tools. See our response above.

I would encourage the authors to have a distinct sentence or two saying why the manuscript is novel or why I should use it. I'm very sure that if published it will receive many citations.

The novelty of the project is now specified more clearly in the abstract and throughout the manuscript.

As someone who is already generally familiar with a lot of the discussed underlying technologies it is a difficult read. I would not expect a non-informatic scientist to be able to understand the paper on their initial read. Again, to reiterate the manuscript needs to state why it is publishable.

We have revised the manuscript to be better understandable by scientists who are not bioinformaticians and also removed or relocated very technical parts. However, a certain level of informatic terminology is needed (i.e., discussing the underlying cloud technologies) to meet the requirements of GigaScience.

The abstract findings section is more of methods than what was discovered/found and conclusion does not state why PhenoMeNal is unique in to the aforementioned cloud systems.

The abstract has been rewritten to emphasize the uniqueness of PhenoMeNal.

Major:

1. The authors need to show why the manuscript is novel or what the system brings to the field. There is some attempt to do this via the 2 and ½ page table of programs that can be used however, a more direct comment on this would be very helpful.

The table containing the list of software tools has been moved to the Supplemental as it does not provide key information for the main text of the manuscript. The manuscript has been rewritten to show the uniqueness of PhenoMeNal (see response above).

2. Who is in charge of security checks on all open source apps into phenomenal? As was recently shown with python-pip unless someone is checking each and every app open source software can leak security.

In PhenoMeNal the tool developers and the release manager are in charge of security. They are automatically notified by GitHub on security issues. When security issues are reported, they trigger a new build in our CI system Jenkins and containers are built that contain the latest security patches and also include the latest stable versions from python-pip as dependencies. If security requires explicitly to install new or updated versions, the versions can be adapted in the Dockerfile. A concise version has been added in the security paragraph.

3. Figure 1 for the "today" seems to be very inaccurate again please cite and compare to other preexisting online cloud-based systems.

We have updated Figure 1.

4. What is the phenomal Cloud? How many cores can I allocate to this? How much data can I upload? This isn't discussed much in the documentation - do the authors not want people to use this ?

We are not sure what you mean…. We have specified the nature of PhenoMeNal and compared it to similar solutions in the Findings section. We further pointed out that limits on data storage and cpu cores really depends on the environment PhenoMeNal was deployed on and the parameters that were chosen.

5. The review suggests that figure 2, rather than a screen shot could demonstrate a workflow for the scientific workflow section.

Figure 2 has been redesigned as suggested showing 4 screenshots how to set up a PhenoMeNal e-infrastructure.

6. Reproducibility section a book is cited but a short description of what framework is used here would be nice as the book is rather long and not freely available.

The reference has been updated with an appropriate paper.

7. I noticed that the paper was supported by a European grant named phenomenal and it makes me wonder how long this grant will continue to get funded. I ask only because of the sustainability section. With such a complex system people need to be dedicated to work on this. Many open source projects have become rust-ware, open source does not promise sustainability, simple-ness does. This software contains 9 programming languages and up to 6 platform dependencies.

The European Metabolomics Infrastructure Foundation was recently established through PhenoMeNal project members, that will do maintenance tasks on the developed infrastructure on a best-effort basis. The physical cloud infrastructure required to run PhenoMeNal is independently operated by third parties, including Amazon or Google, or scientific cloud installations like de.NBI or EOSC.

8. Where does the continuous integration happen? Again, this is import for the sustainability!

The Continuous Integration (CI) strategy is implemented in Jenkins-CI. We have added instructions in the Reproducibility section in Methods and linked from the Findings section to make the process more transparent.

9. NeIC-Tryggve2 - a short description of what this is and why it matters to the reader. Google brings up 5 listings for this so very few people probably know about it.

As Tryggve has started as an individual project, we have removed the slightly misleading reference from the manuscript.

10. Methods section is again very informatic heavy. Most scientist will not understand this please make this clearer and help the reader to understand why this is needed.

The methods section has mostly been rewritten for clarity and purpose. Specific informatic topics have been removed to improve the readability so that scientists from other fields do understand the section better.

11. In the scientific workflows the authors add clarity that PhenoMeNal is Galaxy, encapsulated. What does PhenoMeNal do that helps me run Galaxy. I do not feel this has be made clear.

This must be a misunderstanding. In PhenoMeNal, a specific metabolomics “flavour” of Galaxy can be deployed alongside other workflow management systems. The text in the manuscript has been rewritten to make it more concise and understandable.

12. Figure 6 does not add to the understanding of the manuscript. I understand this is digital and colour images are not costly to print however, figures should add content and help the reader to understand.

Figure 6 has been removed as suggested.

13. The manuscript cites that data was used however, I did not see any discussion about data and or processing of that data.

We have added a clarification to the supporting data section.

Minor:

1. I'm unaware of any dataset public or private that are terabytes in size. Many projects with multiple parts including transcriptomics, proteomics, histopathology and others can well exceed the terabytes size but normally it's hundreds of gigabytes. The cited paper talks about file sizes but does not mention datasets. Please find an additional citation if your saying this is in terms of epidemiological studies where there are 1000s of samples.

Phenome Centres process many thousands of metabolite profiles each year. References have been added and the relevant text has been rewritten. Multiple authors are also involved with a large-scale study (which is not published so far) in the field eco-metabolomics that has acquired over 1000 profiles.

2. The authors spend a lot of time talking about how to setup the system on amazon or google both of which can be pricy for academic users. They suggest openstack as a local based alternative. However, many institutes/universities (US based at least) do not run openstack. For an end user this is a lot of configuration to do. What about baremetal, HyperV etc…

The web-based portal supports deployments to AWS, GCE and OpenStack. From the command line, we also support Microsoft Azure, EOSC and bare metal installations. We have added links to our wiki pages which provides step-by-step instructions.

3. A description of what Datacloud and ECI bring to the project and why they are relevant. Many readers may not know

It is beyond the scope of the manuscript to describe these initiatives. We have added qualified references and URLs.

4. The authors cite the recently gone into effect GDPR. This is under the security section and I wonder how this is possible since patients will not know about this system and the metabolomics personal are a rather long way down the line from where the request will happen. Apologizes if I've not fully understood the GDPR.

In PhenoMeNal, GDPR is basically relevant with regard to patient consent. As this is just one minor aspect, explanation of GDPR has been shortened.

5. Table 1 could be in the supplementary. I'm not sure that it adds to the manuscript.

Table 1 has been relocated to the Supplement.

6. I would encourage the use of page numbers

Page numbers have been added to the manuscript.

Reviewer #2:
The authors have presented an exhaustive system that I believe would benefit the Metabolomics community vastly. I am glad to see that PhenoMeNal has taken into consideration the aspects of data openness, data standardisation and security whilst building this system. There are no improvements that I can think of from either a software engineering perspective or from the breadth of usability. I agree that PhenoMeNal is indeed a keystone solution and am looking forward to using it.

The authors would like to thank reviewer 2 for his/her positive feedback.
