# Supplementary figures and images for: PhenoMeNal: processing and analysis of metabolomics data in the cloud
Source: Gigascience. 2018 Dec 7;8(2):giy149. doi: 10.1093/gigascience/giy149 (PMC6377398; doi:10.1093/gigascience/giy149)

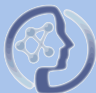

# PhenoMeNal

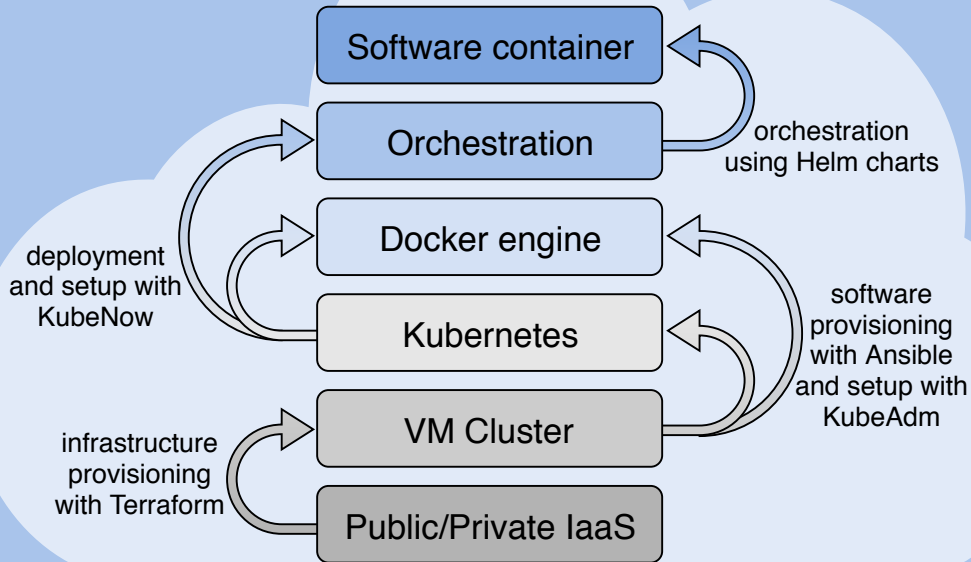

Supplement: Supplemental Files [file giy149_supplemental_files.zip › Figure_3_draw.io.pdf]
