# Supplementary material for: PhenoMeNal: processing and analysis of metabolomics data in the cloud
Source: Gigascience. 2018 Dec 7;8(2):giy149. doi: 10.1093/gigascience/giy149 (PMC6377398; doi:10.1093/gigascience/giy149)
Supplement: Response_to_Reviewer_Comments_Original_Submission.pdf [file giy149_response_to_reviewer_comments_original_submission.pdf]

Author's Response To Reviewer Comments

Close

First of all, we thank the reviewers and editors for the very helpful and very proficient comments. We have revised the manuscript according to the comments.

In order to coordinate the revision process between all the involved authors, we have created a Google doc which contains the changes from the individual authors:

https://docs.google.com/document/d/1OlDE-05TFzP6NfITJkMBNm7tAy5J5j9aTfLP6ArUErA/edit

Changes were then transferred to a Word document and the references updated.

Dear Prof. Dr. Steinbeck,

Your manuscript "PhenoMeNal: Processing and analysis of Metabolomics data in the Cloud" (GIGA-D-18-00347) has been assessed by our reviewers. Based on these reports, and my own assessment as Editor, I am pleased to inform you that it is potentially acceptable for publication in GigaScience, once you have carried out some essential revisions suggested by our reviewers.

A comparison is required against other tools such as MetaboAnalyst, XCMS Online, Galaxy and other cloud-based metabolomics tools, as well as including a few sentences to highlight it's uniqueness and novelty would be beneficial.

We have added a comparison in the introduction.

Their reports, together with any other comments, are below. Please also take a moment to check our website at https://giga.editorialmanager.com/ for any additional comments that were saved as attachments.

In addition, please register any new software application in the SciCrunch.org database to receive a RRID (Research Resource Identification Initiative ID) number, and include this in your manuscript. This will facilitate tracking, reproducibility and re-use of your tool.

We have registered the project to SciCrunch.org and have added the ID to the Availability section.

Once you have made the necessary corrections, please submit a revised manuscript online at:

https://giga.editorialmanager.com/

If you have forgotten your username or password please use the "Send Login Details" link to get your login information. For security reasons, your password will be reset.

Please include a point-by-point within the 'Response to Reviewers' box in the submission

system. Please ensure you describe additional experiments that were carried out and include a detailed rebuttal of any criticisms or requested revisions that you disagreed with. Please also ensure that your revised manuscript conforms to the journal style, which can be found in the Instructions for Authors on the journal homepage.

The manuscript has been formatted according to the guidelines.

The due date for submitting the revised version of your article is 24 Dec 2018.

We look forward to receiving your revised manuscript soon.

Best wishes,

Nicole Nogoy, Ph.D
GigaScience
www.gigasciencejournal.com

Reviewer reports:
Reviewer #1: Review for PhenoMeNal: Processing and analysis of Metabolomics data in the Cloud

The authors have put together an impressive smorgasbord of software to allow for the data processing of multiple types of metabolomics datasets and continue on with post-processing. Wrapping the Galaxy software into a software-as-a-service system while also integrating other software that may not have been previously integrated into Galaxy. The authors seem to have gone to great lengths to consider open standards and have contacted many universities and institutes.

After reading the notes to authors and reviewers' guidelines it is still difficult to tell if the journal is expecting this type of manuscript. Additionally, due to this being published online I'll use first person.

The authors would like to thank reviewer 1 for the very helpful and valuable comments. We have revised the manuscript according to the comments. The manuscript is intended to be published as a Technical Note in GigaScience. We have formatted the manuscript according to the guidelines appropriate for this publication format.

In general, the manuscript in its current form reads more as a detailed documentation for developers, describing the underlying system. The manuscript is a bit strange in this way that it is presenting a heavy bioinformatic tool with details about company connections and European data regulations that are not often seen in informatic papers.

PhenoMeNal is a comprehensive project with participation of over 50 scientists from different research areas. Thus, PhenoMeNal includes the entire implementation workflow including the technical implementation, reproducibility, sustainability, regulations and ethics. We have revised the manuscript in such a way that also technically/informatically less experienced users understand it. To this end, we have removed very technical parts and added links to documentation in our wiki instead and also moved some informatic parts to the Supplemental.

There is a noticeable lack of comparison against other systems such as MetaboAnalyst, XCMS Online, Galaxy and other cloud-based metabolomics tools.

We have added a comparison to other tools. See our response above.

I would encourage the authors to have a distinct sentence or two saying why the manuscript is novel or why I should use it. I'm very sure that if published it will receive many citations.

The novelty of the project is now specified more clearly in the abstract and throughout the manuscript.

As someone who is already generally familiar with a lot of the discussed underlying technologies it is a difficult read. I would not expect a non-informatic scientist to be able to understand the paper on their initial read. Again, to reiterate the manuscript needs to state why it is publishable.

We have revised the manuscript to be better understandable by scientists who are not bioinformaticians and also removed or relocated very technical parts. However, a certain level of informatic terminology is needed (i.e., discussing the underlying cloud technologies) to meet the requirements of GigaScience.

The abstract findings section is more of methods than what was discovered/found and conclusion does not state why PhenoMeNal is unique in to the aforementioned cloud systems.

The abstract has been rewritten to emphasize the uniqueness of PhenoMeNal.

Major:
1. The authors need to show why the manuscript is novel or what the system brings to the field. There is some attempt to do this via the 2 and ½ page table of programs that can be used however, a more direct comment on this would be very helpful.

The table containing the list of software tools has been moved to the Supplemental as it does not provide key information for the main text of the manuscript. The manuscript has been rewritten to show the uniqueness of PhenoMeNal (see response above).

2. Who is in charge of security checks on all open source apps into phenomenal? As was recently shown with python-pip unless someone is checking each and every app open source software can leak security.

In PhenoMeNal the tool developers and the release manager are in charge of security. They are automatically notified by GitHub on security issues. When security issues are reported, they trigger a new build in our CI system Jenkins and containers are built that contain the latest security patches and also include the latest stable versions from python-pip as dependencies. If security requires explicitly to install new or updated versions, the versions can be adapted in the Dockerfile. A concise version has been added in the security paragraph.

3. Figure 1 for the "today" seems to be very inaccurate again please cite and compare to other preexisting online cloud-based systems.

We have updated Figure 1.

4. What is the phenomal Cloud? How many cores can I allocate to this? How much data can I upload? This isn't discussed much in the documentation - do the authors not want people to use this ?

We are not sure what you mean…. We have specified the nature of PhenoMeNal and compared it to similar solutions in the Findings section. We further pointed out that limits on data storage and cpu cores really depends on the environment PhenoMeNal was deployed on and the parameters that were chosen.

5. The review suggests that figure 2, rather than a screen shot could demonstrate a workflow for the scientific workflow section.

Figure 2 has been redesigned as suggested showing 4 screenshots how to set up a PhenoMeNal e-infrastructure.

6. Reproducibility section a book is cited but a short description of what framework is used here would be nice as the book is rather long and not freely available.

The reference has been updated with an appropriate paper.

7. I noticed that the paper was supported by a European grant named phenomenal and it makes me wonder how long this grant will continue to get funded. I ask only because of the sustainability section. With such a complex system people need to be dedicated to work on this. Many open source projects have become rust-ware, open source does not promise sustainability, simple-ness does. This software contains 9 programming languages and up to 6 platform dependencies.

The European Metabolomics Infrastructure Foundation was recently established through PhenoMeNal project members, that will do maintenance tasks on the developed infrastructure on a best-effort basis. The physical cloud infrastructure required to run PhenoMeNal is independently operated by third parties, including Amazon or Google, or scientific cloud installations like de.NBI or EOSC.

8. Where does the continuous integration happen? Again, this is import for the sustainability!

The Continuous Integration (CI) strategy is implemented in Jenkins-CI. We have added instructions in the Reproducibility section in Methods and linked from the Findings section to make the process more transparent.

9. NelC-Tryggve2 - a short description of what this is and why it matters to the reader. Google brings up 5 listings for this so very few people probably know about it.

As Tryggve has started as an individual project, we have removed the slightly misleading reference from the manuscript.

10. Methods section is again very informatic heavy. Most scientist will not understand this please make this clearer and help the reader to understand why this is needed.

The methods section has mostly been rewritten for clarity and purpose. Specific informatic topics have been removed to improve the readability so that scientists from other fields do understand the section better.

11. In the scientific workflows the authors add clarity that PhenoMeNal is Galaxy, encapsulated. What does PhenoMeNal do that helps me run Galaxy. I do not feel this has be made clear.

This must be a misunderstanding. In PhenoMeNal, a specific metabolomics “flavour” of Galaxy can be deployed alongside other workflow management systems. The text in the manuscript has been rewritten to make it more concise and understandable.

12. Figure 6 does not add to the understanding of the manuscript. I understand this is digital and colour images are not costly to print however, figures should add content and help the reader to understand.

Figure 6 has been removed as suggested.

13. The manuscript cites that data was used however, I did not see any discussion about data and or processing of that data.

We have added a clarification to the supporting data section.

Minor:
1. I'm unaware of any dataset public or private that are terabytes in size. Many projects with multiple parts including transcriptomics, proteomics, histopathology and others can well exceed the terabytes size but normally it's hundreds of gigabytes. The cited paper talks about file sizes but does not mention datasets. Please find an additional citation if your saying this is in terms of epidemiological studies where there are 1000s of samples.

Phenome Centres process many thousands of metabolite profiles each year. References have been added and the relevant text has been rewritten. Multiple authors are also involved with a large-scale study (which is not published so far) in the field eco-metabolomics that has acquired over 1000 profiles.

2. The authors spend a lot of time talking about how to setup the system on amazon or google both of which can be pricy for academic users. They suggest openstack as a local based alternative. However, many institutes/universities (US based at least) do not run openstack. For an end user this is a lot of configuration to do. What about baremetal, HyperV etc…

The web-based portal supports deployments to AWS, GCE and OpenStack. From the command line, we also support Microsoft Azure, EOSC and bare metal installations. We have added links to our wiki pages which provides step-by-step instructions.

3. A description of what Datacloud and ECI bring to the project and why they are relevant. Many readers may not know

It is beyond the scope of the manuscript to describe these initiatives. We have added qualified references and URLs.

4. The authors cite the recently gone into effect GDPR. This is under the security section and I wonder how this is possible since patients will not know about this system and the metabolomics personal are a rather long way down the line from where the request will happen. Apologizes if I've not fully understood the GDPR.

In PhenoMeNal, GDPR is basically relevant with regard to patient consent. As this is just one minor aspect, explanation of GDPR has been shortened.

5. Table 1 could be in the supplementary. I'm not sure that it adds to the manuscript.

Table 1 has been relocated to the Supplement.

6. I would encourage the use of page numbers

Page numbers have been added to the manuscript.

Reviewer #2:
The authors have presented an exhaustive system that I believe would benefit the Metabolomics community vastly. I am glad to see that PhenoMeNal has taken into consideration the aspects of data openness, data standardisation and security whilst building this system. There are no improvements that I can think of from either a software engineering perspective or from the breadth of usability. I agree that PhenoMeNal is indeed a keystone solution and am looking forward to using it.

The authors would like to thank reviewer 2 for his/her positive feedback.

Close
