# Supplementary material for: PhenoMeNal: processing and analysis of metabolomics data in the cloud
Source: Gigascience. 2018 Dec 7;8(2):giy149. doi: 10.1093/gigascience/giy149 (PMC6377398; doi:10.1093/gigascience/giy149)
Supplement: Reviewer_1_Report_Original_Submission -- H Paul Benton — 9/24/2018 Reviewed [file giy149_reviewer_1_report_original_submission.pdf]

Reviewer Report

Title: PhenoMeNal: Processing and analysis of Metabolomics data in the Cloud

Version: Original Submission **Date:** 9/24/2018

Reviewer name: H Paul Benton

Reviewer Comments to Author:

Review for PhenoMeNal: Processing and analysis of Metabolomics data in the CloudThe authors have put together an impressive smorgasbord of software to allow for the data processing of multiple types of metabolomics datasets and continue on with post-processing. Wrapping the Galaxy software into a software-as-a-service system while also integrating other software that may not have been previously integrated into Galaxy. The authors seem to have gone to great lengths to consider open standards and have contacted many universities and institutes. After reading the notes to authors and reviewers' guidelines it is still difficult to tell if the journal is expecting this type of manuscript. Additionally, due to this being published online I'll use first person. In general, the manuscript in its current form reads more as a detailed documentation for developers, describing the underlying system. The manuscript is a bit strange in this way that it is presenting a heavy bioinformatic tool with details about company connections and European data regulations that are not often seen in informatic papers. There is a noticeable lack of comparison against other systems such as MetaboAnalyst, XCMS Online, Galaxy and other cloud-based metabolomics tools. I would encourage the authors to have a distinct sentence or two saying why the manuscript is novel or why I should use it. I'm very sure that if published it will receive many citations. As someone who is already generally familiar with a lot of the discussed underlying technologies it is a difficult read. I would not expect a non-informatic scientist to be able to understand the paper on their initial read. Again, to reiterate the manuscript needs to state why it is publishable. The abstract findings section is more of methods than what was discovered/found and conclusion does not state why PhenoMeNal is unique in to the aforementioned cloud systems.Major:1. The authors need to show why the manuscript is novel or what the system brings to the field. There is some attempt to do this via the 2 and ½ page table of programs that can be used however, a more direct comment on this would be very helpful.2. Who is in charge of security checks on all open source apps into phenomenal? As was recently shown with python-pip unless someone is checking each and every app open source software can leak security.3. Figure 1 for the "today" seems to be very inaccurate again please cite and compare to other preexisting online cloud-based systems.4. What is the phenomal Cloud? How many cores can I allocate to this? How much data can I upload? This isn't discussed much in the documentation - do the authors not want people to use this ?5. The review suggests that figure 2, rather than a screen shot could demonstrate a workflow for the scientific workflow section. 6. Reproducibility section a book is cited but a short description of what framework is used here would be nice as the book is rather long and not freely available.7. I noticed that the paper was supported by a European grant named phenomenal and it makes me wonder how long this grant will continue to get funded. I ask only because of the sustainability section. With such a complex system people need to be dedicated to work on this. Many open source projects have become rust-ware, open source does not promise sustainability, simple-ness does. This software contains 9 programming languages and up to 6 platform dependencies. 8. Where does the continuous integration happen? Again, this is import for the sustainability!9. NeIC-Tryggve2 - a short description of what this is and why it matters to the reader. Google brings up 5 listings for this so very few people probably know about it. 10. Methods section is again very informatic heavy. Most scientist will not understand this please make this clearer and help the reader to understand why this is needed.11. In the scientific workflows the authors add clarity that PhenoMeNal is Galaxy, encapsulated. What does PhenoMeNal do that helps me run Galaxy. I do not feel this has be made clear.12. Figure 6 does not add to the understanding of the manuscript. I understand this is digital and colour images are not costly to print however, figures should add content and help the reader to understand.13. The manuscript cites that data was used however, I did not see any discussion about data

and or processing of that data.Minor:1. I'm unaware of any dataset public or private that are terabytes in size. Many projects with multiple parts including transcriptomics, proteomics, histopathology and others can well exceed the terabytes size but normally it's hundreds of gigabytes. The cited paper talks about file sizes but does not mention datasets. Please find an additional citation if your saying this is in terms of epidemiological studies where there are 1000s of samples. 2. The authors spend a lot of time talking about how to setup the system on amazon or google both of which can be pricy for academic users. They suggest openstack as a local based alternative. However, many institutes/universities (US based at least) do not run openstack. For an end user this is a lot of configuration to do. What about baremetal, HyperV etc…3. A description of what Datacloud and ECI bring to the project and why they are relevant. Many readers may not know4. The authors cite the recently gone into effect GDPR. This is under the security section and I wonder how this is possible since patients will not know about this system and the metabolomics personal are a rather long way down the line from where the request will happen. Apologizes if I've not fully understood the GDPR.5. Table 1 could be in the supplementary. I'm not sure that it adds to the manuscript.6. I would encourage the use of page numbers

Level of Interest

Please indicate how interesting you found the manuscript: Choose an item.

Quality of Written English

Please indicate the quality of language in the manuscript: Choose an item.

Declaration of Competing Interests

Please complete a declaration of competing interests, considering the following questions:

- Have you in the past five years received reimbursements, fees, funding, or salary from an organisation that may in any way gain or lose financially from the publication of this manuscript, either now or in the future?
- Do you hold any stocks or shares in an organisation that may in any way gain or lose financially from the publication of this manuscript, either now or in the future?
- Do you hold or are you currently applying for any patents relating to the content of the manuscript?
- Have you received reimbursements, fees, funding, or salary from an organization that holds or has applied for patents relating to the content of the manuscript?
- Do you have any other financial competing interests?
- Do you have any non-financial competing interests in relation to this paper?

If you can answer no to all of the above, write 'I declare that I have no competing interests' below. If your reply is yes to any, please give details below.

Author 7 was my PhD advisor. I help to run one of the worlds largest metabolomic cloud resources.

I agree to the open peer review policy of the journal. I understand that my name will be included on my report to the authors and, if the manuscript is accepted for publication, my named report including any attachments I upload will be posted on the website along with the authors' responses. I agree for my report to be made available under an Open Access Creative Commons CC-BY license (http://creativecommons.org/licenses/by/4.0/). I understand that any comments which I do not wish to be included in my named report can be included as confidential comments to the editors, which will not be published.

I agree to the open peer review policy of the journal

To further support our reviewers, we have joined with Publons, where you can gain additional credit to further highlight your hard work (see: https://publons.com/journal/530/gigascience). On publication of this paper, your review will be automatically added to Publons, you can then choose whether or not to claim your Publons credit. I understand this statement. Yes
